# Patterns of Combined Prenatal Supplement Use and Associations with Birth Outcomes and Early Infant Growth: A Multicenter Prospective Mother–Infant Cohort Study in China

**DOI:** 10.3390/nu18213649

**Published:** 2026-11-02

**Authors:** Haoquan Zhang, Zheng Bian, Yongyi Zhang, Yuchen Wu, Zihan Xiong, Ahenaer Teliewubieerde, Weijie Cai, Siliang Duan, Yuanyuan Yan, Hui Wang

**Affiliations:** 1 School of Public Health, Shanghai Jiao Tong University School of Medicine, Shanghai 200025, China; zhq120805@sjtu.edu.cn (H.Z.); 1715zyy@sjtu.edu.cn (Y.Z.); clearhate@sjtu.edu.cn (Y.W.); xearz1h2237@163.com (Z.X.); ahnar123@sjtu.edu.cn (A.T.); 2 Tongren Hospital, Shanghai Jiao Tong University School of Medicine, 1111 Xianxia Road, Shanghai 200336, China; jacqulingbz@hotmail.com; 3 School of Basic Medicine Sciences, Gannan Medical University, Ganzhou 341000, China; wr2258000839@outlook.com (W.C.); dslxhlhx@163.com (S.D.); 4 Hainan International Medical Center, Shanghai Jiao Tong University School of Medicine, Qionghai 571400, China; 5 Center for Single-Cell Omics, School of Public Health, Shanghai Jiao Tong University School of Medicine, Shanghai 200025, China

**Keywords:** prenatal supplements, combined supplement-use patterns, latent class analysis, birth outcomes, neonatal outcomes, infant growth

## Abstract

Background: Women often use several prenatal supplements together, yet most studies evaluate products one at a time. Objective: To identify longitudinal patterns of combined prenatal supplement use and assess their associations with cesarean delivery, birth size, jaundice, and early infant growth. Methods: This multicenter cohort included 1758 mother–infant pairs from 16 provinces in China. Supplement use was reported once in the baseline questionnaire: on or before delivery for 876 women (median gestational age 38.57 weeks) and after delivery for 809. For each of seven supplements, use frequency was reported once, and participants indicated the pregnancy periods during which the supplement was used. One frequency was assigned to every period endorsed from preconception through the third trimester, giving 28 indicators for latent class analysis. Associations were estimated in exploratory multivariable models, with Benjamini–Hochberg correction within outcome families. Results: Six patterns were identified. They differ in product combination, endorsed periods, and one recorded frequency per product, and are not ordered by dose (C6 (low use), 25.7%; C4 (all-supplement high-frequency), 7.0%). Membership differed by region, parity, and pregnancy complications. Compared with C6, mid-late high-frequency core use (C5) was nominally associated with lower odds of cesarean delivery (adjusted OR 0.72, 95% CI 0.53–0.99). Persistent multi-supplement high-frequency use (C3) and all-supplement high-frequency use (C4) were nominally associated with higher odds of physiological jaundice recorded at the 40–60-day follow-up (1.65, 1.00–2.70 and 1.86, 1.04–3.27), and C4 with shorter birth length (adjusted β −0.46 cm, 95% CI −0.81 to −0.10). These associations did not remain significant after within-family correction. Macrosomia, infant eczema, and gains in length and weight showed no association. Greater head-circumference gain in mid-late moderate core use (C1) remained significant after correction (adjusted β 0.87 cm, 95% CI 0.34–1.40; q = 0.019) among 513 infants in the adjusted analysis. Conclusions: Supplement use clustered into six patterns of timing and reported frequency. The associations are observational and do not show that changing a supplement regimen would change these endpoints.

## 1. Introduction

Micronutrient inadequacy remains highly prevalent among women of reproductive age, with an estimated two-thirds of this population worldwide deficient in at least one key micronutrient [1], and pregnancy further increases the demand for vitamins and minerals. Insufficient micronutrient status during pregnancy has been implicated in adverse maternal and fetal outcomes, including congenital anomalies, maternal anemia, and hypertensive disorders of pregnancy [2]. Prenatal dietary supplements are therefore widely used during pregnancy. Folic acid is firmly recommended for the prevention of neural tube defects [3], while iron and calcium supplementation are routinely recommended in many settings [2,4,5]. In China, prenatal supplement use is widespread and continues to increase, with most pregnant women now reporting use of at least one supplement [6,7]. Therefore, understanding how multiple supplements are combined and used over the course of pregnancy in routine care, and how these real-world use patterns relate to maternal and infant health, is essential for generating evidence to guide prenatal supplementation practice.

Although the benefits of preventing and correcting micronutrient deficiencies are well established, evidence regarding the broader effects of prenatal supplementation on maternal and infant outcomes remains heterogeneous. Multiple-micronutrient and lipid-based nutrient supplementation have been shown to reduce low birth weight, preterm birth, and small-for-gestational-age birth in trials conducted in low- and middle-income settings [8,9]. Omega-3 fatty acid supplementation may reduce preterm birth and increase birth weight [10]. By contrast, prenatal docosahexaenoic acid (DHA) supplementation has not consistently demonstrated benefit for offspring neurodevelopment [11]. Similarly, vitamin D supplementation has shown no clear effect on congenital anomalies [12], and the reported associations between maternal folic acid supplementation and congenital heart disease have demonstrated substantial heterogeneity across studies [13]. Moreover, the effects of multiple-micronutrient supplementation appear to depend on the timing of initiation, duration of use, and adherence. Notably, most of this literature evaluates supplements one product at a time. That approach can overlook jointly occurring regimens, period-specific intensity of use, and the possibility that intensive multi-product use marks clinical indication rather than a single causal exposure.

In China, concurrent use of multiple products during pregnancy is common. In one recent survey, nearly 95% of women reported at least one supplement and almost 30% reported more than four [7], and use varies markedly by region, education, and parity [6,7]. Person-centered approaches are well established in nutrition research: latent class analysis (LCA) has been applied to characterize dietary and consumption patterns in pregnancy [14,15]. Unlike conventional variable-centered approaches, which estimate associations for individual exposures, LCA can capture correlated behaviors and identify empirically derived subgroups characterized by distinct combinations of exposures [16]. Pregnancy dietary patterns and dietary diversity have in turn been associated with gestational diabetes mellitus (GDM), hypertensive disorders, and birth outcomes [17,18]. Applying a similar framework to prenatal supplement use offers an opportunity to move beyond the question of whether a specific supplement is used toward understanding the broader behavioral patterns through which supplementation occurs across pregnancy.

Nevertheless, analogous models of supplement-use patterns remain uncommon. Existing studies in China linking supplementation with birth outcomes still examine products or fortified formulas individually [19], and data connecting real-world use patterns to maternal–infant health indicators, especially beyond the immediate neonatal period, are still scarce. Understanding these relationships in a prospective mother–infant cohort may therefore provide a more comprehensive view of the real-world role of prenatal supplementation.

In the present study, drawing on a multicenter prospective cohort of 1758 mother–infant pairs from 16 provinces in China, we used the LCA of 28 period-by-supplement indicators to identify patterns of prenatal supplement use from preconception through pregnancy: for each supplement, the single reported frequency was assigned to every endorsed period, and a period that was not endorsed was coded 0. We examined sociodemographic and clinical determinants associated with pattern membership and evaluated the associations of these patterns with cesarean delivery and selected neonatal and early postnatal outcomes. We further explored whether pattern membership tracks pregnancy complications, given that clinical indications may influence both supplementation practices and pregnancy outcomes. By shifting the analytical focus from individual supplements to patterns of combined use across pregnancy, this study records which products were used together, which periods were endorsed, and the one frequency reported for each product.

## 2. Materials and Methods

### 2.1. Study Design and Participants

**Figure 1:**
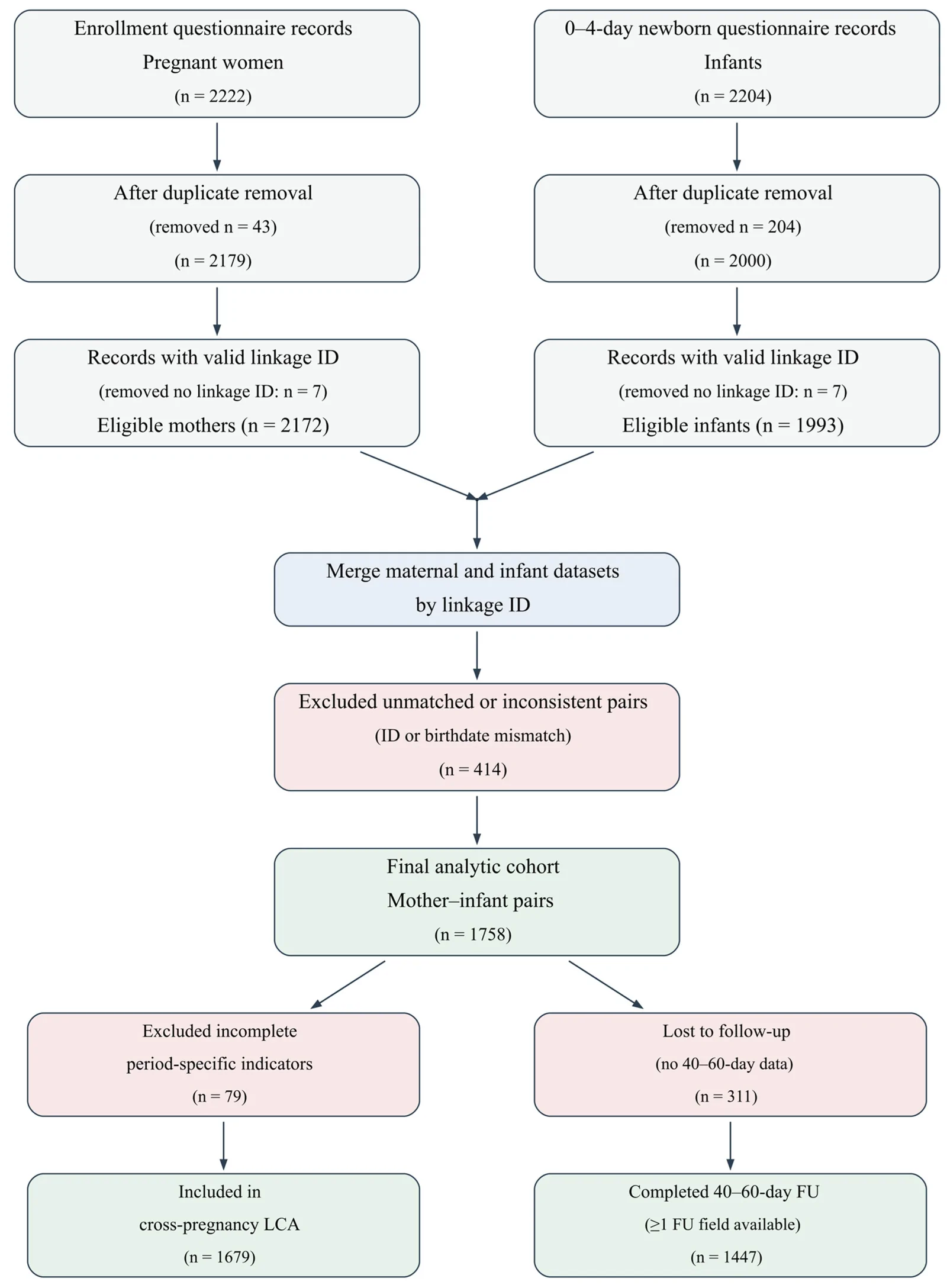
Flow chart of the study population.

This multicenter prospective cohort was conducted at 22 maternal and child health hospitals in 16 provinces of China between November 2023 and December 2025. The questionnaire cohort is the follow-up subset of a mother–infant study that planned breast-milk collection across 30 provinces and municipalities. Milk composition is not an endpoint of this analysis. Sample size used the mean total nitrogen of mature milk at about 4 months (0.170 ± 0.032 g/100 mL) [20]. With a relative margin of 10% and a two-sided 95% confidence level, the sample-size formula for estimating a mean gave about 14 participants per stratum [21]. To allow for 50–60% loss from low milk volume, expression difficulty, and attrition, 35 participants were planned per stratum, so that at least 14 would remain after 60% loss. Three economic strata in each of 30 provinces or municipalities (3150), plus 35 vegetarian mothers, gave a planned sample of 3185. The latent class analysis of supplement-use patterns and the pattern–outcome comparisons in this manuscript are a secondary exploratory analysis of questionnaire and outcome data collected in the cohort. No sample-size calculation was performed for these comparisons. Women were eligible when gestational age at birth was 37 weeks + 0 days to 41 weeks + 6 days and maternal age was 20–40 years. They had no contraindication to breastfeeding, had a strong intention to breastfeed, were willing to return to the delivery hospital for the postpartum examination and child health care, and were proficient with a smartphone. The protocol excluded women with severe nutritional or genetic disease, severe obstetric comorbidity or complication, or endocrine disease affecting breastfeeding, and women who did not later attend the postpartum examination and child health care at the participating hospital. Mother–infant pairs were assembled from the maternal enrollment file and the 0–4-day newborn file, and the baseline questionnaire was completed once (Figure 1). Of 2222 maternal records, 43 duplicates and then 7 records without a linkage identifier were removed, leaving 2172. Of 2204 newborn records, 204 duplicates and then 7 records without a linkage identifier were removed, leaving 1993. A pair was kept only when the identifier matched and the birth date agreed, which removed 414 maternal and 235 newborn records. The analytic cohort was the remaining 1758 pairs. Pairs were not further excluded for preterm birth, missing gestational age, or non-attendance at the 40–60-day visit.

The study was conducted in accordance with the principles of the Declaration of Helsinki and reported according to the Strengthening the Reporting of Observational Studies in Epidemiology (STROBE) guideline [22].

### 2.2. Data Collection

One electronic questionnaire recorded sociodemographics, health status, supplement use before and during pregnancy, and pregnancy-related factors. Preconception use and use in each trimester were reported at that single completion. Newborn data came from a standardized 0–4-day questionnaire, with follow-up at 40–60 days. Participants completed the questionnaires on mobile devices through platform links, and responses were uploaded in real time.

### 2.3. Assessment of Prenatal Supplement Use

The primary exposure was dietary supplement use before and during pregnancy. Latent class analysis used seven supplements with timing data: folic acid, calcium, iron, DHA, fish oil, vitamin C, and vitamin E. Use was assessed once at baseline, not separately for each period. For each product, women reported one frequency: none or almost none, 1–3 times per week, 4–7 times per week, or ≥2 times per day. They also marked preconception and the first, second, and/or third trimester. That frequency was assigned to every endorsed period, and non-use was coded 0, giving 28 ordinal indicators (4 periods × 7 supplements) [23]. Within a woman, frequency did not vary across endorsed periods, so period differences show which periods were endorsed. Zinc, selenium, and multiple-micronutrient supplements (MMS) were summarized descriptively and kept for supplementary product-level analyses, but incomplete timing excluded them from the primary model.

### 2.4. Maternal and Infant Outcomes

Cesarean delivery and macrosomia came from hospital records. Macrosomia was a birth weight of 4000 g or greater. At 40–60 days, mothers reported jaundice from birth to that visit, with onset and resolution, but not a bilirubin value. None, physiological, and pathological were the pediatric categories from the routine examination, entered by the mother, and the study team did not measure bilirubin. A bilirubin level above 342 μmol/L (20 mg/dL) was defined as severe hyperbilirubinemia and classified as pathological jaundice, and the modeled outcome was physiological jaundice. Resolution after postnatal day 14 was not a separate endpoint. Prolonged jaundice, including breast-milk jaundice, was entered as physiological when bilirubin stayed at or below 342 μmol/L and no etiology such as hemolysis or biliary disease had been identified [24]. Pathological jaundice was coded as a non-event. Infant eczema, recorded on the same questionnaire, was a history from birth to that visit. Preterm birth (12/1714), low birth weight (25/1740), and infant anemia (11/760) were collected but not modeled because events were few. Weight, length, and head circumference were measured at birth and at follow-up.

### 2.5. Covariates

Covariates were chosen a priori from clinical relevance and previous literature [25]: maternal age, prepregnancy body mass index (BMI), education, parity, gestational anemia, gestational hypertension, GDM, and geographic region. Regional economic level was omitted because it was collinear with region. BMI used reported prepregnancy weight and height and World Health Organization categories (<18.5, 18.5–25, 25–30, and ≥30 kg/m^2^). Education was primary school or below, junior high school, high school or vocational school, college or bachelor’s degree, or master’s degree or above. Anemia, hypertension, and GDM were binary hospital diagnoses and were not reclassified by laboratory thresholds. Region followed China’s grouping of East, North, Central, South, Northeast, and West China (Supplementary Table S1). Provincial GDP rankings for 2024 described the regions only and were not model covariates.

### 2.6. Statistical Analysis

Continuous variables are means with standard deviations, or medians with interquartile ranges, and categorical variables are counts and percentages. Latent class models used the 28 indicators among women with complete data (*N* = 1679). Categories were entered as 1–4, matching frequency scores of 0–3. Models were fit in R with poLCA [26] as intercept-only models, with one response probability per category. Given class, indicators were assumed independent, and no residual associations were added. Indicators for one supplement are dependent by construction, because one frequency was copied to each endorsed period. Each model from 2 to 10 classes used 30 random starts and up to 5000 EM iterations. The highest log-likelihood was kept when the EM algorithm finished within that limit. Fit was compared by the Akaike information criterion (AIC), Bayesian information criterion (BIC), sample-size-adjusted BIC, relative entropy, average posterior probability (AvePP), odds of correct classification, and minimum class size. Among models with entropy ≥ 0.70, minimum class size ≥ 5%, and minimum AvePP ≥ 0.70, the lowest BIC was selected. Women were assigned to the class with the highest posterior probability, and regressions used that class rather than posterior weights. In supplementary sensitivity analyses, one indicator per product, without a period split, was fit for the seven supplements plus zinc, selenium, and MMS (*N* = 1700). That model is not the primary set of 28 indicators with timing removed. The same 28 periods were also coded as used or not used, with frequency left out. The class model was refit among women who finished the questionnaire on or before delivery. In the same 1679 women, each of the seven supplements was entered as one frequency and was not split by period. Stabilized inverse-probability weights for 40–60-day follow-up were applied to jaundice and the three growth increments, without refitting the classes. The class model was refit after excluding 12 preterm births, defined as delivery before 37 completed weeks, with the same 28 indicators (*N* = 1667).

Multinomial logistic regression related covariates to class membership, with C6 (low-use) as the reference. Logistic models estimated cesarean delivery, macrosomia, physiological jaundice, and eczema. Linear models estimated birth size and the change in weight, length, and head circumference from birth to 40–60 days. Models adjusted for maternal age, prepregnancy BMI, education, parity, region, infant sex, gestational anemia, gestational hypertension, and GDM. Birth-size models also included gestational age, and growth models also included gestational age and the corresponding birth measurement. No clinical outcome was prespecified, and the pattern comparisons are exploratory. Benjamini–Hochberg false discovery rates were calculated within families. Each binary outcome had five pattern contrasts against C6. Birth length, birth weight, and head circumference contributed 15 contrasts, as did the three growth increments. These families were chosen because the exposure unit for inference was pattern membership relative to C6, not a single primary clinical endpoint. Correction was therefore applied within each pattern–outcome family rather than across all tests in the manuscript. Product-level analyses of any use versus never use, and of frequency strata, were prespecified as exploratory, are reported in the Supplementary Materials, and were undertaken outside of these families. Their q values do not change the pattern-level q values. Descriptive proportions use every recorded value. Adjusted models include a participant only when the outcome and every covariate are non-missing, so the denominator is smaller than the descriptive denominator and differs by outcome. Two-sided *p* values are reported, inference for these families uses the Benjamini–Hochberg q value, and analyses were performed in R version 4.4.3.

### 2.7. Ethical Approval and Informed Consent

The study was reviewed and approved by the Ethics Committee of Shanghai Jiao Tong University School of Medicine for Public Health and Nursing Research [ethics approval number: SJUPN-202304]. The study was registered with the Chinese Clinical Trial Registry (ChiCTR; registration number: ChiCTR2400092015). Written informed consent was obtained from all participants. All study procedures were conducted in accordance with the Declaration of Helsinki and applicable institutional and national ethical requirements.

## 3. Results

### 3.1. Characteristics of the Study Population

A total of 1758 mother–infant pairs comprised the analytic cohort (Figure 1). A total of 1679 participants (95.5%) had complete period-specific supplement-use indicators and were included in the cross-pregnancy LCA, and 1447 infants (82.3%) completed the 40–60-day postnatal follow-up. Baseline characteristics of the study population are shown in Table 1. Most women had normal prepregnancy BMI (66.0%) and a college or bachelor’s education (67.0%), and 53.4% were multiparous. GDM, gestational hypertension, and prenatal anemia were documented in 7.3%, 2.4%, and 6.8% of pregnancies, respectively. The cesarean delivery rate was 49.9% in this cohort. The mean (SD) gestational age at birth was 39.33 (1.16) weeks, and the mean birth weight was 3360.6 (432.1) g. Among infants with available follow-up data, neonatal physiological jaundice and eczema were reported in 27.4% and 40.6%, respectively.

The number of participants included in adjusted analyses varied according to outcome because analyses were restricted to complete cases. The analytical sample sizes were 1556 for cesarean delivery, 1540 for macrosomia, 1130 for neonatal physiological jaundice, and 1075 for infant eczema; 1127–1509 for birth anthropometric outcomes; and 965 for length gain, 1036 for weight gain, and 513 for head-circumference gain. Loss to follow-up was unevenly distributed: retention was highest in Northeast China (95.7%) and lowest in South China, where only one of 38 infants was retained, and follow-up differed further by maternal age, prepregnancy BMI, education, and folic acid use (Supplementary Table S2; Supplementary Figure S5).

### 3.2. Identification and Characterization of Longitudinal Prenatal Supplement-Use Patterns

Among the 1679 participants with complete period-specific supplement-use indicators, cross-pregnancy LCA was conducted and six distinct longitudinal patterns of prenatal supplement use were identified. The six-class model met all predefined fit and classification criteria and yielded the lowest BIC (59,273.9) among eligible solutions (Supplementary Table S3; Supplementary Figure S1). The seven-class solution also met the prespecified criteria but showed a slightly higher BIC (ΔBIC = 12.2). Solutions with eight or more classes generated at least one class below the prespecified minimum class size of 5%, and the nine-class solution failed to converge. The selected six-class model demonstrated good classification quality [27,28,29], with an entropy of 0.928, a minimum average posterior probability of 0.94, and a minimum odds of correct classification of 41.3 (Supplementary Table S4).

**Figure 2:**
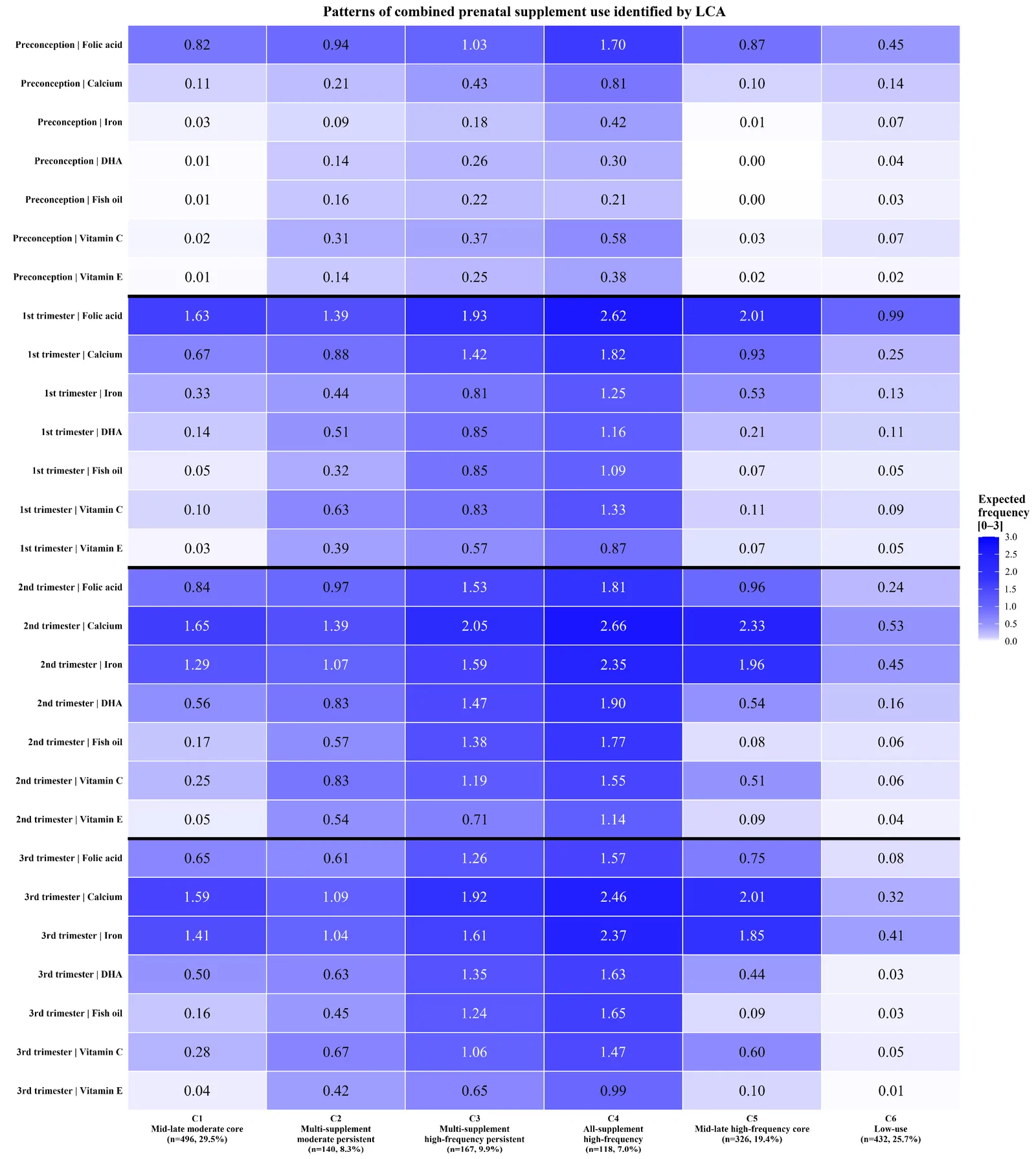
Six patterns of combined prenatal supplement use across preconception and pregnancy. Cross-pregnancy ordinal-frequency LCA (k = 6) among 1679 mothers with complete indicators. The heatmap shows the model-expected frequency for each period and supplement. A lower expected frequency in one period means that period was endorsed by fewer women in the class. The *x*-axis shows classes C1–C6 with pattern names and *n* (%). Expected frequency: 0 = none; 1 = 1–3 times/week; 2 = 4–7 times/week; 3 = ≥2 times/day.

The six patterns differed substantially in the timing, frequency, and breadth of supplement use across pregnancy. Class labels, sizes, and defining characteristics are presented in Table 2 and Figure 2. C6, the low-use pattern, comprised 432 participants (25.7%) and was used as the reference category in subsequent outcome analyses. C1 (mid-late moderate core use; *n* = 496, 29.5%) and C5 (mid-late high-frequency core use; *n* = 326, 19.4%) both endorsed folic acid, calcium, and iron, mainly in the second and third trimesters. They differ in the frequency category recorded once for those products: moderate in C1 and high in C5. C2 (multi-supplement moderate persistent-use; *n* = 140, 8.3%) and C3 (multi-supplement high-frequency persistent-use; *n* = 167, 9.9%) endorsed those core products plus DHA, fish oil, vitamin C, and vitamin E from the first trimester onward, and likewise differ only in that recorded frequency. C4 (all-supplement high-frequency; *n* = 118, 7.0%) combined all seven supplements at high recorded frequency. It is not high in every period.

The expected-frequency heatmaps further differentiated the six patterns (Figure 2). The mean expected frequency across all seven supplements was 0.1–0.2 in C6. In C1 and C5 this all-supplement mean was 0.7–0.9 in the second and third trimesters; that average does not separate C1 from C5, because four of the seven supplements were rarely endorsed in either class. Their separation is the recorded frequency of folic acid, calcium, and iron (Figure 2). In C2 and C3 the mean stayed elevated from the first trimester onward. C4 had the highest second-trimester mean (1.9). Its preconception mean was 0.6, the highest preconception value among the six classes, but much lower than 1.9 because preconception was endorsed less often than the second trimester. C2, C3, and C4 combined core micronutrients (folic acid, calcium, and iron) with DHA, fish oil, and antioxidant vitamins, whereas C1 and C5 were predominantly characterized by use of the core micronutrients during mid-to-late pregnancy (Figure 2; observed frequencies are shown in Supplementary Figure S2). Product-specific prevalence estimates and associations of individual supplements with study outcomes are presented in Supplementary Tables S21–S33.

Pattern membership varied according to geographic, sociodemographic, and pregnancy-related factors after multivariable adjustment (Supplementary Table S17). Relative to East China, women in North China had higher odds of membership in C5, C2, and C4 (adjusted OR 1.59, 95% CI 1.03–2.45; 1.96, 1.03–3.73; and 3.42, 1.55–7.55). Women from Northeast or South China had markedly higher odds of membership in C4 (5.40, 2.23–13.09 and 10.52, 1.96–56.37). In contrast, women from Central China were associated with lower odds of membership in C1 (0.55, 0.36–0.85) and C5 (0.47, 0.27–0.85), while those from West and Northeast China had lower odds of C1 membership (0.61, 0.39–0.94 and 0.52, 0.30–0.91).

Maternal age, education, and parity were also associated with pattern membership. Women aged 36 years or older were more likely to belong to the C4 pattern than younger women (adjusted ORs for 18–25, 26–30, and 31–35 versus ≥36 years: 0.29, 0.12–0.69; 0.43, 0.22–0.86; and 0.46, 0.24–0.90). Women with junior-high or high-school education had lower odds of C3 membership than women with a master’s or doctoral degree (0.26, 0.09–0.76; and 0.36, 0.14–0.88). Nulliparous women had higher odds of membership in C1 than multiparous women (1.52, 1.13–2.06).

Pregnancy complications were enriched in specific supplement-use patterns: women without gestational anemia had lower odds of C1 membership (0.47, 0.26–0.85; crude anemia prevalence 9.9% in C1 versus 4.2% in C6), and women without GDM had lower odds of C4 membership (0.35, 0.17–0.75; crude prevalence 11.9% in C4 versus 5.1% in C6). These gradients indicate that membership is jointly shaped by region, age, education, parity, and pregnancy complications rather than representing a pure exposure contrast.

### 3.3. Associations with Birth Outcomes

**Figure 3:**
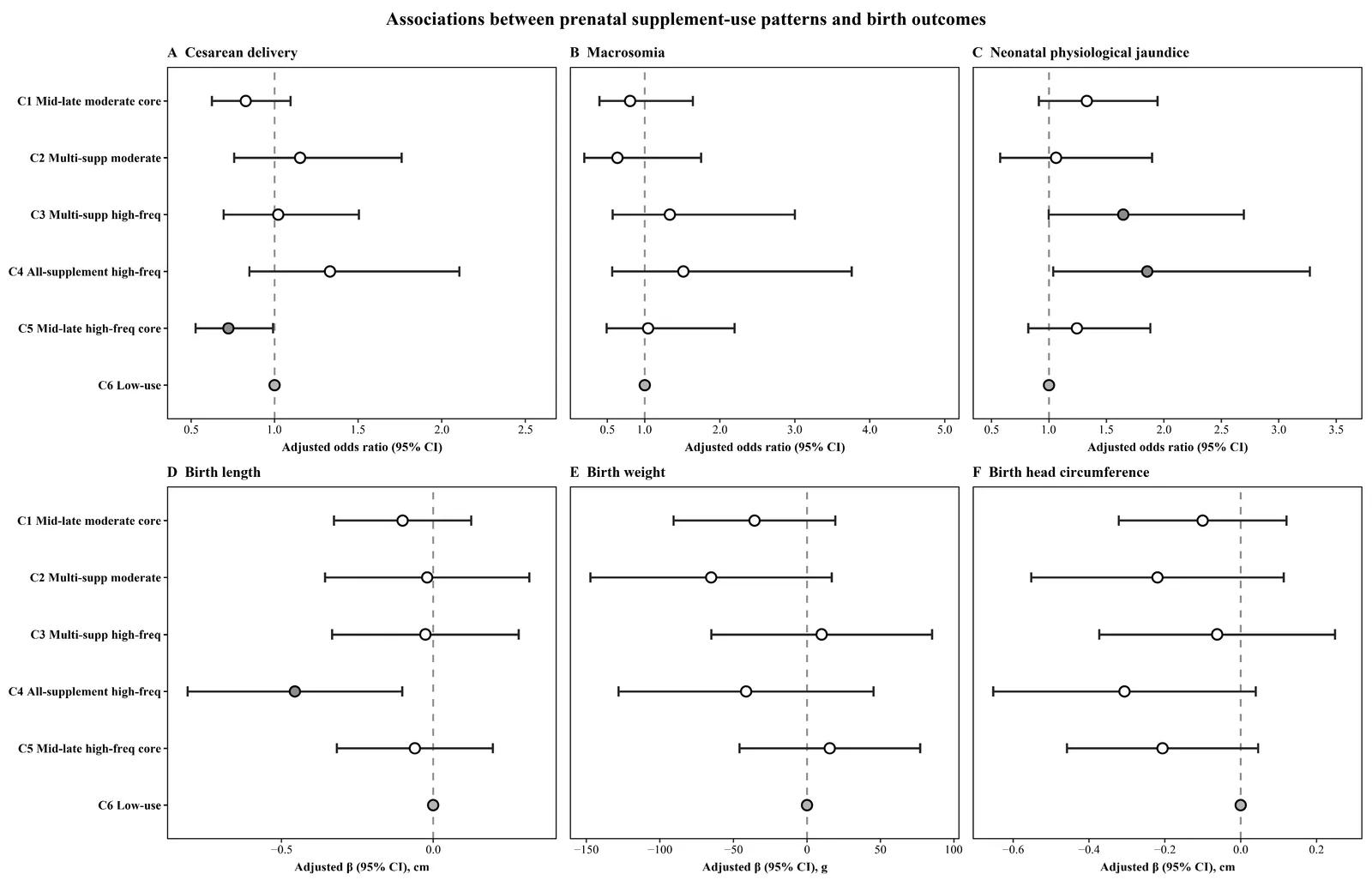
Birth outcomes according to prenatal supplement-use patterns. (**A**) Cesarean delivery. (**B**) Macrosomia (birth weight ≥ 4000 g). (**C**) Neonatal physiological jaundice. (**D**–**F**) Birth length, birth weight, and birth head circumference. C6 (low-use) was the reference pattern (OR = 1 or β = 0). Gray markers indicate nominal *p* < 0.05 only; open markers indicate neither. For panels (**A**–**C**), q is calculated within the five pattern comparisons of that outcome. For panels (**D**–**F**), q is calculated across the 15 comparisons of birth length, birth weight, and birth head circumference. Logistic regression models (**A**–**C**) were adjusted for maternal age, prepregnancy BMI, education, geographic region, parity, infant sex, gestational anemia, gestational hypertension, and GDM. Linear regression models (**D**–**F**) were additionally adjusted for gestational age at birth. Class labels are shown on the leftmost panel of each row.

Cesarean delivery occurred in 836 of 1678 mother–infant pairs in the LCA sample with delivery-mode data (49.8%; Supplementary Table S6). The crude rates were lowest in C1 and C5 (45.8% in C1 and 45.7% in C5) and highest in C4 (61.0%), compared with 51.7% in C6. In the adjusted analysis (*n* = 1556; 764 events), women in C5 (mid-late high-frequency core use) were associated with lower odds of cesarean delivery than those in C6 (adjusted OR 0.72, 95% CI 0.53–0.99; Figure 3A; Table 3). This association did not remain significant after within-family Benjamini–Hochberg correction (Supplementary Table S44). Compared separately with vaginal delivery, C5 was not associated with elective cesarean with indication (adjusted OR 0.77, 95% CI 0.54–1.11), elective cesarean without indication (0.58, 0.32–1.02), or intrapartum or emergency cesarean (0.89, 0.49–1.62). No statistically significant differences were observed for C1 (0.83, 0.62–1.10), C2 (1.15, 0.76–1.76), C3 (1.02, 0.69–1.50), or C4 (1.33, 0.85–2.11) compared with C6. At the individual-supplement level, any use of DHA (adjusted OR 1.24, 95% CI 1.00–1.53), fish oil (1.33, 1.05–1.67), vitamin E (1.34, 1.02–1.75), and selenium (1.59, 1.02–2.51) had a nominal association with higher odds of cesarean delivery (Supplementary Table S34).

Macrosomia was uncommon, occurring in 74 of 1540 infants included in the adjusted analysis (4.8%). No supplement-use pattern was significantly associated with macrosomia, although the point estimate for C4 was above the null (adjusted OR 1.51, 95% CI 0.57–3.76; Figure 3B). No individual supplement was associated with macrosomia in product-level analyses (Supplementary Table S35).

Neonatal physiological jaundice occurred in 298 of 1130 infants included in the adjusted analysis. Among infants with physiological jaundice, a link identifier in the analytic cohort, and complete onset and resolution times, prolonged jaundice (resolution after postnatal day 14) accounted for 154 of 226 (68.1%). Compared with C6, higher odds of neonatal physiological jaundice were observed among infants whose mothers belonged to C3 (adjusted OR 1.65, 95% CI 1.00–2.70) and C4 (1.86, 1.04–3.27) (Figure 3C; Table 3). These associations did not remain significant after within-family Benjamini–Hochberg correction (Supplementary Table S44). The corresponding crude prevalence was 32.5% in both C3 and C4, compared with 22.7% in C6 (Supplementary Table S6). No statistically significant differences were observed for C1 (1.33, 0.91–1.95), C2 (1.06, 0.58–1.90), or C5 (1.24, 0.82–1.88). At the individual-supplement level, any folic acid use was associated with higher odds of neonatal physiological jaundice (adjusted OR 1.70, 95% CI 1.05–2.87; Supplementary Table S36).

For birth anthropometry, C4 was associated with shorter birth length after adjustment (adjusted β −0.46 cm, 95% CI −0.81 to −0.10; *n* = 1445; Figure 3D, Table 3). No statistically significant differences between supplement-use patterns were observed for birth weight (*n* = 1509) or birth head circumference (*n* = 1127; Figure 3E,F). The birth-length association did not remain significant after within-family Benjamini–Hochberg correction (Supplementary Table S44). Preterm birth, low birth weight, and infant anemia were infrequent in the study population and were therefore not included in the adjusted association analyses.

In frequency-stratified individual-supplement analyses, birth weight and birth head circumference were not associated with any supplement use. Two nominal associations were observed for birth length: DHA use at a frequency of ≥2 times per day was associated with shorter birth length (adjusted β = −0.36 cm, *p* = 0.033), whereas vitamin E use at 1–3 times per week was associated with longer birth length (β = 0.33 cm, *p* = 0.046). Neither association remained statistically significant after false discovery rate (FDR) correction (Supplementary Tables S38, S40 and S42).

### 3.4. Associations with Early Infant Growth

**Figure 4:**
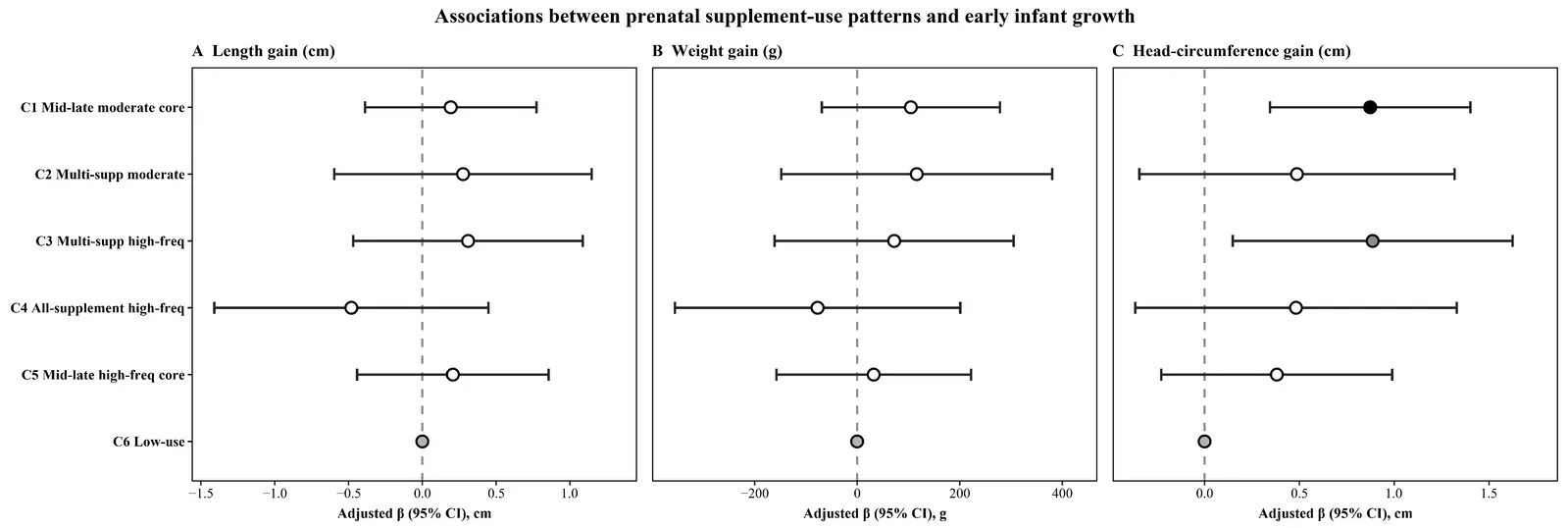
Early infant growth according to prenatal supplement-use patterns. (**A**) Length gain. (**B**) Weight gain. (**C**) Head-circumference gain, from birth to 40–60 days. C6 (low-use) is the reference (β = 0). Black markers indicate associations with Benjamini–Hochberg q < 0.05 across the 15 pattern–growth comparisons; gray markers indicate nominal *p* < 0.05 only; open markers indicate neither. Models were adjusted for the corresponding birth measure, gestational age, maternal age, prepregnancy BMI, education, geographic region, parity, infant sex, gestational anemia, gestational hypertension, and GDM. Class labels are shown on the leftmost panel.

Growth increments from birth to 40–60 days were largely similar across patterns after adjustment (Figure 4, Table 3). No statistically significant differences between patterns were observed for length gain (*n* = 965) or weight gain (*n* = 1036) (Figure 4A,B). The smaller head-circumference sample arose because mothers left that field blank more often than the fields for length, weight, jaundice, and eczema. No additional investigator exclusion was applied. Among 1171 infants with both length and weight at 40–60 days, head circumference was recorded for 729 (62.3%). The adjusted model included the 513 infants with a value at both birth and 40–60 days and with complete covariates. In this subsample, gain was greater in C1 (mid-late moderate core) than in C6 (adjusted β 0.87 cm, 95% CI 0.34–1.40; nominal *p* = 0.001). This was the only one of the 15 pattern–growth comparisons that remained statistically significant after Benjamini–Hochberg FDR correction (q = 0.019; Figure 4C). C3 showed a nominal association of similar magnitude (adjusted β 0.89 cm, 95% CI 0.15–1.62) that did not survive correction (q = 0.14). Associations between patterns and infant eczema were not statistically significant (430 events among 1075 infants in the adjusted analysis; adjusted ORs ranged from 0.58, 95% CI 0.33–1.01, for C2 to 1.38, 0.88–2.18, for C3; Table 3). At the product level, no supplement was associated with eczema (Supplementary Table S37), and no supplement frequency level was associated with length, weight, or head-circumference gain after FDR correction (Supplementary Tables S39, S41 and S43).

### 3.5. Sensitivity Analyses

Sensitivity analyses changed the indicators or the sample. None replaced the primary six-class solution. A ten-supplement model that ignored trimester (*N* = 1700) selected four classes, but both the products and the sample differed from the primary analysis (Supplementary Tables S18–S20; Supplementary Figures S3 and S4). On the same 28 indicators, omitting frequency selected eight classes, and only C6 stayed largely together (287/432, 66%; Supplementary Tables S46 and S47; Figure S6). Questionnaires completed on or before delivery, and one frequency for each of the seven products, likewise did not recover six classes (Supplementary Tables S48–S61). Greater head-circumference gain remained significant after Benjamini–Hochberg correction only in the before-delivery analysis (adjusted β 1.19 cm, 95% CI 0.41–1.97; *p* = 0.003; q = 0.012). Separately, the original six classes were retained and the outcome models were weighted for completion of the 40–60-day follow-up (Supplementary Tables S62–S66). In that analysis, the C1 head-circumference gain did not remain significant after correction (adjusted β 0.83 cm, 95% CI 0.27–1.40; *p* = 0.004; q = 0.056). Excluding 12 preterm births and refitting the same 28 indicators among the remaining 1667 women again selected six classes, and 1661 (99.6%) were assigned to the same class (Supplementary Table S67).

## 4. Discussion

### 4.1. Principal Findings

In this multicenter prospective cohort, prenatal supplement use was grouped into six classes defined by which periods were endorsed, the frequency category recorded once for each supplement, and which supplements were reported together [14,16,23,30,31]. The classes are not a ranking by dose. They separate different combinations of endorsed periods, that one recorded frequency, and product combination. In C6, few products and few periods were endorsed, and the recorded frequency was none or almost none. C1 and C5 both endorsed folic acid, calcium, and iron in the second and third trimesters, and differ in the recorded frequency category. C2 and C3 both endorsed those core products plus DHA, fish oil, vitamin C, and vitamin E from the first trimester onward, and differ in the recorded frequency category. C4 combined all seven supplements at a high recorded frequency. Endorsement was most consistent during pregnancy, and the class-conditional mean was highest in the second trimester (1.9). Preconception endorsement was more common than in the other classes, but the preconception mean was 0.6, because that period was endorsed less often. This is not a within-woman increase in frequency, and it is not high-frequency use in every period. These patterns were not random product combinations. Membership varied by region, parity, age, education, and selected pregnancy complications: C1 was relatively enriched for nulliparity and anemia, C3 for higher education, and C4 for GDM. This patterning supports viewing prenatal supplementation as joint, socially and clinically structured behavior across pregnancy, rather than as isolated single-product use [6,7,32].

Pattern–outcome comparisons were exploratory, and inference uses the within-family Benjamini–Hochberg q value. Relative to C6, C5 had a nominal association with lower odds of cesarean delivery (adjusted OR 0.72, 95% CI 0.53–0.99; q = 0.221). C3 and C4 had nominal associations with higher odds of neonatal physiological jaundice (1.65, 1.00–2.70, and 1.86, 1.04–3.27; q = 0.122 for both), and C4 with shorter birth length (β −0.46 cm, 95% CI −0.81 to −0.10; q = 0.175). None of these estimates remained significant after correction. No association was observed for macrosomia, birth weight, birth head circumference, infant eczema, or gains in length and weight. The only comparison that remained significant was greater head-circumference gain in C1 (β 0.87 cm, 95% CI 0.34–1.40; q = 0.019) among 513 infants.

The individual-supplement analyses provided complementary but not interchangeable information. Most outcomes were not consistently associated with any use versus non-use of individual supplements. Folic acid had a nominal association with higher odds of physiological jaundice, and DHA, fish oil, vitamin E, and selenium had nominal associations with higher odds of cesarean delivery (Supplementary Tables S34 and S36). These product-level comparisons were not part of the Benjamini–Hochberg families. Macrosomia, eczema, birth weight, birth head circumference, and 40–60-day length and weight gains likewise showed no product-level associations that survived FDR correction. These product-level signals did not map one-to-one onto the pattern results. Higher jaundice odds in C3 and C4 cannot be attributed simply to folic acid. The nominal C4 birth-length estimate had no corresponding product-level association that survived correction. Overall, the product-level results support interpreting supplementation as a cross-pregnancy joint regimen, rather than reducing pattern associations to the effect of any single product.

The observed associations should be interpreted as observational covariation rather than causal effects. Outcome models adjusted for maternal age, prepregnancy BMI, education, parity, geographic region, infant sex, and binary indicators of gestational anemia, gestational hypertension, and GDM. Residual confounding may remain through antenatal care, maternal health behaviors, socioeconomic circumstances beyond education and region, clinical indication, and obstetric management [33]. Pregnancy intention was not recorded. Preconception use is concentrated in C4, so that pattern cannot be separated from a planned pregnancy. In this cohort, C4 had a higher prevalence of GDM and C1 a higher prevalence of gestational anemia, so clinical conditions may have shaped both supplement use and the outcomes. These associations should not be read as evidence that changing a supplement regimen would change cesarean delivery, jaundice, birth length, or early head-circumference gain.

### 4.2. Comparison with Previous Studies

Compared with earlier Chinese surveys, the prevalence of folic acid, calcium, and iron use in this cohort was high and aligns with the continuing rise in prenatal supplement uptake reported in recent regional and national studies in China [6,7,32]. Folic acid coverage was broadly similar to the 81.7% reported in Sichuan during 2014–2016, whereas calcium (89.1%) and iron (76.4%) were markedly higher than in that survey (51.4% and 18.1%, respectively) and than the lower micronutrient use rates reported in a large Shaanxi population-based study [34]. These findings are broadly consistent with recent cross-sectional descriptions of substantial multi-product use and geographic and educational differences in supplementation [6,7,32]. High uptake in this cohort was not uniform, however. It was grouped into six classes that differ by endorsed periods, the recorded frequency, and product combination, and membership varied by region, education, parity, and selected pregnancy complications. In this cohort, GDM, gestational hypertension, and gestational anemia were recorded in 7.3%, 2.4%, and 6.8% of pregnancies, respectively (Table 1), which is slightly lower than in previous studies [35]. That patterning accords with prior evidence on who is more likely to adopt recommended micronutrients and who is more likely to add lipid or antioxidant products [6,7,32].

Methodologically, person-centered models of dietary patterns in pregnancy are well established and have been linked to GDM, hypertensive disorders of pregnancy, and birth outcomes [14,15,16,17,18,30,31]. For supplements, most Chinese outcome studies still analyze folic acid, iron, calcium, or formula powders as separate products rather than treating cross-pregnancy joint regimens as the exposure unit [19,34]. Our approach applied a person-centered framework to prenatal supplement-use regimens. The exposure was product combination, the periods a woman endorsed, and the one frequency she reported for each product. It does not contain a separate frequency for each trimester. This taxonomy provides exposure strata closer to real-world clinical behavior than surveys that report product prevalences alone. The resulting patterns therefore represent empirically derived exposure profiles rather than a simple ranking of supplement intensity.

For clinical endpoints, our findings partly converge with, and partly diverge from, prior reports. The largely null associations for length and weight gain to 40–60 days are consistent with long-term follow-up of antenatal multiple-micronutrient trials, which show at most modest early postnatal anthropometric benefits and no sustained growth advantage through childhood [8,9,36,37,38], and with meta-analytic evidence that prenatal DHA does not benefit offspring neurodevelopment [11]. The cesarean delivery rate was 49.9% (Table 1): 31.4% elective with an indication, 10.8% elective without an indication, and 7.7% intrapartum or emergency. This rate is higher than the national estimate of 34.9% for 2014 [39] and close to the hospital-based estimate of 43.5% for 2016–2020 [40]. The proportion of prolonged jaundice in our cohort is relatively high, but given that our cohort is a breastfeeding cohort, this value can also be explained; however, due to incomplete data, there remains some bias [24,41,42]. The C5 cesarean estimate did not survive correction (q = 0.221). C5 is defined by folic acid, calcium, and iron, and the nominal product-level estimates for DHA and fish oil were in the opposite direction. The nominal jaundice estimates in C3 and C4 (q = 0.122) and the nominal shorter birth length in C4 (q = 0.175) likewise provide no basis for a comparison with supplementation trials. Greater head-circumference gain in C1 was the only growth association to survive FDR correction (q = 0.019), but it was not reproduced for length or weight gain. This signal has no direct pattern-level precedent: trial syntheses report small early head-circumference benefits with multiple-micronutrient regimens in some settings, whereas longer-term trial follow-up has generally been null [37,38]. These differences should be interpreted in light of the substantially different exposure definitions and study designs: randomized trials evaluate specified supplementation interventions, whereas the present study grouped reported product combination, endorsed periods, and one recorded frequency.

### 4.3. Potential Explanations for the Observed Associations

The lower cesarean odds for C5 relative to C6 should be read against the relatively high cesarean delivery prevalence observed in this cohort [39,43]. Women in C5 may have been more closely engaged with antenatal care, differed in obstetric risk profiles, or received different patterns of prenatal counseling and clinical management. C5 records mid-to-late endorsement of folic acid, calcium, and iron at high recorded frequency. The C5 estimate remained in the same direction after adjustment for age, prepregnancy BMI, education, parity, region, infant sex, gestational anemia, gestational hypertension, and GDM, but it did not survive within-family correction (q = 0.221). Residual confounding of this nominal estimate can still occur through antenatal-care intensity, adherence to clinical recommendations, or obstetric decision-making. A Chinese multicenter study reported lower risks among women who continued folic acid into mid- and late pregnancy, and that exposure may partly reflect antenatal-care engagement or other health-related behavior [44]. C5 is not that exposure: it indicates that the mid-to-late periods were endorsed for folic acid, calcium, and iron at high recorded frequency. A lipid effect on labor is a less plausible reading of the nominal C5 estimate. C5 is defined by folic acid, calcium, and iron, and the nominal product-level estimates for DHA, fish oil, and vitamin E pointed toward higher odds of cesarean delivery. Parity was included, but previous cesarean delivery was not measured, so the C5 association cannot be separated from obstetric history. Compared separately from vaginal delivery, C5 was not associated with elective cesarean with an indication, elective cesarean without an indication, or intrapartum or emergency cesarean (Supplementary Table S45).

Signals in the intensive multi-supplement patterns should be read first as observational associations that may reflect both supplementation behavior and underlying clinical characteristics. C4 was relatively enriched for GDM, and maternal disease and delivery factors are repeatedly associated with neonatal hyperbilirubinemia [45,46]. Adjustment for gestational anemia, gestational hypertension, and GDM left the C3 and C4 jaundice estimates and the C4 birth-length estimate in the same direction. None survived within-family correction. These nominal estimates were therefore not fully explained by the measured binary indicators of pregnancy-related conditions. Residual confounding remains possible, because these variables may not capture disease severity, clinical management, antenatal-care intensity, or other indications for supplement use [33,47]. Jaundice in this analysis was a history of jaundice from birth to the 40–60-day follow-up. Physiological jaundice is common, usually transient, and shaped by gestational age, feeding, hemolysis risk, and monitoring intensity [45,48]. Accordingly, the observed associations should not be interpreted as evidence that intensive supplementation induces neonatal physiological jaundice. The modestly shorter birth length in C4 is likewise not sufficient to support an interpretation of growth restriction: trial syntheses indicate that multiple-micronutrient or lipid-based supplementation tends to increase, or at least not reduce, birth size [9,36,38], and individual-supplement analyses showed no corresponding consistent association with length. Folic acid had a nominal association with higher jaundice odds in this cohort, but that product-level signal is not sufficient to reduce the C3 and C4 pattern estimates to a folic acid effect.

Greater head-circumference gain in C1 was the only growth association that remained significant after multiplicity correction, but the head-circumference subsample was substantially smaller than those for length and weight, the estimate was not reproduced for length or weight gain, and C3 showed a nominal association of similar magnitude that did not survive FDR correction. Moderate core use in mid-to-late pregnancy could co-vary with early head-circumference change, and trial literature has reported small early head-circumference benefits with prenatal multiple-micronutrient supplementation in some settings [38]. In the present study, however, differential follow-up, differences in healthcare utilization or monitoring, and residual confounding cannot be excluded, particularly given the smaller sample available for head-circumference analyses. This finding is better treated as a signal requiring independent confirmation than as evidence of a developmental benefit.

### 4.4. Implications for Pattern-Informed Maternal Nutrition

Although these associations should not be read as nutrient mechanisms, the pattern perspective may provide a useful framework for considering heterogeneity in prenatal supplementation [2,49]. Supplement use in this cohort coalesced into a small number of recognizable longitudinal patterns, with membership associated with geographic, sociodemographic, and pregnancy-related characteristics. Membership varied by region, education, parity, and pregnancy complications (C1 was associated with higher prevalence of nulliparity and anemia, C3 with higher education, and C4 with GDM), consistent with previous evidence that prenatal supplement use and regimen selection are influenced by socioeconomic context and clinical indication [6,7,32]. These findings suggest that antenatal nutrition assessment may benefit from recording which products were used, which periods were endorsed, and the frequency written on the questionnaire, rather than recording products in isolation. The present data do not establish that class-based counseling would improve outcomes. These estimates compare the odds of outcomes across supplement-use pattern groups. An adjusted odds ratio does not give a woman’s own probability of cesarean delivery, jaundice, or a difference in growth. A statistically detectable association does not necessarily imply good predictive performance for identifying individuals who will experience an outcome [50].

The nominal estimates for cesarean delivery, jaundice, and birth length, and the head-circumference association that remained after correction, require independent confirmation. They do not support a change in clinical recommendations. Pattern-informed maternal nutrition strategies will require study designs that capture clinical indication, supplement dose and adherence, and relevant aspects of antenatal care more explicitly, and that can prospectively evaluate whether modification of supplement-use patterns is associated with improved maternal or infant outcomes. Such work could help determine whether the longitudinal patterns identified here are primarily markers of underlying maternal and healthcare characteristics or represent potentially modifiable aspects of prenatal nutrition behavior.

### 4.5. Strengths and Limitations

This study has several strengths. The multicenter prospective design across 16 provinces provided geographic and care-context heterogeneity. Indicators for preconception and each trimester record which periods were endorsed and carry the one frequency reported for each supplement. The six-class solution uses both of these features. With the same 28 period indicators and the same 1679 women retained, omitting the repeated frequency selects eight classes and does not recover these six (Supplementary Table S46; Figure S6). The exposure unit is this grouping of endorsed periods, one recorded frequency per product, and product combination, rather than single-product use.

Several limitations should be considered. First, supplement use was reported only once, at baseline, and was not updated thereafter (876 women completed the questionnaire on or before delivery, median 38.57 weeks; 809 completed it after delivery). Only reported frequency was available. Dose, formulation, amount, and adherence were unknown. Shared ingredients prevent adding frequencies across products to estimate nutrient intake. High-frequency and intensive denote the frequency category and the product count. Recall and reporting bias cannot be excluded [51]. The 809 women who reported after delivery knew the mode of delivery and birth size, and hospital records do not remove that influence. Among women who completed the questionnaire on or before delivery, the six-class solution and the nominal associations with cesarean delivery, jaundice, and birth length were not reproduced. Second, one frequency covered every endorsed period, so mid-to-late frequency denotes those periods at a single value, not within-person change. Omitting frequency selected eight classes rather than six (Supplementary Table S46; Figure S6). One frequency for each of the same seven products selected five classes. The primary taxonomy therefore depends on both the endorsed periods and the repeated frequency, and should not be read as a stable grouping of frequency alone. Outcome models used the modal class, so the confidence intervals omit uncertainty in class assignment (minimum average posterior probability 0.94; minimum odds of correct classification 41.3; Supplementary Table S4). Third, incomplete timing data excluded zinc, selenium, and multiple-micronutrient supplements from the primary LCA. Fourth, jaundice, eczema, and 40–60-day length and weight gain were available-case analyses, without imputation. Follow-up was associated with region, maternal age, prepregnancy BMI, education, and folic acid use. Of 38 infants enrolled in South China, 37 were not retained, so South China is not represented, and the direction and magnitude of the bias are unknown. Head-circumference gain required values at birth and at 40–60 days. Mothers left this field blank more often than length, weight, jaundice, and eczema. The estimate applies to infants for whom the field was completed and is additionally susceptible to selection bias. Fifth, adjustment for gestational anemia, gestational hypertension, and GDM did not separate indication for use or care intensity from exposure [33]. Diagnoses were not necessarily uniform across the 22 hospitals. Prepregnancy BMI was categorized using WHO cut points (<18.5, 18.5–25, 25–30, and ≥30 kg/m^2^) for comparability with the international literature. Chinese adult cut points (24.0 and 28.0 kg/m^2^) are widely used in domestic studies and would reclassify some women into higher categories. The WHO categories were applied throughout the analyses. The magnitude of this residual bias was not estimated. Of 1714 infants with a recorded gestational age, only 12 were preterm. Follow-up ended at 40–60 days, short of later growth and neurodevelopment, endpoints that have often been null in longer-term trials [11,37].

Enrollment covered 22 maternal and child health hospitals in 16 provinces. The criteria (term birth, age 20–40 years, intention and ability to breastfeed, access to a smartphone, and postnatal care at the delivery hospital, with exclusion of conditions that affected breastfeeding) were not reapplied after enrollment. No sample-size calculation was performed for the pattern–outcome comparisons, which had no prespecified outcome; the planned sample of 3185 was calculated for the mean total nitrogen concentration of mature milk. Cesarean associations apply where elective cesarean is common and close to recent hospital-based estimates. They do not extend to settings with a substantially lower cesarean rate. Causal interpretation is precluded. Future work needs to assess dose, adherence, indication, antenatal care, and longer-term follow-up.

## 5. Conclusions

In this Chinese multicenter prospective mother–infant cohort, latent class analysis identified six patterns of supplement use based on endorsed periods, reported frequency categories, and supplement combinations from preconception through pregnancy. The pattern–outcome comparisons were exploratory. After within-family Benjamini–Hochberg correction, the associations with cesarean delivery, physiological jaundice, and birth length did not remain significant. Greater head-circumference gain in the mid-to-late moderate core pattern remained significant after correction among 513 infants in the adjusted analysis. Macrosomia, birth weight, birth head circumference, gains in length and weight, and infant eczema showed no statistically significant associations with pattern membership. Product-level analyses were exploratory. Nominal associations for individual supplements did not reduce the pattern results to one product. These estimates are observational and do not show that changing a supplement regimen would change these endpoints. The six patterns include one frequency assigned to every endorsed period and were not recovered when frequency was omitted, so the estimates apply to this classification. Clinical recommendations should not be inferred from these results. Further studies with period-specific exposure assessment, detailed information on supplement dose and adherence, and more complete follow-up are needed to determine whether these patterns have reproducible clinical relevance.

## Figures and Tables

**Table 1:** Comprehensive baseline characteristics of mother–infant pairs.

Section	Characteristic	Value
Maternal characteristics	Maternal age (years)	
	18–25	189 (10.8%)
	26–30	753 (42.8%)
	31–35	616 (35.0%)
	≥36	200 (11.4%)
	Prepregnancy BMI	
	Underweight (<18.5)	132 (7.5%)
	Normal weight (18.5–25)	1160 (66.0%)
	Overweight (25–30)	264 (15.0%)
	Obese (≥30)	72 (4.1%)
	Missing	130 (7.4%)
	Education level	
	Primary school or below	14 (0.8%)
	Junior high school	167 (9.5%)
	High school/vocational school	277 (15.8%)
	College or bachelor’s degree	1178 (67.0%)
	Master’s degree or above	119 (6.8%)
	Missing	3 (0.2%)
	Parity	
	Primiparous	816 (46.4%)
	Multiparous	939 (53.4%)
	Missing	3 (0.2%)
	GDM	
	Yes	129 (7.3%)
	No	1629 (92.7%)
	Gestational hypertension	
	Yes	42 (2.4%)
	No	1716 (97.6%)
	Gestational anemia	
	Yes	119 (6.8%)
	No	1639 (93.2%)
	Geographic region	
	East China	371 (21.1%)
	North China	571 (32.5%)
	Central China	253 (14.4%)
	South China	38 (2.2%)
	Northeast China	209 (11.9%)
	West China	316 (18.0%)
Neonatal characteristics	Sex (*n* = 1758)	
	Boys	882 (50.2%)
	Girls	876 (49.8%)
	Gestational age (weeks)	39.33 ± 1.16
	Birth weight (g)	3360.6 ± 432.1
	Mode of delivery (*n* = 1757)	
	Spontaneous vaginal delivery	832 (47.4%)
	Assisted vaginal delivery	48 (2.7%)
	Elective cesarean section with indication	552 (31.4%)
	Elective cesarean section without indication	190 (10.8%)
	Intrapartum/emergency cesarean section	135 (7.7%)
Postnatal follow-up (40–60 days)	Physiological jaundice (*n* = 1238)	
	No	899 (72.6%)
	Yes	339 (27.4%)
	Eczema (*n* = 1206)	
	No	716 (59.4%)
	Yes	490 (40.6%)

Data are presented as *n* (%) unless otherwise indicated; gestational age and birth weight are shown as mean ± SD. Percentages are based on the full cohort (*N* = 1758) unless a different denominator is specified. Data on mode of delivery were missing for 1 participant. Physiological jaundice and eczema were recorded at the 40–60-day postpartum follow-up as histories from birth to that visit among infants with available records (*n* = 1238 and *n* = 1206, respectively, of 1447 infants completing follow-up). Prepregnancy BMI categories were defined according to WHO criteria.

**Table 2:** Prenatal supplement-use patterns from LCA: class labels, definitions, and sizes (*N* = 1679).

Class	Label	Definition	*n*	Percent
C1	Mid-late moderate core	Core supplements (folic acid, calcium, iron) endorsed mainly in the second and third trimesters, at a moderate recorded frequency. That frequency is copied to each endorsed period.	496	29.5
C2	Multi-supplement moderate persistent	Core supplements plus vitamin C, DHA, fish oil, and vitamin E, endorsed from the first trimester onward, at a moderate recorded frequency.	140	8.3
C3	Multi-supplement high-frequency persistent	The same products and the same periods as C2, at a high recorded frequency.	167	9.9
C4	All-supplement high-frequency	All seven supplements at a high recorded frequency. Pregnancy periods were endorsed most consistently.	118	7.0
C5	Mid-late high-frequency core	The same core supplements and the same periods as C1, at a high recorded frequency.	326	19.4
C6	Low-use	Consistently low use of all supplements across all periods.	432	25.7

Data are *n* (%) of the 1679 participants included in the LCA. C6 (low-use) was the reference category in outcome models. Class labels are shorthand. ‘High-frequency’ refers to the frequency category reported for a given supplement and assigned to each pregnancy period in which its use was endorsed. A lower expected frequency in one period means fewer women endorsed that period, not a lower frequency within the same woman. Percentages may not sum to 100 because of rounding.

**Table 3:** Adjusted associations of prenatal supplement-use patterns with birth outcomes and early infant growth.

Outcome	Pattern Class	Adjusted Estimate (95% CI)	*p* Value	FDR q
Birth Outcomes				
Cesarean delivery (OR), 764/1556	C1 Mid-late moderate core	0.83 (0.62–1.10)	0.186	0.359
	C2 Multi-supplement moderate persistent	1.15 (0.76–1.76)	0.509	0.636
	C3 Multi-supplement high-frequency persistent	1.02 (0.69–1.50)	0.913	0.913
	C4 All-supplement high-frequency	1.33 (0.85–2.11)	0.216	0.359
	C5 Mid-late high-frequency core	0.72 (0.53–0.99)	0.044	0.221
	C6 Low-use	1.00 (Reference)	—	—
Neonatal physiological jaundice (OR), 298/1130	C1 Mid-late moderate core	1.33 (0.91–1.95)	0.140	0.234
	C2 Multi-supplement moderate persistent	1.06 (0.58–1.90)	0.843	0.843
	C3 Multi-supplement high-frequency persistent	1.65 (1.00–2.70)	0.049	0.122
	C4 All-supplement high-frequency	1.86 (1.04–3.27)	0.034	0.122
	C5 Mid-late high-frequency core	1.24 (0.82–1.88)	0.304	0.380
	C6 Low-use	1.00 (Reference)	—	—
Infant eczema (OR), 430/1075	C1 Mid-late moderate core	1.09 (0.78–1.54)	0.615	0.615
	C2 Multi-supplement moderate persistent	0.58 (0.33–1.01)	0.060	0.300
	C3 Multi-supplement high-frequency persistent	1.38 (0.88–2.18)	0.165	0.412
	C4 All-supplement high-frequency	1.29 (0.75–2.21)	0.360	0.471
	C5 Mid-late high-frequency core	0.84 (0.58–1.23)	0.377	0.471
	C6 Low-use	1.00 (Reference)	—	—
Birth length, cm (β), *N* = 1445	C1 Mid-late moderate core	−0.10 (−0.33 to 0.13)	0.382	0.637
	C2 Multi-supplement moderate persistent	−0.02 (−0.36 to 0.32)	0.908	0.908
	C3 Multi-supplement high-frequency persistent	−0.03 (−0.33 to 0.28)	0.870	0.908
	C4 All-supplement high-frequency	−0.46 (−0.81 to −0.10)	0.012	0.175
	C5 Mid-late high-frequency core	−0.06 (−0.32 to 0.20)	0.646	0.869
	C6 Low-use	0 (Reference)	—	—
Birth weight, g (β), *N* = 1509	C1 Mid-late moderate core	−35.64 (−90.66 to 19.38)	0.204	0.510
	C2 Multi-supplement moderate persistent	−65.14 (−147.21 to 16.93)	0.120	0.449
	C3 Multi-supplement high-frequency persistent	10.07 (−65.01 to 85.14)	0.793	0.908
	C4 All-supplement high-frequency	−41.36 (−128.09 to 45.37)	0.350	0.637
	C5 Mid-late high-frequency core	15.57 (−45.88 to 77.03)	0.619	0.869
	C6 Low-use	0 (Reference)	—	—
Birth head circumference, cm (β), *N* = 1127	C1 Mid-late moderate core	−0.10 (−0.32 to 0.12)	0.374	0.637
	C2 Multi-supplement moderate persistent	−0.22 (−0.55 to 0.11)	0.196	0.510
	C3 Multi-supplement high-frequency persistent	−0.06 (−0.37 to 0.25)	0.695	0.869
	C4 All-supplement high-frequency	−0.31 (−0.65 to 0.04)	0.083	0.449
	C5 Mid-late high-frequency core	−0.21 (−0.46 to 0.05)	0.109	0.449
	C6 Low-use	0 (Reference)	—	—
Early Infant Growth				
Head-circumference gain, birth to 40–60 d, cm (β), *N* = 513	C1 Mid-late moderate core	0.87 (0.34 to 1.40)	0.001	0.019
	C2 Multi-supplement moderate persistent	0.49 (−0.34 to 1.32)	0.250	0.628
	C3 Multi-supplement high-frequency persistent	0.89 (0.15 to 1.62)	0.019	0.140
	C4 All-supplement high-frequency	0.48 (−0.37 to 1.33)	0.264	0.628
	C5 Mid-late high-frequency core	0.38 (−0.23 to 0.99)	0.220	0.628
	C6 Low-use	0 (Reference)	—	—
Length gain, birth to 40–60 d, cm (β), *N* = 965	C1 Mid-late moderate core	0.19 (−0.39 to 0.77)	0.516	0.628
	C2 Multi-supplement moderate persistent	0.28 (−0.60 to 1.15)	0.535	0.628
	C3 Multi-supplement high-frequency persistent	0.31 (−0.47 to 1.09)	0.435	0.628
	C4 All-supplement high-frequency	−0.48 (−1.41 to 0.45)	0.310	0.628
	C5 Mid-late high-frequency core	0.21 (−0.44 to 0.85)	0.532	0.628
	C6 Low-use	0 (Reference)	—	—
Weight gain, birth to 40–60 d, g (β), *N* = 1036	C1 Mid-late moderate core	104.74 (−69.10 to 278.57)	0.237	0.628
	C2 Multi-supplement moderate persistent	116.18 (−147.94 to 380.31)	0.388	0.628
	C3 Multi-supplement high-frequency persistent	72.10 (−160.94 to 305.15)	0.544	0.628
	C4 All-supplement high-frequency	−77.17 (−355.54 to 201.20)	0.587	0.628
	C5 Mid-late high-frequency core	32.31 (−157.25 to 221.88)	0.738	0.738
	C6 Low-use	0 (Reference)	—	—

Estimates are adjusted odds ratios (ORs) for binary outcomes and adjusted β coefficients for birth anthropometry and growth increments, with C6 (low-use) as the reference class; models were adjusted as described in the Methods. For binary outcomes, values after the outcome label are events/analytic *N*; for continuous outcomes, *N* is the analytic sample. Analyses used complete cases, so *N* varies by endpoint. q values are Benjamini–Hochberg-adjusted within families: five pattern comparisons for each binary outcome; 15 comparisons across birth length, birth weight, and birth head circumference; and 15 comparisons across the three growth increments. CI, confidence interval. —, reference class.

## Data Availability

The datasets generated and/or analyzed during the current study are available from the corresponding author on reasonable request due to privacy and ethical restrictions.

## Supplementary Materials

The following supporting information can be downloaded at https://www.mdpi.com/article/10.3390/nu18213649/s1, Figure S1. Model comparison for the cross-pregnancy latent class analysis. Bayesian information criterion (BIC) and sample-size-adjusted BIC (aBIC) are shown for models with 2–10 classes fitted to 28 period-by-supplement frequency indicators (*N* = 1679). The dashed line marks the selected six-class model, which had the lowest BIC among models with relative entropy ≥ 0.70, a minimum class size ≥ 5%, and a minimum average posterior probability ≥ 0.70; Figure S2. Observed mean supplement-use frequency by latent class. Each cell is the mean frequency score among women assigned to that class. Rows are pregnancy period and supplement; columns are the six classes, labelled with the pattern name and the number and percentage of women. Scores are 0 = none, 1 = 1–3 times/week, 2 = 4–7 times/week, and 3 = ≥2 times/day; Figure S3. Sensitivity latent class analysis using cross-sectional binary indicators. Folic acid, calcium, iron, DHA, fish oil, vitamin C, vitamin E, zinc, selenium, and multiple micronutrient supplements were each coded as any use versus none, without separation by pregnancy period (*N* = 1700). A four-class model was selected. Cell values are model-estimated probabilities of use; Figure S4. Sensitivity latent class analysis using cross-sectional frequency indicators. Folic acid, calcium, iron, DHA, fish oil, vitamin C, vitamin E, zinc, selenium, and multiple micronutrient supplements were each entered as a four-level variable, without separation by pregnancy period (*N* = 1700): 0 = none, 1 = 1–3 times/week, 2 = 4–7 times/week, and 3 = ≥2 times/day. A four-class model was selected. Cell values are model-expected frequency scores; Figure S5. Odds of loss to follow-up at 40–60 days after birth. Points are univariable odds ratios and horizontal bars are 95% confidence intervals, on a logarithmic scale, comparing women lost to follow-up with those who completed the assessment. The vertical line is an odds ratio of 1. Reference categories are North China, maternal age 26–30 years, normal prepregnancy body-mass index, a college or bachelor’s degree, and any use of folic acid; Figure S6. Sensitivity latent class analysis using period-specific binary indicators. Folic acid, calcium, iron, DHA, fish oil, vitamin C, and vitamin E were each coded as used or not used in preconception and in the first, second, and third trimesters (28 indicators). Frequency did not enter the class model. Cells are the model-estimated probability of use in that period. Columns are the eight-class solution selected by the same rules as the primary analysis (entropy ≥ 0.70, minimum class size ≥ 5%, minimum average posterior probability ≥ 0.70, then lowest BIC; *N* = 1679; entropy 0.894). Labels B1–B8 refer only to this sensitivity solution and are not classes C1–C6. The number and percentage of women are shown under each column. Table S1. Classification of Provinces by Geographic Region in China. Table S2. Baseline Characteristics of Participants Completing Versus Lost to 40–60-Day Follow-up. Table S3. Model Fit Indices for Cross-Pregnancy Latent Class Analysis of Maternal Supplement-Use Patterns. Table S4. Classification Diagnostics for the Selected Six-Class Model of Maternal Supplement-Use Patterns. Table S5. Baseline Characteristics by Maternal Supplement-Use Pattern. Table S6. Outcome Event Counts and Rates by Maternal Supplement-Use Pattern. Table S7. Association Between Maternal Supplement-Use Patterns and Cesarean Delivery. Table S8. Association Between Maternal Supplement-Use Patterns and Macrosomia. Table S9. Association Between Maternal Supplement-Use Patterns and Neonatal Physiological Jaundice. Table S10. Association Between Maternal Supplement-Use Patterns and Infant Eczema. Table S11. Association Between Maternal Supplement-Use Patterns and Length at Birth. Table S12. Association Between Maternal Supplement-Use Patterns and Weight at Birth. Table S13. Association Between Maternal Supplement-Use Patterns and Head Circumference at Birth. Table S14. Association Between Maternal Supplement-Use Patterns and Length Gain (40–60 d). Table S15. Association Between Maternal Supplement-Use Patterns and Weight Gain (40–60 d). Table S16. Association Between Maternal Supplement-Use Patterns and Head Circumference Gain (40–60 d). Table S17. Multivariable Multinomial Logistic Regression for Maternal Supplement-Use Pattern Membership. Table S18. Model Fit Indices for Sensitivity Latent Class Analyses of Maternal Supplement Use. Table S19. Cross-Classification of Primary and Binary Sensitivity Latent Classes of Maternal Supplement Use. Table S20. Cross-Classification of Primary and Ordinal-Frequency Sensitivity Latent Classes of Maternal Supplement Use. Table S21. Univariate Analysis of Calcium Supplement Use. Table S22. Univariate Analysis of Iron Supplement Use. Table S23. Univariate Analysis of Zinc Supplement Use. Table S24. Univariate Analysis of Fish Oil Supplement Use. Table S25. Univariate Analysis of Vitamin E Supplement Use. Table S26. Univariate Analysis of Vitamin C Supplement Use. Table S27. Univariate Analysis of Folic Acid Supplement Use. Table S28. Univariate Analysis of MMS Supplement Use. Table S29. Univariate Analysis of DHA Supplement Use. Table S30. Univariate Analysis of Selenium Supplement Use. Table S31. Multivariable Logistic Regression for Calcium, Iron, and Zinc Supplement Use. Table S32. Multivariable Logistic Regression for Fish Oil, Vitamin E, and Vitamin C Supplement Use. Table S33. Multivariable Logistic Regression for Folic Acid, MMS, DHA, and Selenium Supplement Use. Table S34. Association Between Maternal Supplement Use and Cesarean Delivery. Table S35. Association Between Maternal Supplement Use and Macrosomia. Table S36. Association Between Maternal Supplement Use and Neonatal Physiological Jaundice. Table S37. Association Between Maternal Supplement Use and Infant Eczema. Table S38. Association Between Supplement Use and Length at Birth. Table S39. Association Between Supplement Use and Length Gain (40–60d). Table S40. Association Between Supplement Use and Weight at Birth. Table S41. Association Between Supplement Use and Weight Gain (40–60d). Table S42. Association Between Supplement Use and Head Circumference at Birth. Table S43. Association Between Supplement Use and Head Circumference Gain (40–60d). Table S44. Benjamini–Hochberg False Discovery Rates for Pattern–Outcome Families. Table S45. Associations Between Maternal Supplement-Use Patterns and Cesarean Subtypes. Table S46. Model Fit Indices for the Period-Specific Binary Sensitivity Latent Class Analysis. Table S47. Cross-Classification of the Primary Six-Class Solution and the Period-Specific Binary Eight-Class Solution. Table S48. Model Fit Indices for the Latent Class Analysis Restricted to Questionnaires Completed on or before Delivery. Table S49. Classification Diagnostics for the Selected Five-Class Before-Delivery Model. Table S50. Association Between Before-Delivery Supplement-Use Classes and Cesarean Delivery. Table S51. Association Between Before-Delivery Supplement-Use Classes and Neonatal Physiological Jaundice. Table S52. Association Between Before-Delivery Supplement-Use Classes and Length at Birth. Table S53. Association Between Before-Delivery Supplement-Use Classes and Head Circumference Gain (40–60 d). Table S54. Model Fit Indices for the One-Indicator-per-Product Sensitivity Latent Class Analysis. Table S55. Classification Diagnostics for the Selected Five-Class One-Indicator Model. Table S56. Association Between One-Indicator Supplement-Use Classes and Cesarean Delivery. Table S57. Association Between One-Indicator Supplement-Use Classes and Neonatal Physiological Jaundice. Table S58. Association Between One-Indicator Supplement-Use Classes and Length at Birth. Table S59. Association Between One-Indicator Supplement-Use Classes and Head Circumference Gain (40–60 d). Table S60. Association Between One-Indicator Supplement-Use Classes and Length Gain (40–60 d). Table S61. Association Between One-Indicator Supplement-Use Classes and Weight Gain (40–60 d). Table S62. Logistic Model for Completion of the 40–60-Day Follow-up Used to Derive Inverse-Probability Weights. Table S63. Follow-up-Weighted Association Between Maternal Supplement-Use Patterns and Neonatal Physiological Jaundice. Table S64. Follow-up-Weighted Association Between Maternal Supplement-Use Patterns and Length Gain (40–60 d). Table S65. Follow-up-Weighted Association Between Maternal Supplement-Use Patterns and Weight Gain (40–60 d). Table S66. Follow-up-Weighted Association Between Maternal Supplement-Use Patterns and Head Circumference Gain (40–60 d). Table S67. Model Fit Indices for the Latent Class Analysis Excluding Preterm Births.

## Abbreviations

The following abbreviations are used in this manuscript:

LCA	Latent class analysis
DHA	Docosahexaenoic acid
BMI	Body mass index
BIC	Bayesian information criterion
CI	Confidence interval
FDR	False discovery rate
GDM	Gestational diabetes mellitus
OR	Odds ratio
SD	Standard deviation
