# Supplementary material for: Patterns of Combined Prenatal Supplement Use and Associations with Birth Outcomes and Early Infant Growth: A Multicenter Prospective Mother–Infant Cohort Study in China
Source: Nutrients. 2026 Nov 2;18(21):3649. doi: 10.3390/nu18213649 (PMC13765063; doi:10.3390/nu18213649)
Supplement: Supplementary file 1 [file nutrients-18-03649-s001.zip › nutrients-4626773-supplementary.pdf]

Supplementary Tables

Table of contents

Table S1. Classification of Provinces by Geographic Region in China 5

Table S2. Baseline Characteristics of Participants Completing Versus Lost to 40–60-Day Follow-up 6

Table S3. Model Fit Indices for Cross-Pregnancy Latent Class Analysis of Maternal Supplement-Use Patterns 8

Table S4. Classification Diagnostics for the Selected Six-Class Model of Maternal Supplement-Use Patterns 9

Table S5. Baseline Characteristics by Maternal Supplement-Use Pattern 10

Table S6. Outcome Event Counts and Rates by Maternal Supplement-Use Pattern 11

Table S7. Association Between Maternal Supplement-Use Patterns and Cesarean Delivery 12

Table S8. Association Between Maternal Supplement-Use Patterns and Macrosomia 13

Table S9. Association Between Maternal Supplement-Use Patterns and Neonatal Physiological Jaundice 14

Table S10. Association Between Maternal Supplement-Use Patterns and Infant Eczema 15

Table S11. Association Between Maternal Supplement-Use Patterns and Length at Birth 16

Table S12. Association Between Maternal Supplement-Use Patterns and Weight at Birth 17

Table S13. Association Between Maternal Supplement-Use Patterns and Head Circumference at Birth 18

Table S14. Association Between Maternal Supplement-Use Patterns and Length Gain (40-60d) 19

Table S15. Association Between Maternal Supplement-Use Patterns and Weight Gain (40-60d) 20

Table S16. Association Between Maternal Supplement-Use Patterns and Head Circumference Gain (40-60d) 21

Table S17. Multivariable Multinomial Logistic Regression for Maternal Supplement-Use Pattern Membership 22

Table S18. Model Fit Indices for Sensitivity Latent Class Analyses of Maternal Supplement Use 23

Table S19. Cross-Classification of Primary and Binary Sensitivity Latent Classes of Maternal Supplement Use 24

Table S20. Cross-Classification of Primary and Ordinal-Frequency Sensitivity Latent Classes of Maternal Supplement Use 25

Table S21. Univariate Analysis of Calcium Supplement Use 26

Table S22. Univariate Analysis of Iron Supplement Use 27

Table S23. Univariate Analysis of Zinc Supplement Use 28

Table S24. Univariate Analysis of Fish Oil Supplement Use 29

Table S25. Univariate Analysis of Vitamin E Supplement Use 30

Table S26. Univariate Analysis of Vitamin C Supplement Use 31

Table S27. Univariate Analysis of Folic Acid Supplement Use 32

Table S28. Univariate Analysis of MMS Supplement Use 33

Table S29. Univariate Analysis of DHA Supplement Use 34

Table S30. Univariate Analysis of Selenium Supplement Use 35

Table S31. Multivariable Logistic Regression for Calcium, Iron, and Zinc Supplement Use 36

Table S32. Multivariable Logistic Regression for Fish Oil, Vitamin E, and Vitamin C Supplement Use 37

Table S33. Multivariable Logistic Regression for Folic Acid, MMS, DHA, and Selenium Supplement Use 38

Table S34. Association Between Maternal Supplement Use and Cesarean Delivery 39

Table S35. Association Between Maternal Supplement Use and Macrosomia 40

Table S36. Association Between Maternal Supplement Use and Neonatal Physiological Jaundice 41

Table S37. Association Between Maternal Supplement Use and Infant Eczema 42

Table S38. Association Between Supplement Use and Length at Birth 43

Table S39. Association Between Supplement Use and Length Gain (40-60d) 44

Table S40. Association Between Supplement Use and Weight at Birth 45

Table S41. Association Between Supplement Use and Weight Gain (40-60d) 46

Table S42. Association Between Supplement Use and Head Circumference at Birth 47

Table S43. Association Between Supplement Use and Head Circumference Gain (40-60d) 48

Table S44. Benjamini–Hochberg False Discovery Rates for Pattern–Outcome Families 49

Table S45. Associations Between Maternal Supplement-Use Patterns and Cesarean Subtypes 51

Table S46. Model Fit Indices for the Period-Specific Binary Sensitivity Latent Class Analysis 52

Table S47. Cross-Classification of the Primary Six-Class Solution and the Period-Specific Binary Eight-Class Solution 53

Table S48. Model Fit Indices for the Latent Class Analysis Restricted to Questionnaires Completed on or before Delivery 54

Table S49. Classification Diagnostics for the Selected Five-Class Before-Delivery Model 55

Table S50. Association Between Before-Delivery Supplement-Use Classes and Cesarean Delivery 56

Table S51. Association Between Before-Delivery Supplement-Use Classes and Neonatal Physiological Jaundice 57

Table S52. Association Between Before-Delivery Supplement-Use Classes and Length at Birth 58

Table S53. Association Between Before-Delivery Supplement-Use Classes and Head Circumference Gain (40–60 d) 59

Table S54. Model Fit Indices for the One-Indicator-per-Product Sensitivity Latent Class Analysis 60

Table S55. Classification Diagnostics for the Selected Five-Class One-Indicator Model 61

Table S56. Association Between One-Indicator Supplement-Use Classes and Cesarean Delivery 62

Table S57. Association Between One-Indicator Supplement-Use Classes and Neonatal Physiological Jaundice 63

Table S58. Association Between One-Indicator Supplement-Use Classes and Length at Birth 64

Table S59. Association Between One-Indicator Supplement-Use Classes and Head Circumference Gain (40–60 d) 65

Table S60. Association Between One-Indicator Supplement-Use Classes and Length Gain (40–60 d) 66

Table S61. Association Between One-Indicator Supplement-Use Classes and Weight Gain (40–60 d)....67

Table S62. Logistic Model for Completion of the 40–60-Day Follow-up Used to Derive Inverse-Probability Weights 68

Table S63. Follow-up-Weighted Association Between Maternal Supplement-Use Patterns and Neonatal Physiological Jaundice....69

Table S64. Follow-up-Weighted Association Between Maternal Supplement-Use Patterns and Length Gain (40–60 d)....70

Table S65. Follow-up-Weighted Association Between Maternal Supplement-Use Patterns and Weight Gain (40–60 d) 71

Table S66. Follow-up-Weighted Association Between Maternal Supplement-Use Patterns and Head Circumference Gain (40–60 d) 72

Table S67. Model Fit Indices for the Latent Class Analysis Excluding Preterm Births....73

Figure S1. Model comparison for the cross-pregnancy latent class analysis....74

Figure S2. Observed mean supplement-use frequency by latent class 76

Figure S3. Sensitivity latent class analysis using cross-sectional binary indicators.. 78

Figure S4. Sensitivity latent class analysis using cross-sectional frequency indicators. 80

Figure S5. Odds of loss to follow-up at 40–60 days after birth....82

Figure S6. Sensitivity latent class analysis using period-specific binary indicators....84

Table S1. Classification of Provinces by Geographic Region in China

Region	Province
East China	Anhui, Jiangsu, Jiangxi, Shandong, Shanghai, Zhejiang, Fujian
North China	Beijing, Tianjin, Hebei, Shanxi, Inner Mongolia
Central China	Henan, Hubei, Hunan
South China	Guangdong, Guangxi, Hainan
Northeast China	Heilongjiang, Jilin, Liaoning
West China	Gansu, Ningxia, Xinjiang, Shaanxi, Guizhou, Sichuan, Tibet, Yunnan, Chongqing, Qinghai

Note: Geographic regions follow China's standard administrative grouping and were used as a covariate in adjusted analyses.

Table S2. Baseline Characteristics of Participants Completing Versus Lost to 40–60-Day Follow-up

Characteristic	Level	Completed FU (n = 1,447)	Lost to follow-up (n = 311)	OR (95% CI) for LTFU	P	P overall
Geographic region	North China	507 (35.0)	64 (20.6)	1 (Ref)	Ref	<0.001
Geographic region	East China	281 (19.4)	90 (28.9)	2.54 (1.79–3.62)	<0.001	
Geographic region	Central China	220 (15.2)	33 (10.6)	1.19 (0.75–1.85)	0.451	
Geographic region	South China	1 (0.1)	37 (11.9)	293.11 (61.86–5248.80)	<0.001	
Geographic region	Northeast China	200 (13.8)	9 (2.9)	0.36 (0.16–0.69)	0.005	
Geographic region	West China	238 (16.4)	78 (25.1)	2.60 (1.80–3.75)	<0.001	
Maternal age (years)	26–30	627 (43.3)	126 (40.5)	1 (Ref)	Ref	0.005
Maternal age (years)	18–25	151 (10.4)	38 (12.2)	1.25 (0.83–1.86)	0.275	
Maternal age (years)	31–35	521 (36.0)	95 (30.5)	0.91 (0.68–1.21)	0.512	
Maternal age (years)	≥36	148 (10.2)	52 (16.7)	1.75 (1.20–2.52)	0.003	
Pre-pregnancy BMI	Normal (18.5–25)	946 (70.3)	214 (75.6)	1 (Ref)	Ref	0.031
Pre-pregnancy BMI	Underweight (<18.5)	111 (8.3)	21 (7.4)	0.84 (0.50–1.34)	0.474	
Pre-pregnancy BMI	Overweight (25–30)	233 (17.3)	31 (11.0)	0.59 (0.39–0.87)	0.010	
Pre-pregnancy BMI	Obese (≥30)	55 (4.1)	17 (6.0)	1.37 (0.76–2.35)	0.278	
Maternal education	College or bachelor's	970 (67.2)	208 (66.9)	1 (Ref)	Ref	0.076
Maternal education	Primary school or below	8 (0.6)	6 (1.9)	3.50 (1.14–10.16)	0.022	
Maternal education	Junior high school	133 (9.2)	34 (10.9)	1.19 (0.78–1.77)	0.395	
Maternal education	Senior high / technical	230 (15.9)	47 (15.1)	0.95 (0.67–1.34)	0.786	

Maternal education	Master's or doctoral	103 (7.1)	16 (5.1)	0.72 (0.40–1.22)	0.249	
Folic acid use	Yes (any use)	1299 (90.2)	267 (86.1)	1 (Ref)	Ref	0.043
Folic acid use	No / almost never	141 (9.8)	43 (13.9)	1.48 (1.02–2.12)	0.035	

Note: Values are n (column %). OR = odds ratio of lost to follow-up versus completed follow-up (univariable logistic regression).

Table S3. Model Fit Indices for Cross-Pregnancy Latent Class Analysis of Maternal Supplement-Use Patterns

Classes	N	logLik	Parameters	AIC	BIC	aBIC	Entropy	Min class n	Min class %	Min AvePP	Min OCC	Converged	Delta BIC
2	1679	-31953.96	169	64245.91	65162.9	64626.01	0.947	421	25.1	0.972	32.94	Yes	5888.97
3	1679	-29720.56	254	59949.12	61327.32	60520.39	0.923	364	21.7	0.963	38.09	Yes	2053.39
4	1679	-28880.96	339	58439.93	60279.32	59202.37	0.926	177	10.5	0.948	37.44	Yes	1005.4
5	1679	-28253.07	424	57354.14	59654.74	58307.75	0.922	144	8.6	0.938	34.88	Yes	380.82
6	1679	-27747.06	509	56512.11	59273.92	57656.9	0.928	118	7.0	0.942	41.27	Yes	0.0
7	1679	-27437.53	594	56063.05	59286.07	57399.01	0.937	117	7.0	0.935	68.85	Yes	12.15
8	1679	-27153.56	679	55665.12	59349.34	57192.25	0.94	68	4.1	0.915	52.75	Yes	75.42
9	1679	-26901.64	764	55331.28	59476.7	57049.58	0.932	40	2.4	0.894	45.75	No	202.78
10	1679	-26624.96	849	54947.92	59554.55	56857.4	0.938	50	3.0	0.919	53.96	Yes	280.63

Note: Primary model selected k = 6 (lowest BIC among models with entropy ≥0.70, minimum class size ≥5%, and minimum AvePP ≥0.70).

Table S4. Classification Diagnostics for the Selected Six-Class Model of Maternal Supplement-Use Patterns

Class	Label	n	Percent	AvePP	OCC
C1	Mid-late moderate core	496	29.5	0.945	41.27
C2	Multi-supplement moderate persistent	140	8.3	0.942	174.93
C3	Multi-supplement high-frequency persistent	167	9.9	0.952	178.0
C4	All-supplement high-frequency	118	7.0	0.971	433.01
C5	Mid-late high-frequency core	326	19.4	0.954	86.64
C6	Low-use	432	25.7	0.952	57.46

Note: AvePP = average posterior probability; OCC = odds of correct classification.

Table S5. Baseline Characteristics by Maternal Supplement-Use Pattern

Characteristic	C1 Mid-late moderate core	C2 Multi-supplement moderate persistent	C3 Multi-supplement high-frequency persistent	C4 All-supplement high-frequency	C5 Mid-late high-frequency core	C6 Low-use
n	496	140	167	118	326	432
Age (years)	30.4 ± 3.8	30.7 ± 5.3	30.7 ± 4.1	31.1 ± 4.6	30.1 ± 4.3	30.1 ± 4.4
Pre–pregnancy BMI	22.4 ± 3.3	22.9 ± 3.9	22.3 ± 3.5	22.9 ± 4.1	22.7 ± 3.7	22.8 ± 3.6
Parity, multiparous	225 (45.4%)	81 (57.9%)	91 (54.5%)	69 (58.5%)	183 (56.1%)	246 (57.1%)
Anemia, yes	49 (9.9%)	10 (7.1%)	16 (9.6%)	2 (1.7%)	19 (5.8%)	18 (4.2%)
Gestational diabetes, yes	40 (8.1%)	5 (3.6%)	19 (11.4%)	14 (11.9%)	20 (6.1%)	22 (5.1%)
Gestational hypertension, yes	11 (2.2%)	1 (0.7%)	4 (2.4%)	2 (1.7%)	11 (3.4%)	8 (1.9%)
Infant sex, male	246 (49.6%)	77 (55.0%)	103 (61.7%)	54 (45.8%)	151 (46.3%)	216 (50.0%)

Note: Mean ± SD for continuous variables; n (%) for binary variables. Primary LCA complete-case cohort (N = 1,679).

Table S6. Outcome Event Counts and Rates by Maternal Supplement-Use Pattern

Outcome	C1 Mid-late moderate core	C2 Multi-supplement moderate persistent	C3 Multi-supplement high-frequency persistent	C4 All-supplement high-frequency	C5 Mid-late high-frequency core	C6 Low-use
Cesarean delivery	227/496 (45.8%)	76/140 (54.3%)	89/167 (53.3%)	72/118 (61.0%)	149/326 (45.7%)	223/431 (51.7%)
Infant eczema	142/335 (42.4%)	29/91 (31.9%)	54/116 (46.6%)	34/79 (43.0%)	82/232 (35.3%)	122/302 (40.4%)
Neonatal physiological jaundice	94/349 (26.9%)	22/98 (22.4%)	39/120 (32.5%)	27/83 (32.5%)	63/243 (25.9%)	73/321 (22.7%)
Macrosomia	21/491 (4.3%)	5/138 (3.6%)	12/166 (7.2%)	9/115 (7.8%)	18/324 (5.6%)	20/427 (4.7%)

Note: Includes cesarean delivery, macrosomia, neonatal physiological jaundice, and infant eczema.

Table S7. Association Between Maternal Supplement-Use Patterns and Cesarean Delivery

Pattern	Crude OR	Crude 95% CI	P value	Adjusted OR	Adjusted 95% CI	P value
C1 Mid-late moderate core	0.787	0.607-1.019	0.07	0.83	0.62-1.10	0.186
C2 Multi-supplement moderate persistent	1.108	0.756-1.626	0.6	1.15	0.76-1.76	0.509
C3 Multi-supplement high-frequency persistent	1.064	0.744-1.524	0.733	1.02	0.69-1.50	0.913
C4 All-supplement high-frequency	1.46	0.967-2.222	0.074	1.33	0.85-2.11	0.216
C5 Mid-late high-frequency core	0.785	0.588-1.047	0.1	0.72	0.53-0.99	0.0441
C6 Low-use	1	—	—	1	—	—

Note: Crude and adjusted odds ratios. Adjusted for maternal age, prepregnancy BMI, education, geographic region, parity, infant sex, gestational anemia, gestational hypertension, and gestational diabetes. Reference = C6 Low-use.

Table S8. Association Between Maternal Supplement-Use Patterns and Macrosomia

Pattern	Crude OR	Crude 95% CI	P value	Adjusted OR	Adjusted 95% CI	P value
C1 Mid-late moderate core	0.909	0.484-1.712	0.766	0.80	0.40-1.64	0.546
C2 Multi-supplement moderate persistent	0.765	0.251-1.932	0.599	0.64	0.19-1.75	0.411
C3 Multi-supplement high-frequency persistent	1.586	0.737-3.279	0.222	1.33	0.57-3.00	0.491
C4 All-supplement high-frequency	1.728	0.73-3.801	0.189	1.51	0.57-3.76	0.385
C5 Mid-late high-frequency core	1.197	0.617-2.306	0.59	1.04	0.49-2.20	0.909
C6 Low-use	1	—	—	1	—	—

Note: Crude and adjusted odds ratios. Adjusted for maternal age, prepregnancy BMI, education, geographic region, parity, infant sex, gestational anemia, gestational hypertension, and gestational diabetes. Reference = C6 Low-use.

Table S9. Association Between Maternal Supplement-Use Patterns and Neonatal Physiological Jaundice

Pattern	Crude OR	Crude 95% CI	P value	Adjusted OR	Adjusted 95% CI	P value
C1 Mid-late moderate core	1.252	0.881-1.784	0.211	1.33	0.91-1.95	0.14
C2 Multi-supplement moderate persistent	0.983	0.563-1.669	0.952	1.06	0.58-1.90	0.843
C3 Multi-supplement high-frequency persistent	1.636	1.025-2.59	0.037	1.65	1.00-2.70	0.049
C4 All-supplement high-frequency	1.638	0.957-2.761	0.067	1.86	1.04-3.27	0.0344
C5 Mid-late high-frequency core	1.189	0.806-1.752	0.382	1.24	0.82-1.88	0.304
C6 Low-use	1	—	—	1	—	—

Note: Crude and adjusted odds ratios. Adjusted for maternal age, prepregnancy BMI, education, geographic region, parity, infant sex, gestational anemia, gestational hypertension, and gestational diabetes. Reference = C6 Low-use.

Table S10. Association Between Maternal Supplement-Use Patterns and Infant Eczema

Pattern	Crude OR	Crude 95% CI	P value	Adjusted OR	Adjusted 95% CI	P value
C1 Mid-late moderate core	1.086	0.792-1.49	0.611	1.09	0.78-1.54	0.615
C2 Multi-supplement moderate persistent	0.69	0.415-1.126	0.144	0.58	0.33-1.01	0.06
C3 Multi-supplement high-frequency persistent	1.285	0.834-1.978	0.254	1.38	0.87-2.19	0.165
C4 All-supplement high-frequency	1.115	0.672-1.836	0.671	1.29	0.75-2.21	0.36
C5 Mid-late high-frequency core	0.807	0.565-1.148	0.234	0.84	0.58-1.23	0.377
C6 Low-use	1	—	—	1	—	—

Note: Crude and adjusted odds ratios. Adjusted for maternal age, prepregnancy BMI, education, geographic region, parity, infant sex, gestational anemia, gestational hypertension, and gestational diabetes. Reference = C6 Low-use.

Table S11. Association Between Maternal Supplement-Use Patterns and Length at Birth

Pattern	Adjusted β	95% CI	P value
C1 Mid-late moderate core	-0.10	-0.33 to 0.13	0.382
C2 Multi-supplement moderate persistent	-0.02	-0.36 to 0.32	0.908
C3 Multi-supplement high-frequency persistent	-0.03	-0.33 to 0.28	0.87
C4 All-supplement high-frequency	-0.46	-0.81 to -0.10	0.0117
C5 Mid-late high-frequency core	-0.06	-0.32 to 0.20	0.646
C6 Low-use	0	—	—

Note: Adjusted β (95% CI). Adjusted for gestational age, maternal age, prepregnancy BMI, education, geographic region, parity, infant sex, gestational anemia, gestational hypertension, and gestational diabetes. Reference = C6 Low-use.

Table S12. Association Between Maternal Supplement-Use Patterns and Weight at Birth

Pattern	Adjusted β	95% CI	P value
C1 Mid-late moderate core	-35.64	-90.66 to 19.38	0.204
C2 Multi-supplement moderate persistent	-65.14	-147.21 to 16.93	0.12
C3 Multi-supplement high-frequency persistent	10.07	-65.01 to 85.14	0.793
C4 All-supplement high-frequency	-41.36	-128.09 to 45.37	0.35
C5 Mid-late high-frequency core	15.57	-45.88 to 77.03	0.619
C6 Low-use	0	—	—

Note: Adjusted β (95% CI). Adjusted for gestational age, maternal age, prepregnancy BMI, education, geographic region, parity, infant sex, gestational anemia, gestational hypertension, and gestational diabetes. Reference = C6 Low-use.

Table S13. Association Between Maternal Supplement-Use Patterns and Head Circumference at Birth

Pattern	Adjusted β	95% CI	P value
C1 Mid-late moderate core	-0.10	-0.32 to 0.12	0.374
C2 Multi-supplement moderate persistent	-0.22	-0.55 to 0.11	0.196
C3 Multi-supplement high-frequency persistent	-0.06	-0.37 to 0.25	0.695
C4 All-supplement high-frequency	-0.31	-0.65 to 0.04	0.0828
C5 Mid-late high-frequency core	-0.21	-0.46 to 0.05	0.109
C6 Low-use	0	—	—

Note: Adjusted β (95% CI). Adjusted for gestational age, maternal age, prepregnancy BMI, education, geographic region, parity, infant sex, gestational anemia, gestational hypertension, and gestational diabetes. Reference = C6 Low-use.

Table S14. Association Between Maternal Supplement-Use Patterns and Length Gain (40-60d)

Pattern	Adjusted β	95% CI	P value
C1 Mid-late moderate core	0.192	-0.388 to 0.773	0.516
C2 Multi-supplement moderate persistent	0.275	-0.596 to 1.147	0.535
C3 Multi-supplement high-frequency persistent	0.309	-0.468 to 1.087	0.435
C4 All-supplement high-frequency	-0.481	-1.409 to 0.448	0.31
C5 Mid-late high-frequency core	0.207	-0.442 to 0.855	0.532
C6 Low-use	0	—	—

Note: Adjusted β (95% CI). Adjusted for birth length, gestational age, maternal age, prepregnancy BMI, education, geographic region, parity, infant sex, gestational anemia, gestational hypertension, and gestational diabetes. Reference = C6 Low-use.

Table S15. Association Between Maternal Supplement-Use Patterns and Weight Gain (40-60d)

Pattern	Adjusted β	95% CI	P value
C1 Mid-late moderate core	104.736	-69.096 to 278.568	0.237
C2 Multi-supplement moderate persistent	116.184	-147.941 to 380.310	0.388
C3 Multi-supplement high-frequency persistent	72.103	-160.942 to 305.148	0.544
C4 All-supplement high-frequency	-77.174	-355.545 to 201.197	0.587
C5 Mid-late high-frequency core	32.312	-157.253 to 221.876	0.738
C6 Low-use	0	—	—

Note: Adjusted β (95% CI). Adjusted for birth weight, gestational age, maternal age, prepregnancy BMI, education, geographic region, parity, infant sex, gestational anemia, gestational hypertension, and gestational diabetes. Reference = C6 Low-use.

Table S16. Association Between Maternal Supplement-Use Patterns and Head Circumference Gain (40-60d)

Pattern	Adjusted β	95% CI	P value
C1 Mid-late moderate core	0.873	0.344-1.402	0.00126
C2 Multi-supplement moderate persistent	0.487	-0.344-1.318	0.25
C3 Multi-supplement high-frequency persistent	0.886	0.148-1.624	0.0187
C4 All-supplement high-frequency	0.482	-0.366-1.330	0.264
C5 Mid-late high-frequency core	0.381	-0.228-0.990	0.22
C6 Low-use	0	—	—

Note: Adjusted β (95% CI). Adjusted for birth head circumference, gestational age, maternal age, prepregnancy BMI, education, geographic region, parity, infant sex, gestational anemia, gestational hypertension, and gestational diabetes. Reference = C6 Low-use.

Table S17. Multivariable Multinomial Logistic Regression for Maternal Supplement-Use Pattern Membership

Variable	Category	C1 RRR (95% CI)	C2 RRR (95% CI)	C3 RRR (95% CI)	C4 RRR (95% CI)	C5 RRR (95% CI)
Age (years)	18~25	0.571 (0.3-1.089)	0.329 (0.136-0.8)	0.425 (0.17-1.061)	0.285 (0.118-0.688)	0.794 (0.41-1.539)
	26~30	1.227 (0.743-2.028)	0.544 (0.281-1.05)	0.944 (0.493-1.81)	0.434 (0.22-0.857)	0.947 (0.548-1.637)
	31~35	1.058 (0.646-1.733)	0.558 (0.294-1.06)	0.816 (0.431-1.547)	0.465 (0.239-0.903)	0.804 (0.469-1.379)
	≥36 (Ref)	1	1	1	1	1
Pre–pregnancy BMI	Underweight (<18.5)	1.967 (0.793-4.881)	0.526 (0.159-1.743)	1.319 (0.442-3.937)	1.306 (0.4-4.261)	1.854 (0.733-4.692)
	Normal (18.5–25)	1.857 (0.858-4.02)	0.664 (0.274-1.606)	0.911 (0.363-2.284)	0.893 (0.352-2.264)	1.326 (0.609-2.89)
	Overweight (25–30)	1.963 (0.861-4.477)	0.608 (0.226-1.637)	0.795 (0.289-2.19)	0.988 (0.356-2.743)	1.371 (0.591-3.177)
	Obese (≥30) (Ref)	1	1	1	1	1
Education level	Primary school or below	0.23 (0.022-2.415)	0.897 (0.074-10.817)	0.57 (0.052-6.228)	3.655 (0.374-35.714)	0.353 (0.032-3.839)
	Junior high school	0.591 (0.291-1.198)	0.54 (0.17-1.71)	0.263 (0.09-0.763)	2.438 (0.618-9.622)	0.537 (0.232-1.245)
	High school / vocational school	0.63 (0.332-1.196)	0.744 (0.262-2.109)	0.358 (0.145-0.884)	2.38 (0.632-8.972)	0.994 (0.474-2.083)
	College or bachelor’s degree	0.871 (0.509-1.492)	1.169 (0.476-2.871)	0.784 (0.39-1.579)	1.754 (0.501-6.142)	0.981 (0.509-1.891)
	Master’s degree or above (Ref)	1	1	1	1	1
Parity	Nulliparous	1.523 (1.127-2.058)	1.111 (0.709-1.741)	1.058 (0.702-1.596)	1.202 (0.741-1.952)	1.036 (0.739-1.454)
	Multiparous (Ref)	1	1	1	1	1
Gestational hypertension	No	1.057 (0.365-3.059)	2.149 (0.25-18.458)	0.738 (0.197-2.766)	1.066 (0.199-5.703)	0.501 (0.173-1.451)
	Yes (Ref)	1	1	1	1	1
Gestational diabetes	No	0.717 (0.408-1.258)	1.631 (0.592-4.494)	0.577 (0.29-1.146)	0.354 (0.169-0.746)	0.902 (0.468-1.738)
	Yes (Ref)	1	1	1	1	1
Anemia	No	0.47 (0.261-0.847)	0.53 (0.225-1.248)	0.506 (0.239-1.074)	2.762 (0.606-12.596)	0.805 (0.393-1.652)
	Yes (Ref)	1	1	1	1	1
Region	North China	0.763 (0.522-1.114)	1.963 (1.034-3.727)	0.895 (0.522-1.536)	3.417 (1.547-7.546)	1.588 (1.03-2.451)
	Central China	0.552 (0.357-0.852)	0.968 (0.448-2.092)	0.819 (0.451-1.489)	1.69 (0.674-4.241)	0.475 (0.267-0.846)
	South China	1.798 (0.442-7.308)	9.405 (1.98-44.665)	1.919 (0.299-12.336)	10.517 (1.962-56.372)	4.9 (1.244-19.297)
	Northeast China	0.522 (0.299-0.908)	2.825 (1.31-6.09)	1.191 (0.588-2.415)	5.397 (2.225-13.092)	1.243 (0.69-2.239)
	West China	0.607 (0.391-0.942)	1.893 (0.927-3.867)	1.093 (0.606-1.971)	2.301 (0.961-5.51)	0.935 (0.561-1.557)
	East China (Ref)	1	1	1	1	1

Note: Relative risk ratios (RRR) relative to C6 Low-use. Education codes 0 and 1 were merged as Primary school or below. Reference levels are Age ≥36, Obese BMI, Master’s degree or above, Multiparous, Yes for gestational hypertension/diabetes/anemia, and East China.

Table S18. Model Fit Indices for Sensitivity Latent Class Analyses of Maternal Supplement Use

Analysis	Classes	BIC	aBIC	Entropy	Min class %	Min AvePP	Delta BIC
Cross-sectional binary LCA	2	15080.42	15013.7	0.714	21.1	0.904	361.06
Cross-sectional binary LCA	3	14838.11	14736.45	0.752	11.2	0.791	118.75
Cross-sectional binary LCA	4	14727.05	14590.45	0.708	7.8	0.786	7.69
Cross-sectional binary LCA	5	14719.36	14547.81	0.744	2.9	0.772	0.0
Cross-sectional binary LCA	6	14722.91	14516.41	0.688	2.6	0.706	3.55
Cross-sectional ordinal-frequency LCA	2	28790.1	28596.31	0.882	24.2	0.947	1538.9
Cross-sectional ordinal-frequency LCA	3	27745.16	27452.89	0.815	22.6	0.902	493.96
Cross-sectional ordinal-frequency LCA	4	27434.92	27044.17	0.79	15.2	0.833	183.72
Cross-sectional ordinal-frequency LCA	5	27258.97	26769.73	0.824	4.6	0.827	7.76
Cross-sectional ordinal-frequency LCA	6	27251.2	26663.48	0.834	2.5	0.845	0.0

Note: Sensitivity analyses ignored trimester timing. Selected k = 4 in both binary and ordinal-frequency specifications.

Table S19. Cross-Classification of Primary and Binary Sensitivity Latent Classes of Maternal Supplement Use

Primary class	1	2	3	4	All
C1 Mid-late moderate core	14	42	50	387	493
C2 Multi-supplement moderate persistent	27	57	0	54	138
C3 Multi-supplement high-frequency persistent	35	99	1	29	164
C4 All-supplement high-frequency	34	44	0	39	117
C5 Mid-late high-frequency core	7	24	43	249	323
C6 Low-use	12	30	173	214	429
All	129	296	267	972	1664

Table S20. Cross-Classification of Primary and Ordinal-Frequency Sensitivity Latent Classes of Maternal Supplement Use

Primary class	1	2	3	4	All
C1 Mid-late moderate core	1	14	113	365	493
C2 Multi-supplement moderate persistent	4	107	5	22	138
C3 Multi-supplement high-frequency persistent	0	0	0	164	164
C4 All-supplement high-frequency	115	1	0	1	117
C5 Mid-late high-frequency core	231	6	37	49	323
C6 Low-use	10	121	289	9	429
All	361	249	444	610	1664

Table S21. Univariate Analysis of Calcium Supplement Use

Variable	Category	Total n	Never used n (%)	Used n (%)	χ^2	P value
Age (years)	18~25	189	34 (18.0%)	155 (82.0%)	18.029	<0.001
	26~30	751	60 (8.0%)	691 (92.0%)		
	31~35	612	73 (11.9%)	539 (88.1%)		
	≥36	200	18 (9.0%)	182 (91.0%)		
Pre–pregnancy BMI	Underweight (<18.5)	131	11 (8.4%)	120 (91.6%)	2.245	0.523
	Normal (18.5–25)	1158	117 (10.1%)	1041 (89.9%)		
	Overweight (25–30)	264	33 (12.5%)	231 (87.5%)		
	Obese (≥30)	72	9 (12.5%)	63 (87.5%)		
Education level	Primary school or below	14	6 (42.9%)	8 (57.1%)	34.746	<0.001
	Junior high school	166	26 (15.7%)	140 (84.3%)		
	High school / vocational school	277	44 (15.9%)	233 (84.1%)		
	College or bachelor's degree	1176	98 (8.3%)	1078 (91.7%)		
	Master's degree or above	119	11 (9.2%)	108 (90.8%)		
Parity	Nulliparous	814	80 (9.8%)	734 (90.2%)	0.619	0.431
	Multiparous	937	104 (11.1%)	833 (88.9%)		
Gestational hypertension	No	1711	183 (10.7%)	1528 (89.3%)	–	0.308
	Yes	41	2 (4.9%)	39 (95.1%)		
Gestational diabetes	No	1623	171 (10.5%)	1452 (89.5%)	0	1
	Yes	129	14 (10.9%)	115 (89.1%)		
Anemia	No	1633	174 (10.7%)	1459 (89.3%)	0.108	0.742
	Yes	119	11 (9.2%)	108 (90.8%)		
Region	East China	370	32 (8.6%)	338 (91.4%)	3.838	0.573
	North China	571	60 (10.5%)	511 (89.5%)		
	Central China	252	30 (11.9%)	222 (88.1%)		
	South China	38	2 (5.3%)	36 (94.7%)		
	Northeast China	206	25 (12.1%)	181 (87.9%)		
	West China	315	36 (11.4%)	279 (88.6%)		
GDP level	High GDP	800	75 (9.4%)	725 (90.6%)	1.962	0.161
	Low GDP	952	110 (11.6%)	842 (88.4%)		

Table S22. Univariate Analysis of Iron Supplement Use

Variable	Category	Total n	Never used n (%)	Used n (%)	χ^2	P value
Age (years)	18~25	188	54 (28.7%)	134 (71.3%)	4.371	0.224
	26~30	751	163 (21.7%)	588 (78.3%)		
	31~35	610	140 (23.0%)	470 (77.0%)		
	≥36	200	49 (24.5%)	151 (75.5%)		
Pre–pregnancy BMI	Underweight (<18.5)	129	35 (27.1%)	94 (72.9%)	9.474	0.024
	Normal (18.5–25)	1158	254 (21.9%)	904 (78.1%)		
	Overweight (25–30)	264	56 (21.2%)	208 (78.8%)		
	Obese (≥30)	72	26 (36.1%)	46 (63.9%)		
Education level	Primary school or below	14	5 (35.7%)	9 (64.3%)	5.612	0.23
	Junior high school	165	40 (24.2%)	125 (75.8%)		
	High school / vocational school	276	75 (27.2%)	201 (72.8%)		
	College or bachelor's degree	1175	264 (22.5%)	911 (77.5%)		
	Master's degree or above	119	22 (18.5%)	97 (81.5%)		
Parity	Nulliparous	812	180 (22.2%)	632 (77.8%)	0.753	0.385
	Multiparous	936	225 (24.0%)	711 (76.0%)		
Gestational hypertension	No	1708	396 (23.2%)	1312 (76.8%)	0	1
	Yes	41	10 (24.4%)	31 (75.6%)		
Gestational diabetes	No	1622	372 (22.9%)	1250 (77.1%)	0.769	0.38
	Yes	127	34 (26.8%)	93 (73.2%)		
Anemia	No	1630	399 (24.5%)	1231 (75.5%)	20.486	<0.001
	Yes	119	7 (5.9%)	112 (94.1%)		
Region	East China	369	82 (22.2%)	287 (77.8%)	76.504	<0.001
	North China	570	78 (13.7%)	492 (86.3%)		
	Central China	253	90 (35.6%)	163 (64.4%)		
	South China	37	6 (16.2%)	31 (83.8%)		
	Northeast China	206	41 (19.9%)	165 (80.1%)		
	West China	314	109 (34.7%)	205 (65.3%)		
GDP level	High GDP	799	233 (29.2%)	566 (70.8%)	28.587	<0.001
	Low GDP	950	173 (18.2%)	777 (81.8%)		

Table S23. Univariate Analysis of Zinc Supplement Use

Variable	Category	Total n	Never used n (%)	Used n (%)	χ^2	P value
Age (years)	18~25	188	163 (86.7%)	25 (13.3%)	7.752	0.051
	26~30	746	628 (84.2%)	118 (15.8%)		
	31~35	608	483 (79.4%)	125 (20.6%)		
	≥36	200	164 (82.0%)	36 (18.0%)		
Pre–pregnancy BMI	Underweight (<18.5)	129	104 (80.6%)	25 (19.4%)	0.529	0.913
	Normal (18.5–25)	1152	952 (82.6%)	200 (17.4%)		
	Overweight (25–30)	263	217 (82.5%)	46 (17.5%)		
	Obese (≥30)	71	60 (84.5%)	11 (15.5%)		
Education level	Primary school or below	14	11 (78.6%)	3 (21.4%)	0.739	0.946
	Junior high school	166	134 (80.7%)	32 (19.3%)		
	High school / vocational school	275	228 (82.9%)	47 (17.1%)		
	College or bachelor's degree	1168	968 (82.9%)	200 (17.1%)		
	Master's degree or above	119	97 (81.5%)	22 (18.5%)		
Parity	Nulliparous	809	688 (85.0%)	121 (15.0%)	6.257	0.012
	Multiparous	932	749 (80.4%)	183 (19.6%)		
Gestational hypertension	No	1702	1403 (82.4%)	299 (17.6%)	0.389	0.533
	Yes	40	35 (87.5%)	5 (12.5%)		
Gestational diabetes	No	1615	1329 (82.3%)	286 (17.7%)	0.791	0.374
	Yes	127	109 (85.8%)	18 (14.2%)		
Anemia	No	1624	1334 (82.1%)	290 (17.9%)	2.342	0.126
	Yes	118	104 (88.1%)	14 (11.9%)		
Region	East China	369	287 (77.8%)	82 (22.2%)	11.689	0.039
	North China	569	491 (86.3%)	78 (13.7%)		
	Central China	253	208 (82.2%)	45 (17.8%)		
	South China	37	31 (83.8%)	6 (16.2%)		
	Northeast China	202	164 (81.2%)	38 (18.8%)		
	West China	312	257 (82.4%)	55 (17.6%)		
GDP level	High GDP	797	636 (79.8%)	161 (20.2%)	7.362	0.007
	Low GDP	945	802 (84.9%)	143 (15.1%)		

Table S24. Univariate Analysis of Fish Oil Supplement Use

Variable	Category	Total n	Never used n (%)	Used n (%)	χ²	P value
Age (years)	18~25	188	142 (75.5%)	46 (24.5%)	5.599	0.133
	26~30	748	565 (75.5%)	183 (24.5%)		
	31~35	607	446 (73.5%)	161 (26.5%)		
	≥36	200	135 (67.5%)	65 (32.5%)		
Pre–pregnancy BMI	Underweight (<18.5)	129	95 (73.6%)	34 (26.4%)	1.228	0.746
	Normal (18.5–25)	1153	853 (74.0%)	300 (26.0%)		
	Overweight (25–30)	263	194 (73.8%)	69 (26.2%)		
	Obese (≥30)	72	49 (68.1%)	23 (31.9%)		
Education level	Primary school or below	14	10 (71.4%)	4 (28.6%)	2.935	0.569
	Junior high school	165	125 (75.8%)	40 (24.2%)		
	High school / vocational school	277	201 (72.6%)	76 (27.4%)		
	College or bachelor's degree	1168	857 (73.4%)	311 (26.6%)		
	Master's degree or above	119	95 (79.8%)	24 (20.2%)		
Parity	Nulliparous	810	613 (75.7%)	197 (24.3%)	2.366	0.124
	Multiparous	932	674 (72.3%)	258 (27.7%)		
Gestational hypertension	No	1703	1252 (73.5%)	451 (26.5%)	4.683	0.03
	Yes	40	36 (90.0%)	4 (10.0%)		
Gestational diabetes	No	1617	1203 (74.4%)	414 (25.6%)	2.567	0.109
	Yes	126	85 (67.5%)	41 (32.5%)		
Anemia	No	1624	1192 (73.4%)	432 (26.6%)	2.675	0.102
	Yes	119	96 (80.7%)	23 (19.3%)		
Region	East China	369	293 (79.4%)	76 (20.6%)	14.695	0.012
	North China	570	427 (74.9%)	143 (25.1%)		
	Central China	253	183 (72.3%)	70 (27.7%)		
	South China	37	23 (62.2%)	14 (37.8%)		
	Northeast China	201	134 (66.7%)	67 (33.3%)		
	West China	313	228 (72.8%)	85 (27.2%)		
GDP level	High GDP	798	594 (74.4%)	204 (25.6%)	0.174	0.676
	Low GDP	945	694 (73.4%)	251 (26.6%)		

Table S25. Univariate Analysis of Vitamin E Supplement Use

Variable	Category	Total n	Never used n (%)	Used n (%)	χ^2	P value
Age (years)	18~25	188	148 (78.7%)	40 (21.3%)	2.427	0.489
	26~30	745	612 (82.1%)	133 (17.9%)		
	31~35	604	498 (82.5%)	106 (17.5%)		
	≥36	199	157 (78.9%)	42 (21.1%)		
Pre–pregnancy BMI	Underweight (<18.5)	129	105 (81.4%)	24 (18.6%)	2.363	0.501
	Normal (18.5–25)	1146	942 (82.2%)	204 (17.8%)		
	Overweight (25–30)	263	215 (81.7%)	48 (18.3%)		
	Obese (≥30)	72	54 (75.0%)	18 (25.0%)		
Education level	Primary school or below	14	9 (64.3%)	5 (35.7%)	15.823	0.003
	Junior high school	165	121 (73.3%)	44 (26.7%)		
	High school / vocational school	274	225 (82.1%)	49 (17.9%)		
	College or bachelor’s degree	1164	953 (81.9%)	211 (18.1%)		
	Master’s degree or above	119	107 (89.9%)	12 (10.1%)		
Parity	Nulliparous	806	673 (83.5%)	133 (16.5%)	3.75	0.053
	Multiparous	929	741 (79.8%)	188 (20.2%)		
Gestational hypertension	No	1696	1381 (81.4%)	315 (18.6%)	0.136	0.712
	Yes	40	34 (85.0%)	6 (15.0%)		
Gestational diabetes	No	1610	1313 (81.6%)	297 (18.4%)	0.002	0.962
	Yes	126	102 (81.0%)	24 (19.0%)		
Anemia	No	1618	1319 (81.5%)	299 (18.5%)	0	1
	Yes	118	96 (81.4%)	22 (18.6%)		
Region	East China	369	327 (88.6%)	42 (11.4%)	66.011	<0.001
	North China	565	489 (86.5%)	76 (13.5%)		
	Central China	251	208 (82.9%)	43 (17.1%)		
	South China	37	30 (81.1%)	7 (18.9%)		
	Northeast China	202	139 (68.8%)	63 (31.2%)		
	West China	312	222 (71.2%)	90 (28.8%)		
GDP level	High GDP	795	659 (82.9%)	136 (17.1%)	1.698	0.193
	Low GDP	941	756 (80.3%)	185 (19.7%)		

Table S26. Univariate Analysis of Vitamin C Supplement Use

Variable	Category	Total n	Never used n (%)	Used n (%)	χ^2	P value
Age (years)	18~25	184	123 (66.8%)	61 (33.2%)	2.048	0.563
	26~30	748	469 (62.7%)	279 (37.3%)		
	31~35	601	393 (65.4%)	208 (34.6%)		
	≥36	200	124 (62.0%)	76 (38.0%)		
Pre–pregnancy BMI	Underweight (<18.5)	129	91 (70.5%)	38 (29.5%)	3.859	0.277
	Normal (18.5–25)	1146	726 (63.4%)	420 (36.6%)		
	Overweight (25–30)	263	173 (65.8%)	90 (34.2%)		
	Obese (≥30)	70	41 (58.6%)	29 (41.4%)		
Education level	Primary school or below	14	6 (42.9%)	8 (57.1%)	8.068	0.089
	Junior high school	164	101 (61.6%)	63 (38.4%)		
	High school / vocational school	275	174 (63.3%)	101 (36.7%)		
	College or bachelor's degree	1162	741 (63.8%)	421 (36.2%)		
	Master's degree or above	118	87 (73.7%)	31 (26.3%)		
Parity	Nulliparous	805	527 (65.5%)	278 (34.5%)	1.232	0.267
	Multiparous	927	582 (62.8%)	345 (37.2%)		
Gestational hypertension	No	1695	1082 (63.8%)	613 (36.2%)	0.556	0.456
	Yes	38	27 (71.1%)	11 (28.9%)		
Gestational diabetes	No	1608	1020 (63.4%)	588 (36.6%)	2.709	0.1
	Yes	125	89 (71.2%)	36 (28.8%)		
Anemia	No	1614	1040 (64.4%)	574 (35.6%)	1.733	0.188
	Yes	119	69 (58.0%)	50 (42.0%)		
Region	East China	367	269 (73.3%)	98 (26.7%)	69.899	<0.001
	North China	565	328 (58.1%)	237 (41.9%)		
	Central China	251	194 (77.3%)	57 (22.7%)		
	South China	37	26 (70.3%)	11 (29.7%)		
	Northeast China	203	94 (46.3%)	109 (53.7%)		
	West China	310	198 (63.9%)	112 (36.1%)		
GDP level	High GDP	792	572 (72.2%)	220 (27.8%)	42.212	<0.001
	Low GDP	941	537 (57.1%)	404 (42.9%)		

Table S27. Univariate Analysis of Folic Acid Supplement Use

Variable	Category	Total n	Never used n (%)	Used n (%)	χ^2	P value
Age (years)	18~25	188	25 (13.3%)	163 (86.7%)	4.651	0.199
	26~30	750	70 (9.3%)	680 (90.7%)		
	31~35	612	62 (10.1%)	550 (89.9%)		
	≥36	200	27 (13.5%)	173 (86.5%)		
Pre–pregnancy BMI	Underweight (<18.5)	130	16 (12.3%)	114 (87.7%)	2.809	0.422
	Normal (18.5–25)	1158	114 (9.8%)	1044 (90.2%)		
	Overweight (25–30)	263	26 (9.9%)	237 (90.1%)		
	Obese (≥30)	72	11 (15.3%)	61 (84.7%)		
Education level	Primary school or below	14	3 (21.4%)	11 (78.6%)	7.205	0.125
	Junior high school	166	21 (12.7%)	145 (87.3%)		
	High school / vocational school	277	38 (13.7%)	239 (86.3%)		
	College or bachelor's degree	1174	111 (9.5%)	1063 (90.5%)		
	Master's degree or above	119	11 (9.2%)	108 (90.8%)		
Parity	Nulliparous	813	74 (9.1%)	739 (90.9%)	2.97	0.085
	Multiparous	936	110 (11.8%)	826 (88.2%)		
Gestational hypertension	No	1709	180 (10.5%)	1529 (89.5%)	–	1
	Yes	41	4 (9.8%)	37 (90.2%)		
Gestational diabetes	No	1621	172 (10.6%)	1449 (89.4%)	0.101	0.751
	Yes	129	12 (9.3%)	117 (90.7%)		
Anemia	No	1631	172 (10.5%)	1459 (89.5%)	0	0.997
	Yes	119	12 (10.1%)	107 (89.9%)		
Region	East China	370	44 (11.9%)	326 (88.1%)	2.239	0.815
	North China	569	62 (10.9%)	507 (89.1%)		
	Central China	253	24 (9.5%)	229 (90.5%)		
	South China	38	4 (10.5%)	34 (89.5%)		
	Northeast China	206	17 (8.3%)	189 (91.7%)		
	West China	314	33 (10.5%)	281 (89.5%)		
GDP level	High GDP	800	89 (11.1%)	711 (88.9%)	0.471	0.493
	Low GDP	950	95 (10.0%)	855 (90.0%)		

Table S28. Univariate Analysis of MMS Supplement Use

Variable	Category	Total n	Never used n (%)	Used n (%)	χ²	P value
Age (years)	18~25	187	165 (88.2%)	22 (11.8%)	13.405	0.004
	26~30	748	657 (87.8%)	91 (12.2%)		
	31~35	607	501 (82.5%)	106 (17.5%)		
	≥36	198	158 (79.8%)	40 (20.2%)		
Pre–pregnancy BMI	Underweight (<18.5)	129	110 (85.3%)	19 (14.7%)	9.658	0.022
	Normal (18.5–25)	1151	962 (83.6%)	189 (16.4%)		
	Overweight (25–30)	262	238 (90.8%)	24 (9.2%)		
	Obese (≥30)	71	63 (88.7%)	8 (11.3%)		
Education level	Primary school or below	14	12 (85.7%)	2 (14.3%)	7.73	0.102
	Junior high school	164	145 (88.4%)	19 (11.6%)		
	High school / vocational school	276	247 (89.5%)	29 (10.5%)		
	College or bachelor's degree	1168	980 (83.9%)	188 (16.1%)		
	Master's degree or above	118	97 (82.2%)	21 (17.8%)		
Parity	Nulliparous	805	695 (86.3%)	110 (13.7%)	1.61	0.204
	Multiparous	934	785 (84.0%)	149 (16.0%)		
Gestational hypertension	No	1701	1444 (84.9%)	257 (15.1%)	2.262	0.133
	Yes	39	37 (94.9%)	2 (5.1%)		
Gestational diabetes	No	1614	1380 (85.5%)	234 (14.5%)	2.229	0.135
	Yes	126	101 (80.2%)	25 (19.8%)		
Anemia	No	1622	1372 (84.6%)	250 (15.4%)	4.667	0.031
	Yes	118	109 (92.4%)	9 (7.6%)		
Region	East China	366	322 (88.0%)	44 (12.0%)	27.881	<0.001
	North China	569	512 (90.0%)	57 (10.0%)		
	Central China	250	200 (80.0%)	50 (20.0%)		
	South China	37	31 (83.8%)	6 (16.2%)		
	Northeast China	204	162 (79.4%)	42 (20.6%)		
	West China	314	254 (80.9%)	60 (19.1%)		
GDP level	High GDP	793	649 (81.8%)	144 (18.2%)	11.856	<0.001
	Low GDP	947	832 (87.9%)	115 (12.1%)		

Table S29. Univariate Analysis of DHA Supplement Use

Variable	Category	Total n	Never used n (%)	Used n (%)	χ^2	P value
Age (years)	18~25	188	122 (64.9%)	66 (35.1%)	6.223	0.101
	26~30	747	443 (59.3%)	304 (40.7%)		
	31~35	606	357 (58.9%)	249 (41.1%)		
	≥36	200	105 (52.5%)	95 (47.5%)		
Pre–pregnancy BMI	Underweight (<18.5)	130	70 (53.8%)	60 (46.2%)	2.787	0.426
	Normal (18.5–25)	1152	671 (58.2%)	481 (41.8%)		
	Overweight (25–30)	264	157 (59.5%)	107 (40.5%)		
	Obese (≥30)	70	46 (65.7%)	24 (34.3%)		
Education level	Primary school or below	14	7 (50.0%)	7 (50.0%)	12.302	0.015
	Junior high school	165	109 (66.1%)	56 (33.9%)		
	High school / vocational school	275	180 (65.5%)	95 (34.5%)		
	College or bachelor's degree	1168	658 (56.3%)	510 (43.7%)		
	Master's degree or above	119	73 (61.3%)	46 (38.7%)		
Parity	Nulliparous	808	464 (57.4%)	344 (42.6%)	1.47	0.225
	Multiparous	932	563 (60.4%)	369 (39.6%)		
Gestational hypertension	No	1701	996 (58.6%)	705 (41.4%)	5.042	0.025
	Yes	40	31 (77.5%)	9 (22.5%)		
Gestational diabetes	No	1616	961 (59.5%)	655 (40.5%)	1.866	0.172
	Yes	125	66 (52.8%)	59 (47.2%)		
Anemia	No	1622	955 (58.9%)	667 (41.1%)	0.063	0.801
	Yes	119	72 (60.5%)	47 (39.5%)		
Region	East China	368	242 (65.8%)	126 (34.2%)	61.292	<0.001
	North China	567	375 (66.1%)	192 (33.9%)		
	Central China	252	123 (48.8%)	129 (51.2%)		
	South China	37	23 (62.2%)	14 (37.8%)		
	Northeast China	204	81 (39.7%)	123 (60.3%)		
	West China	313	183 (58.5%)	130 (41.5%)		
GDP level	High GDP	796	448 (56.3%)	348 (43.7%)	4.24	0.039
	Low GDP	945	579 (61.3%)	366 (38.7%)		

Table S30. Univariate Analysis of Selenium Supplement Use

Variable	Category	Total n	Never used n (%)	Used n (%)	χ²	P value
Age (years)	18~25	188	181 (96.3%)	7 (3.7%)	4.673	0.197
	26~30	746	705 (94.5%)	41 (5.5%)		
	31~35	607	564 (92.9%)	43 (7.1%)		
	≥36	200	192 (96.0%)	8 (4.0%)		
Pre–pregnancy BMI	Underweight (<18.5)	127	119 (93.7%)	8 (6.3%)	1.231	0.746
	Normal (18.5–25)	1152	1090 (94.6%)	62 (5.4%)		
	Overweight (25–30)	264	246 (93.2%)	18 (6.8%)		
	Obese (≥30)	72	69 (95.8%)	3 (4.2%)		
Education level	Primary school or below	14	12 (85.7%)	2 (14.3%)	2.167	0.705
	Junior high school	165	155 (93.9%)	10 (6.1%)		
	High school / vocational school	275	261 (94.9%)	14 (5.1%)		
	College or bachelor's degree	1168	1102 (94.3%)	66 (5.7%)		
	Master's degree or above	119	112 (94.1%)	7 (5.9%)		
Parity	Nulliparous	809	767 (94.8%)	42 (5.2%)	0.536	0.464
	Multiparous	931	874 (93.9%)	57 (6.1%)		
Gestational hypertension	No	1701	1602 (94.2%)	99 (5.8%)	–	0.166
	Yes	40	40 (100.0%)	0 (0.0%)		
Gestational diabetes	No	1616	1522 (94.2%)	94 (5.8%)	0.416	0.519
	Yes	125	120 (96.0%)	5 (4.0%)		
Anemia	No	1623	1528 (94.1%)	95 (5.9%)	0.828	0.363
	Yes	118	114 (96.6%)	4 (3.4%)		
Region	East China	368	344 (93.5%)	24 (6.5%)	11.785	0.038
	North China	569	547 (96.1%)	22 (3.9%)		
	Central China	253	243 (96.0%)	10 (4.0%)		
	South China	37	35 (94.6%)	2 (5.4%)		
	Northeast China	201	182 (90.5%)	19 (9.5%)		
	West China	313	291 (93.0%)	22 (7.0%)		
GDP level	High GDP	797	746 (93.6%)	51 (6.4%)	1.158	0.282
	Low GDP	944	896 (94.9%)	48 (5.1%)		

Table S31. Multivariable Logistic Regression for Calcium, Iron, and Zinc Supplement Use

Variable	Category	Calcium OR (95% CI)	Iron OR (95% CI)	Zinc OR (95% CI)
Age (years)	18~25	0.471 (0.233–0.926)	0.932 (0.554–1.566)	0.843 (0.452–1.554)
	26~30	1.159 (0.614–2.091)	1.309 (0.859–1.974)	1.045 (0.666–1.677)
	31~35	0.722 (0.393–1.263)	1.154 (0.763–1.726)	1.428 (0.926–2.254)
	≥36 (Ref)	1	1	1
Pre–pregnancy BMI	Underweight (<18.5)	1.442 (0.539–3.777)	1.692 (0.874–3.267)	1.253 (0.576–2.867)
	Normal weight(18.5–25)	1.153 (0.512–2.330)	1.908 (1.109–3.226)	1.100 (0.581–2.272)
	Overweight (25–30)	0.966 (0.407–2.108)	1.977 (1.084–3.568)	1.097 (0.546–2.368)
	Obese (≥30) (Ref)	1	1	1
Education level	Primary school or below	0.094 (0.020–0.415)	0.502 (0.123–2.543)	1.195 (0.168–5.429)
	Junior high school	0.636 (0.271–1.427)	0.868 (0.445–1.672)	1.258 (0.644–2.479)
	High school / vocational school	0.677 (0.305–1.408)	0.675 (0.368–1.206)	1.132 (0.620–2.113)
	College or bachelor’s degree	1.162 (0.561–2.205)	0.832 (0.488–1.368)	1.073 (0.651–1.841)
	Master’s degree or above	1	1	1
Parity	Nulliparous	0.973 (0.679–1.396)	1.024 (0.785–1.336)	0.779 (0.583–1.039)
	Multiparous (Ref)	1	1	1
Gestational hypertension	No	0.242 (0.013–1.170)	1.445 (0.605–3.177)	1.110 (0.451–3.348)
	Yes (Ref)	1	1	1
Gestational diabetes	No	1.272 (0.666–2.272)	1.273 (0.807–1.974)	1.378 (0.811–2.489)
	Yes (Ref)	1	1	1
Anemia	No	1.063 (0.519–1.988)	0.196 (0.081–0.402)	1.632 (0.923–3.122)
	Yes (Ref)	1	1	1
Region	North China	0.845 (0.515–1.362)	2.059 (1.429–2.973)	0.543 (0.377–0.781)
	Central China	0.742 (0.422–1.313)	0.534 (0.365–0.779)	0.784 (0.509–1.193)
	South China	4.177 (0.812–76.972)	1.416 (0.582–3.994)	0.763 (0.272–1.841)
	Northeast China	0.870 (0.470–1.641)	1.531 (0.962–2.484)	0.854 (0.532–1.349)
	West China	0.769 (0.445–1.321)	0.574 (0.397–0.827)	0.724 (0.476–1.092)
	East China (Ref)	1	1	1

Table S32. Multivariable Logistic Regression for Fish Oil, Vitamin E, and Vitamin C Supplement Use

Variable	Category	Fish oil OR (95% CI)	Vitamin E OR (95% CI)	Vitamin C OR (95% CI)
Age (years)	18~25	0.651 (0.397–1.062)	0.784 (0.449–1.367)	0.623 (0.384–1.007)
	26~30	0.626 (0.431–0.916)	0.729 (0.473–1.139)	0.864 (0.601–1.246)
	31~35	0.741 (0.514–1.074)	0.793 (0.518–1.230)	0.822 (0.574–1.181)
	≥36 (Ref)	1	1	1
Pre–pregnancy BMI	Underweight (<18.5)	0.803 (0.420–1.549)	0.633 (0.307–1.318)	0.605 (0.320–1.145)
	Normal (18.5–25)	0.777 (0.463–1.338)	0.629 (0.358–1.150)	0.782 (0.469–1.317)
	Overweight (25–30)	0.763 (0.430–1.378)	0.623 (0.332–1.204)	0.646 (0.369–1.141)
	Obese (≥30) (Ref)	1	1	1
Education level	Primary school or below	1.902 (0.441–7.475)	5.750 (1.333–25.112)	5.453 (1.340–27.699)
	Junior high school	1.088 (0.585–2.043)	2.027 (0.984–4.413)	1.444 (0.811–2.598)
	High school / vocational school	1.341 (0.776–2.370)	1.264 (0.630–2.690)	1.246 (0.738–2.137)
	College or bachelor’s degree	1.276 (0.797–2.112)	1.498 (0.822–2.970)	1.451 (0.925–2.330)
	Master’s degree or above (Ref)	1	1	1
Parity	Nulliparous	1.014 (0.792–1.299)	0.884 (0.662–1.177)	0.902 (0.714–1.137)
	Multiparous (Ref)	1	1	1
Gestational hypertension	No	3.117 (1.190–10.734)	1.164 (0.470–3.526)	1.542 (0.714–3.619)
	Yes (Ref)	1	1	1
Gestational diabetes	No	0.655 (0.435–0.999)	1.078 (0.666–1.808)	1.481 (0.971–2.306)
	Yes (Ref)	1	1	1
Anemia	No	1.676 (1.026–2.868)	0.834 (0.504–1.439)	0.618 (0.409–0.938)
	Yes (Ref)	1	1	1
Region	North China	1.274 (0.917–1.781)	1.218 (0.803–1.871)	2.045 (1.515–2.774)
	Central China	1.471 (0.996–2.170)	1.678 (1.037–2.716)	0.816 (0.547–1.207)
	South China	2.428 (1.107–5.183)	1.416 (0.495–3.510)	0.806 (0.323–1.822)
	Northeast China	1.877 (1.235–2.849)	3.583 (2.245–5.766)	3.547 (2.401–5.268)
	West China	1.389 (0.952–2.031)	3.001 (1.954–4.671)	1.630 (1.146–2.322)
	East China (Ref)	1	1	1

Table S33. Multivariable Logistic Regression for Folic Acid, MMS, DHA, and Selenium Supplement Use

Variable	Category	Folic acid OR (95% CI)	MMS OR (95% CI)	DHA OR (95% CI)	Selenium OR (95% CI)
Age (years)	18~25	0.931 (0.489–1.777)	0.521 (0.275–0.965)	0.483 (0.302–0.769)	0.915 (0.302–2.717)
	26~30	1.473 (0.867–2.449)	0.466 (0.295–0.745)	0.675 (0.474–0.962)	1.306 (0.603–3.160)
	31~35	1.503 (0.892–2.480)	0.773 (0.503–1.204)	0.752 (0.531–1.065)	1.798 (0.859–4.251)
	≥36 (Ref)	1	1	1	1
Pre–pregnancy BMI	Underweight (<18.5)	1.261 (0.526–2.934)	1.288 (0.530–3.378)	1.689 (0.902–3.212)	1.342 (0.363–6.420)
	Normal (18.5–25)	1.572 (0.752–3.021)	1.457 (0.706–3.417)	1.371 (0.810–2.374)	1.157 (0.402–4.900)
	Overweight (25–30)	1.622 (0.724–3.438)	0.729 (0.316–1.842)	1.267 (0.716–2.286)	1.499 (0.478–6.624)
	Obese (≥30) (Ref)	1	1	1	1
Education level	Primary school or below	0.499 (0.102–3.686)	0.905 (0.125–4.226)	1.826 (0.462–7.887)	1.469 (0.072–10.382)
	Junior high school	1.066 (0.442–2.511)	0.539 (0.251–1.140)	0.836 (0.483–1.449)	1.280 (0.444–3.865)
	High school / vocational school	0.776 (0.349–1.619)	0.566 (0.292–1.108)	0.859 (0.526–1.407)	0.864 (0.318–2.507)
	College or bachelor’s degree	1.068 (0.518–2.016)	0.858 (0.514–1.486)	1.154 (0.765–1.755)	0.997 (0.457–2.511)
	Master’s degree or above (Ref)	1	1	1	1
Parity	Nulliparous	1.334 (0.932–1.919)	0.941 (0.690–1.283)	1.185 (0.947–1.484)	0.874 (0.541–1.403)
	Multiparous (Ref)	1	1	1	1
Gestational hypertension	No	0.865 (0.202–2.534)	2.610 (0.743–16.627)	2.005 (0.943–4.662)	–
	Yes (Ref)	1	1	1	1
Gestational diabetes	No	0.849 (0.417–1.574)	0.625 (0.385–1.049)	0.753 (0.509–1.117)	1.264 (0.538–3.711)
	Yes (Ref)	1	1	1	1
Anemia	No	1.064 (0.523–1.971)	2.202 (1.135–4.819)	1.063 (0.707–1.611)	1.522 (0.607–5.114)
	Yes (Ref)	1	1	1	1
Region	North China	1.144 (0.726–1.787)	0.872 (0.563–1.358)	1.046 (0.782–1.402)	0.527 (0.280–0.983)
	Central China	1.137 (0.660–1.997)	1.886 (1.188–3.003)	2.035 (1.443–2.876)	0.609 (0.272–1.273)
	South China	1.119 (0.404–3.976)	1.781 (0.620–4.466)	1.358 (0.628–2.854)	0.911 (0.139–3.411)
	Northeast China	1.333 (0.723–2.560)	2.451 (1.489–4.036)	3.783 (2.568–5.621)	1.541 (0.785–2.979)
	West China	1.140 (0.676–1.935)	1.729 (1.091–2.755)	1.546 (1.107–2.164)	0.837 (0.422–1.627)
	East China (Ref)	1	1	1	1

Note: Crude and adjusted odds ratios for any use versus never use. Adjusted for maternal age, prepregnancy BMI, education, geographic region, parity, infant sex, gestational anemia, gestational hypertension, and gestational diabetes.

Table S34. Association Between Maternal Supplement Use and Cesarean Delivery

Supplement	Crude OR	Crude 95% CI	P value	Adjusted OR	Adjusted 95% CI	P value
**Folic Acid**	0.951	0.700-1.292	0.749	0.962	0.689-1.343	0.821
**DHA**	1.247	1.030-1.511	0.024	1.237	1.002-1.526	0.048
**MMS**	1.051	0.807-1.369	0.714	0.993	0.743-1.326	0.960
**Calcium**	0.975	0.718-1.323	0.870	1.058	0.756-1.481	0.742
**Iron**	1.123	0.899-1.404	0.306	1.246	0.971-1.600	0.084
**Zinc**	1.191	0.930-1.527	0.167	1.230	0.940-1.609	0.131
**Selenium**	1.594	1.057-2.429	0.028	1.592	1.020-2.511	0.042
**Fish Oil**	1.398	1.128-1.735	0.002	1.327	1.052-1.673	0.017
**Vitamin E**	1.392	1.091-1.778	0.008	1.337	1.022-1.753	0.035
**Vitamin C**	1.183	0.973-1.441	0.093	1.060	0.853-1.317	0.601

Note: Crude and adjusted odds ratios for any use versus never use. Adjusted for maternal age, prepregnancy BMI, education, geographic region, parity, infant sex, gestational anemia, gestational hypertension, and gestational diabetes.

Table S35. Association Between Maternal Supplement Use and Macrosomia

Supplement	Crude OR	Crude 95% CI	P value	Adjusted OR	Adjusted 95% CI	P value
**Folic Acid**	0.840	0.457-1.699	0.600	0.926	0.451-2.161	0.846
**DHA**	1.066	0.690-1.634	0.769	1.070	0.650-1.743	0.788
**MMS**	0.620	0.287-1.186	0.182	0.622	0.265-1.278	0.230
**Calcium**	0.760	0.421-1.493	0.391	0.769	0.381-1.729	0.491
**Iron**	1.438	0.851-2.583	0.197	1.813	0.953-3.770	0.087
**Zinc**	0.939	0.512-1.611	0.827	0.796	0.396-1.479	0.494
**Selenium**	0.364	0.059-1.176	0.162	0.393	0.062-1.337	0.209
**Fish Oil**	1.371	0.858-2.143	0.175	1.305	0.777-2.146	0.302
**Vitamin E**	1.022	0.576-1.717	0.936	1.233	0.660-2.195	0.492
**Vitamin C**	1.088	0.696-1.677	0.706	1.078	0.650-1.768	0.768

Note: Crude and adjusted odds ratios for any use versus never use. Adjusted for maternal age, prepregnancy BMI, education, geographic region, parity, infant sex, gestational anemia, gestational hypertension, and gestational diabetes.

Table S36. Association Between Maternal Supplement Use and Neonatal Physiological Jaundice

Supplement	Crude OR	Crude 95% CI	P value	Adjusted OR	Adjusted 95% CI	P value
**Folic Acid**	1.584	1.013-2.572	0.052	1.699	1.049-2.870	0.038
**DHA**	1.206	0.934-1.554	0.149	1.317	0.997-1.740	0.052
**MMS**	0.828	0.564-1.195	0.325	0.823	0.544-1.222	0.344
**Calcium**	1.109	0.740-1.701	0.625	1.188	0.769-1.880	0.450
**Iron**	0.909	0.678-1.225	0.524	0.996	0.719-1.391	0.983
**Zinc**	1.134	0.809-1.573	0.458	1.110	0.774-1.574	0.565
**Selenium**	1.260	0.696-2.197	0.427	1.213	0.639-2.208	0.539
**Fish Oil**	1.163	0.876-1.537	0.291	1.147	0.847-1.546	0.371
**Vitamin E**	1.142	0.825-1.566	0.415	1.199	0.840-1.696	0.310
**Vitamin C**	0.983	0.757-1.275	0.900	1.055	0.792-1.402	0.712

Note: Crude and adjusted odds ratios for any use versus never use. Adjusted for maternal age, prepregnancy BMI, education, geographic region, parity, infant sex, gestational anemia, gestational hypertension, and gestational diabetes.

Table S37. Association Between Maternal Supplement Use and Infant Eczema

Supplement	Crude OR	Crude 95% CI	P value	Adjusted OR	Adjusted 95% CI	P value
**Folic Acid**	0.993	0.677-1.469	0.973	0.972	0.646-1.472	0.891
**DHA**	1.048	0.828-1.327	0.695	1.021	0.789-1.321	0.873
**MMS**	0.963	0.687-1.343	0.826	0.934	0.650-1.335	0.712
**Calcium**	1.091	0.748-1.606	0.654	1.153	0.769-1.748	0.496
**Iron**	1.017	0.773-1.342	0.905	1.006	0.743-1.367	0.969
**Zinc**	1.022	0.748-1.392	0.890	1.046	0.749-1.454	0.790
**Selenium**	1.030	0.591-1.768	0.915	1.116	0.620-1.978	0.709
**Fish Oil**	1.178	0.906-1.530	0.220	1.295	0.980-1.710	0.068
**Vitamin E**	1.185	0.881-1.592	0.260	1.274	0.923-1.757	0.139
**Vitamin C**	0.952	0.749-1.210	0.691	0.981	0.754-1.275	0.888

Note: Crude and adjusted odds ratios for any use versus never use. Adjusted for maternal age, prepregnancy BMI, education, geographic region, parity, infant sex, gestational anemia, gestational hypertension, and gestational diabetes.

Table S38. Association Between Supplement Use and Length at Birth

Supplement	1–3 times per week β	P value	FDR-adjusted P	4–7 times per week β	P value	FDR-adjusted P	≥2 times per day β	P value	FDR-adjusted P
**Folic Acid**	0.069	0.663	0.976	0.013	0.926	0.976	-0.012	0.940	0.976
**DHA**	0.036	0.771	0.976	0.051	0.629	0.976	-0.356	0.033	0.615
**MMS**	0.048	0.800	0.976	-0.090	0.585	0.976	-0.159	0.493	0.976
**Calcium**	0.148	0.346	0.976	0.003	0.982	0.982	-0.071	0.648	0.976
**Iron**	0.014	0.911	0.976	0.011	0.926	0.976	-0.061	0.634	0.976
**Zinc**	-0.023	0.888	0.976	0.017	0.921	0.976	0.028	0.889	0.976
**Fish Oil**	0.015	0.915	0.976	0.163	0.210	0.976	-0.158	0.405	0.976
**Vitamin E**	0.328	0.046	0.615	-0.167	0.295	0.976	-0.147	0.493	0.976
**Vitamin C**	0.053	0.677	0.976	-0.177	0.144	0.976	-0.190	0.233	0.976

Note: Adjusted β (frequency levels versus never use). Adjusted for gestational age, maternal age, prepregnancy BMI, education, geographic region, parity, infant sex, gestational anemia, gestational hypertension, and gestational diabetes. FDR within each outcome across supplement×frequency contrasts.

Table S39. Association Between Supplement Use and Length Gain (40-60d)

Supplement	1–3 times per week β	P value	FDR-adjusted P	4–7 times per week β	P value	FDR-adjusted P	≥2 times per day β	P value	FDR-adjusted P
**Folic Acid**	0.969	0.020	0.197	0.684	0.064	0.433	0.969	0.022	0.197
**DHA**	-0.200	0.543	0.956	0.108	0.692	0.956	-0.070	0.874	0.956
**MMS**	-0.136	0.782	0.956	-0.489	0.246	0.853	-0.051	0.937	0.970
**Calcium**	-0.153	0.710	0.956	-0.139	0.718	0.956	-0.100	0.807	0.956
**Iron**	0.228	0.494	0.956	0.043	0.885	0.956	-0.164	0.621	0.956
**Zinc**	0.322	0.432	0.956	-0.018	0.970	0.970	-0.601	0.253	0.853
**Fish Oil**	-0.095	0.786	0.956	0.310	0.355	0.956	-0.206	0.689	0.956
**Vitamin E**	0.131	0.753	0.956	0.674	0.104	0.561	-0.179	0.744	0.956
**Vitamin C**	-0.762	0.018	0.197	0.073	0.814	0.956	-0.450	0.248	0.853

Note: Adjusted β (frequency levels versus never use). Adjusted for the corresponding birth measurement, gestational age, maternal age, prepregnancy BMI, education, geographic region, parity, infant sex, gestational anemia, gestational hypertension, and gestational diabetes. FDR within each outcome across supplement×frequency contrasts.

Table S40. Association Between Supplement Use and Weight at Birth

Supplement	1–3 times/week β	P value	FDR-P	4–7 times/week β	P value	FDR-P	≥2 times/day β	P value	FDR-P
**Folic Acid**	24.533	0.525	0.982	14.262	0.679	0.982	40.512	0.300	0.982
**DHA**	-6.124	0.841	0.982	-1.046	0.968	0.982	23.329	0.566	0.982
**MMS**	22.495	0.627	0.982	-37.144	0.359	0.982	-15.923	0.782	0.982
**Calcium**	16.926	0.658	0.982	-27.511	0.441	0.982	7.261	0.849	0.982
**Iron**	11.492	0.715	0.982	1.014	0.971	0.982	-3.130	0.919	0.982
**Zinc**	-43.647	0.273	0.982	-62.617	0.134	0.982	-1.125	0.982	0.982
**Fish Oil**	3.948	0.909	0.982	-43.243	0.175	0.982	-37.966	0.410	0.982
**Vitamin E**	27.682	0.485	0.982	-56.173	0.153	0.982	8.335	0.876	0.982
**Vitamin C**	-29.014	0.348	0.982	-27.242	0.357	0.982	-24.405	0.524	0.982

Note: Adjusted β (frequency levels versus never use). Adjusted for gestational age, maternal age, prepregnancy BMI, education, geographic region, parity, infant sex, gestational anemia, gestational hypertension, and gestational diabetes. FDR within each outcome across supplement×frequency contrasts.

Table S41. Association Between Supplement Use and Weight Gain (40-60d)

Supplement	1–3 times/week β	P value	FDR-P	4–7 times/week β	P value	FDR-P	≥2 times/day β	P value	FDR-P
**Folic Acid**	113.791	0.362	0.946	33.102	0.766	0.946	87.811	0.488	0.946
**DHA**	-88.051	0.373	0.946	13.810	0.868	0.946	-58.535	0.655	0.946
**MMS**	73.248	0.615	0.946	-33.538	0.794	0.946	123.206	0.519	0.946
**Calcium**	-16.170	0.894	0.946	5.413	0.962	0.962	-77.536	0.521	0.946
**Iron**	-99.810	0.320	0.946	-42.716	0.629	0.946	-182.957	0.064	0.946
**Zinc**	136.971	0.276	0.946	-202.682	0.153	0.946	18.019	0.911	0.946
**Fish Oil**	-65.049	0.539	0.946	28.916	0.774	0.946	-132.130	0.383	0.946
**Vitamin E**	28.556	0.816	0.946	31.265	0.803	0.946	-75.758	0.652	0.946
**Vitamin C**	-116.726	0.226	0.946	-77.349	0.402	0.946	25.501	0.824	0.946

Note: Adjusted β (frequency levels versus never use). Adjusted for the corresponding birth measurement, gestational age, maternal age, prepregnancy BMI, education, geographic region, parity, infant sex, gestational anemia, gestational hypertension, and gestational diabetes. FDR within each outcome across supplement×frequency contrasts.

Table S42. Association Between Supplement Use and Head Circumference at Birth

Supplement	1–3 times/week β	P value	FDR-P	4–7 times/week β	P value	FDR-P	≥2 times/day β	P value	FDR-P
**Folic Acid**	0.046	0.773	0.995	-0.063	0.659	0.995	-0.220	0.175	0.751
**DHA**	0.194	0.120	0.751	0.051	0.630	0.995	-0.195	0.247	0.751
**MMS**	0.078	0.692	0.995	-0.205	0.212	0.751	-0.063	0.775	0.995
**Calcium**	0.288	0.071	0.751	0.065	0.665	0.995	0.061	0.701	0.995
**Iron**	-0.016	0.902	0.995	0.195	0.095	0.751	0.001	0.995	0.995
**Zinc**	-0.105	0.509	0.995	0.011	0.950	0.995	0.219	0.265	0.751
**Fish Oil**	0.155	0.278	0.751	0.189	0.149	0.751	0.026	0.893	0.995
**Vitamin E**	-0.005	0.978	0.995	0.028	0.865	0.995	0.018	0.936	0.995
**Vitamin C**	0.035	0.778	0.995	-0.019	0.879	0.995	-0.197	0.225	0.751

Note: Adjusted β (frequency levels versus never use). Adjusted for gestational age, maternal age, prepregnancy BMI, education, geographic region, parity, infant sex, gestational anemia, gestational hypertension, and gestational diabetes. FDR within each outcome across supplement×frequency contrasts.

Table S43. Association Between Supplement Use and Head Circumference Gain (40-60d)

Supplement	1–3 times/week β	P value	FDR-P	4–7 times/week β	P value	FDR-P	≥2 times/day β	P value	FDR-P
**Folic Acid**	0.291	0.449	0.891	0.518	0.133	0.810	0.405	0.307	0.810
**DHA**	-0.068	0.825	0.891	0.144	0.571	0.891	-0.104	0.798	0.891
**MMS**	-0.936	0.046	0.626	-0.202	0.628	0.891	-0.791	0.207	0.810
**Calcium**	0.385	0.307	0.810	0.731	0.040	0.626	0.398	0.287	0.810
**Iron**	0.154	0.622	0.891	0.469	0.101	0.810	0.091	0.767	0.891
**Zinc**	0.395	0.334	0.810	-0.138	0.771	0.891	-0.503	0.336	0.810
**Fish Oil**	0.176	0.626	0.891	0.371	0.230	0.810	-0.371	0.471	0.891
**Vitamin E**	-0.180	0.651	0.891	0.145	0.716	0.891	0.118	0.815	0.891
**Vitamin C**	-0.022	0.940	0.940	0.042	0.889	0.923	0.332	0.360	0.810

Note: Adjusted β (frequency levels versus never use). Adjusted for the corresponding birth measurement, gestational age, maternal age, prepregnancy BMI, education, geographic region, parity, infant sex, gestational anemia, gestational hypertension, and gestational diabetes. FDR within each outcome across supplement×frequency contrasts.

Table S44. Benjamini–Hochberg False Discovery Rates for Pattern–Outcome Families44

Family	Endpoint	Pattern	Estimate (95% CI)	P	q
Macrosomia (5 tests)	Macrosomia	C1 Mid-late moderate core	0.80 (0.40-1.64)	0.546	0.682
Macrosomia (5 tests)	Macrosomia	C2 Multi-supplement moderate persistent	0.64 (0.19-1.75)	0.411	0.682
Macrosomia (5 tests)	Macrosomia	C3 Multi-supplement high-frequency persistent	1.33 (0.57-3.00)	0.491	0.682
Macrosomia (5 tests)	Macrosomia	C4 All-supplement high-frequency	1.51 (0.57-3.76)	0.385	0.682
Macrosomia (5 tests)	Macrosomia	C5 Mid-late high-frequency core	1.04 (0.49-2.20)	0.909	0.909
Physiological jaundice (5 tests)	Physiological jaundice	C1 Mid-late moderate core	1.33 (0.91-1.95)	0.140	0.234
Physiological jaundice (5 tests)	Physiological jaundice	C2 Multi-supplement moderate persistent	1.06 (0.58-1.90)	0.843	0.843
Physiological jaundice (5 tests)	Physiological jaundice	C3 Multi-supplement high-frequency persistent	1.65 (1.00-2.70)	0.049	0.122
Physiological jaundice (5 tests)	Physiological jaundice	C4 All-supplement high-frequency	1.86 (1.04-3.27)	0.034	0.122
Physiological jaundice (5 tests)	Physiological jaundice	C5 Mid-late high-frequency core	1.24 (0.82-1.88)	0.304	0.380
Infant eczema (5 tests)	Infant eczema	C1 Mid-late moderate core	1.09 (0.78-1.54)	0.615	0.615
Infant eczema (5 tests)	Infant eczema	C2 Multi-supplement moderate persistent	0.58 (0.33-1.01)	0.060	0.300
Infant eczema (5 tests)	Infant eczema	C3 Multi-supplement high-frequency persistent	1.38 (0.87-2.19)	0.165	0.412
Infant eczema (5 tests)	Infant eczema	C4 All-supplement high-frequency	1.29 (0.75-2.21)	0.360	0.471
Infant eczema (5 tests)	Infant eczema	C5 Mid-late high-frequency core	0.84 (0.58-1.23)	0.377	0.471
Any cesarean (5 tests)	Any cesarean	C1 Mid-late moderate core	0.83 (0.62-1.10)	0.186	0.359
Any cesarean (5 tests)	Any cesarean	C2 Multi-supplement moderate persistent	1.15 (0.76-1.76)	0.509	0.636
Any cesarean (5 tests)	Any cesarean	C3 Multi-supplement high-frequency persistent	1.02 (0.69-1.50)	0.913	0.913
Any cesarean (5 tests)	Any cesarean	C4 All-supplement high-frequency	1.33 (0.85-2.11)	0.216	0.359
Any cesarean (5 tests)	Any cesarean	C5 Mid-late high-frequency core	0.72 (0.53-0.99)	0.044	0.221
Birth size (15 tests)	Birth length, cm	C1 Mid-late moderate core	-0.10 (-0.33 to 0.13)	0.382	0.637
Birth size (15 tests)	Birth length, cm	C2 Multi-supplement moderate persistent	-0.02 (-0.36 to 0.32)	0.908	0.908
Birth size (15 tests)	Birth length, cm	C3 Multi-supplement high-frequency persistent	-0.03 (-0.33 to 0.28)	0.870	0.908
Birth size (15 tests)	Birth length, cm	C4 All-supplement high-frequency	-0.46 (-0.81 to -0.10)	0.012	0.175
Birth size (15 tests)	Birth length, cm	C5 Mid-late high-frequency core	-0.06 (-0.32 to 0.20)	0.646	0.869
Birth size (15 tests)	Birth weight, g	C1 Mid-late moderate core	-35.64 (-90.66 to 19.38)	0.204	0.510
Birth size (15 tests)	Birth weight, g	C2 Multi-supplement moderate persistent	-65.14 (-147.21 to 16.93)	0.120	0.449
Birth size (15 tests)	Birth weight, g	C3 Multi-supplement high-frequency persistent	10.07 (-65.01 to 85.14)	0.793	0.908
Birth size (15 tests)	Birth weight, g	C4 All-supplement high-frequency	-41.36 (-128.09 to 45.37)	0.350	0.637
Birth size (15 tests)	Birth weight, g	C5 Mid-late high-frequency core	15.57 (-45.88 to 77.03)	0.619	0.869
Birth size (15 tests)	Birth head circumference, cm	C1 Mid-late moderate core	-0.10 (-0.32 to 0.12)	0.374	0.637
Birth size (15 tests)	Birth head circumference, cm	C2 Multi-supplement moderate persistent	-0.22 (-0.55 to 0.11)	0.196	0.510
Birth size (15 tests)	Birth head circumference, cm	C3 Multi-supplement high-frequency persistent	-0.06 (-0.37 to 0.25)	0.695	0.869
Birth size (15 tests)	Birth head circumference, cm	C4 All-supplement high-frequency	-0.31 (-0.65 to 0.04)	0.083	0.449
Birth size (15 tests)	Birth head circumference, cm	C5 Mid-late high-frequency core	-0.21 (-0.46 to 0.05)	0.109	0.449

Note: Each row compares one non-reference pattern with C6 Low-use in the adjusted model reported in Tables S7–S13. Benjamini–Hochberg adjustment was applied within the family in the first column. Families were macrosomia (5 tests), physiological jaundice (5), infant eczema (5), any cesarean delivery (5), and birth length, birth weight, and birth head circumference together (15). Estimates are adjusted odds ratios for binary endpoints and adjusted β coefficients for birth size. q values are descriptive. No comparison had $q < 0.05$.

Table S45. Associations Between Maternal Supplement-Use Patterns and Cesarean Subtypes45

Cesarean subtype	Pattern	N	Events	Adjusted OR (95% CI)	P	q
Elective, with indication	C1 Mid-late moderate core	1277	485	0.97 (0.70-1.34)	0.834	0.834
Elective, with indication	C2 Multi-supplement moderate persistent	1277	485	1.08 (0.66-1.76)	0.760	0.834
Elective, with indication	C3 Multi-supplement high-frequency persistent	1277	485	1.10 (0.70-1.72)	0.666	0.834
Elective, with indication	C4 All-supplement high-frequency	1277	485	1.60 (0.97-2.64)	0.067	0.334
Elective, with indication	C5 Mid-late high-frequency core	1277	485	0.77 (0.54-1.11)	0.169	0.423
Elective, without indication	C1 Mid-late moderate core	952	160	0.59 (0.36-0.98)	0.041	0.158
Elective, without indication	C2 Multi-supplement moderate persistent	952	160	1.42 (0.73-2.68)	0.293	0.488
Elective, without indication	C3 Multi-supplement high-frequency persistent	952	160	1.07 (0.56-1.97)	0.840	0.840
Elective, without indication	C4 All-supplement high-frequency	952	160	0.77 (0.32-1.74)	0.553	0.691
Elective, without indication	C5 Mid-late high-frequency core	952	160	0.58 (0.32-1.02)	0.063	0.158
Intrapartum or emergency	C1 Mid-late moderate core	911	119	0.80 (0.46-1.40)	0.440	0.787
Intrapartum or emergency	C2 Multi-supplement moderate persistent	911	119	0.99 (0.42-2.20)	0.988	0.988
Intrapartum or emergency	C3 Multi-supplement high-frequency persistent	911	119	0.75 (0.34-1.59)	0.472	0.787
Intrapartum or emergency	C4 All-supplement high-frequency	911	119	1.37 (0.57-3.06)	0.459	0.787
Intrapartum or emergency	C5 Mid-late high-frequency core	911	119	0.89 (0.49-1.62)	0.712	0.890

Note: Each subtype was compared with vaginal delivery (spontaneous or assisted). Women delivered by either of the other cesarean subtypes were excluded from that model, so the sample differs by subtype: elective cesarean with indication, N = 1,277 (485 events); elective cesarean without indication, N = 952 (160 events); intrapartum or emergency cesarean, N = 911 (119 events). Models were adjusted for maternal age, prepregnancy BMI, education, geographic region, parity, infant sex, gestational anemia, gestational hypertension, and gestational diabetes. Reference pattern: C6 Low-use. Benjamini–Hochberg adjustment used the five pattern comparisons within each subtype. No comparison had $q < 0.05$.

Table S46. Model Fit Indices for the Period-Specific Binary Sensitivity Latent Class Analysis46

Classes	BIC	aBIC	Entropy	Minimum class, %	Minimum AvePP	Eligible	Selected
2	40332	40151	0.878	25.1	0.944	Yes	
3	39181	38907	0.817	16.3	0.901	Yes	
4	38473	38108	0.857	12.0	0.913	Yes	
5	38060	37602	0.886	7.0	0.916	Yes	
6	37801	37251	0.875	5.8	0.874	Yes	
7	37642	37000	0.875	5.5	0.850	Yes	
8	37446	36712	0.894	5.5	0.888	Yes	Yes
9	37349	36523	0.896	4.4	0.860	No	
10	37287	36369	0.915	1.8	0.881	No	

Note: Each of seven supplements (folic acid, calcium, iron, DHA, fish oil, vitamin C, and vitamin E) was coded as used or not used in preconception and in each trimester (28 indicators). Frequency did not enter the model. N = 1,679. A model was eligible when entropy was at least 0.70, the smallest class was at least 5%, and the minimum average posterior probability was at least 0.70. Among eligible models, the lowest BIC was at 8 classes (entropy 0.894; minimum class 93 women, 5.5%; minimum average posterior probability 0.888). This solution does not replace the primary six-class model.

Table S47. Cross-Classification of the Primary Six-Class Solution and the Period-Specific Binary Eight-Class Solution47

Primary class	B1	B2	B3	B4	B5	B6	B7	B8	Total
C1 Mid-late moderate core	38	85	17	46	103	107	100	0	496
C2 Multi-supplement moderate persistent	20	5	16	58	14	4	0	23	140
C3 Multi-supplement high-frequency persistent	55	1	8	55	6	0	0	42	167
C4 All-supplement high-frequency	28	6	10	36	8	2	0	28	118
C5 Mid-late high-frequency core	9	56	19	36	42	75	89	0	326
C6 Low-use	8	9	74	5	8	41	287	0	432

Note: Counts of women (N = 1,679). Rows are the primary modal class. Columns B1–B8 are the modal class from the period-specific binary model in Table S46 and Figure S6. Of 432 women in C6 Low-use, 287 (66%) were assigned to B7.

Table S48. Model Fit Indices for the Latent Class Analysis Restricted to Questionnaires Completed on or before Delivery48

Classes	BIC	aBIC	Entropy	Minimum class, %	Minimum AvePP	Eligible	Selected
2	33157	32620	0.955	24.1	0.973	Yes	
3	31401	30594	0.928	20.8	0.966	Yes	
4	31026	29950	0.930	10.9	0.944	Yes	
5	30930	29583	0.921	11.0	0.929	Yes	Yes
6	31030	29414	0.932	4.4	0.940	No	

Note: Questionnaires completed on or before delivery with complete data for the 28 period-by-supplement indicators (folic acid, calcium, iron, DHA, fish oil, vitamin C, and vitamin E in preconception and each trimester). N = 827. A model was eligible when entropy was at least 0.70, the smallest class was at least 5%, and the minimum average posterior probability was at least 0.70. Among eligible models, the lowest BIC was at 5 classes (entropy 0.921; minimum class 91 women, 11.0%; minimum average posterior probability 0.929). The six-class model was not eligible (minimum class 36 women, 4.4%). This solution does not replace the primary six-class model.

Table S49. Classification Diagnostics for the Selected Five-Class Before-Delivery Model49

Class	Label	n	Percent	AvePP	OCC
B1	Mid-to-late moderate use	269	32.5	0.949	39.47
B2	Low-to-moderate, mixed	148	17.9	0.934	65.36
B3	Higher-frequency multi-supplement	91	11.0	0.972	269.78
B4	High-frequency core supplements	139	16.8	0.983	279.33
B5	Low use	180	21.8	0.929	47.10

Note: AvePP = average posterior probability; OCC = odds of correct classification.

Table S50. Association Between Before-Delivery Supplement-Use Classes and Cesarean Delivery50

Class	Adjusted OR	95% CI	P value	q
B1 Mid-to-late moderate use	0.82	0.53–1.25	0.346	0.975
B2 Low-to-moderate, mixed	1.06	0.65–1.74	0.818	0.975
B3 Higher-frequency multi-supplement	1.01	0.58–1.77	0.975	0.975
B4 High-frequency core supplements	1.03	0.63–1.69	0.911	0.975
B5 Low use	1	—	—	—

Note: Adjusted odds ratios (95% CI). Adjusted for maternal age, prepregnancy BMI, education, geographic region, parity, infant sex, gestational anemia, gestational hypertension, and gestational diabetes. Reference = B5 Low use. N = 760 (375 events). Benjamini–Hochberg adjustment used the four class comparisons for this outcome.

Table S51. Association Between Before-Delivery Supplement-Use Classes and Neonatal Physiological Jaundice51

Class	Adjusted OR	95% CI	P value	q
B1 Mid-to-late moderate use	1.28	0.74–2.26	0.379	0.908
B2 Low-to-moderate, mixed	1.01	0.52–1.93	0.981	0.981
B3 Higher-frequency multi-supplement	1.20	0.54–2.59	0.646	0.908
B4 High-frequency core supplements	1.14	0.60–2.17	0.681	0.908
B5 Low use	1	—	—	—

Note: Adjusted odds ratios (95% CI). Adjusted for maternal age, prepregnancy BMI, education, geographic region, parity, infant sex, gestational anemia, gestational hypertension, and gestational diabetes. Reference = B5 Low use. N = 551 (152 events). Benjamini–Hochberg adjustment used the four class comparisons for this outcome.

Table S52. Association Between Before-Delivery Supplement-Use Classes and Length at Birth52

Class	Adjusted β	95% CI	P value	q
B1 Mid-to-late moderate use	0.10	-0.26–0.46	0.576	0.641
B2 Low-to-moderate, mixed	0.11	-0.30–0.53	0.587	0.641
B3 Higher-frequency multi-supplement	0.11	-0.35–0.57	0.641	0.641
B4 High-frequency core supplements	-0.22	-0.64–0.19	0.289	0.641
B5 Low use	0	—	—	—

Note: Adjusted β (95% CI). Adjusted for gestational age, maternal age, prepregnancy BMI, education, geographic region, parity, infant sex, gestational anemia, gestational hypertension, and gestational diabetes. Reference = B5 Low use. N = 744. Benjamini–Hochberg adjustment used the four class comparisons for this outcome.

Table S53. Association Between Before-Delivery Supplement-Use Classes and Head Circumference Gain (40–60 d)53

Class	Adjusted β	95% CI	P value	q
B1 Mid-to-late moderate use	1.19	0.41–1.97	0.003	0.012
B2 Low-to-moderate, mixed	0.42	-0.53–1.36	0.388	0.517
B3 Higher-frequency multi-supplement	0.65	-0.40–1.69	0.225	0.450
B4 High-frequency core supplements	0.15	-0.72–1.02	0.737	0.737
B5 Low use	0	—	—	—

Note: Adjusted β (95% CI). Adjusted for birth head circumference, gestational age, maternal age, prepregnancy BMI, education, geographic region, parity, infant sex, gestational anemia, gestational hypertension, and gestational diabetes. Reference = B5 Low use. N = 279. Benjamini–Hochberg adjustment used the four class comparisons for this outcome.

Table S54. Model Fit Indices for the One-Indicator-per-Product Sensitivity Latent Class Analysis54

Classes	BIC	aBIC	Entropy	Minimum class, %	Minimum AvePP	Eligible	Selected
2	23454	23318	0.880	24.2	0.938	Yes	
3	22539	22333	0.813	23.0	0.909	Yes	
4	22300	22024	0.783	17.7	0.785	Yes	
5	22235	21889	0.772	12.7	0.805	Yes	Yes
6	22257	21841	0.768	7.2	0.808	Yes	

Note: The same 1,679 women and the same seven supplements as the primary analysis (folic acid, calcium, iron, DHA, fish oil, vitamin C, and vitamin E). Each supplement was entered as one frequency score and was not split by period. A model was eligible when entropy was at least 0.70, the smallest class was at least 5%, and the minimum average posterior probability was at least 0.70. Among eligible models, the lowest BIC was at 5 classes (entropy 0.772; minimum class 214 women, 12.7%; minimum average posterior probability 0.805). The six-class solution was eligible but had a higher BIC. This solution does not replace the primary six-class model.

Table S55. Classification Diagnostics for the Selected Five-Class One-Indicator Model55

Class	Label	n	Percent	AvePP	OCC
S1	Lower use	214	12.7	0.836	33.51
S2	Moderate, mixed	336	20.0	0.846	21.85
S3	Higher frequency, all seven products	214	12.7	0.805	27.57
S4	High-frequency folic acid, calcium, and iron	368	21.9	0.926	45.86
S5	Moderate-to-high folic acid, calcium, and iron	547	32.6	0.850	11.80

Note: AvePP = average posterior probability; OCC = odds of correct classification. Mean folic acid frequency in S1 was 1.11.

Table S56. Association Between One-Indicator Supplement-Use Classes and Cesarean Delivery56

Class	Adjusted OR	95% CI	P value	q
S2 Moderate, mixed	1.30	0.89–1.90	0.170	0.678
S3 Higher frequency, all seven products	1.12	0.74–1.69	0.587	0.907
S4 High-frequency folic acid, calcium, and iron	1.07	0.74–1.55	0.736	0.907
S5 Moderate-to-high folic acid, calcium, and iron	0.98	0.69–1.39	0.907	0.907
S1 Lower use	1	—	—	—

Note: Adjusted odds ratios (95% CI). Adjusted for maternal age, prepregnancy BMI, education, geographic region, parity, infant sex, gestational anemia, gestational hypertension, and gestational diabetes. Reference = S1 Lower use. N = 1,556 (764 events). Benjamini–Hochberg adjustment used the four class comparisons for this outcome.

Table S57. Association Between One-Indicator Supplement-Use Classes and Neonatal Physiological Jaundice57

Class	Adjusted OR	95% CI	P value	q
S2 Moderate, mixed	1.02	0.62–1.71	0.926	0.926
S3 Higher frequency, all seven products	1.40	0.82–2.40	0.224	0.620
S4 High-frequency folic acid, calcium, and iron	1.21	0.75–1.99	0.441	0.620
S5 Moderate-to-high folic acid, calcium, and iron	1.19	0.75–1.91	0.465	0.620
S1 Lower use	1	—	—	—

Note: Adjusted odds ratios (95% CI). Adjusted for maternal age, prepregnancy BMI, education, geographic region, parity, infant sex, gestational anemia, gestational hypertension, and gestational diabetes. Reference = S1 Lower use. N = 1,130 (298 events). Benjamini–Hochberg adjustment used the four class comparisons for this outcome.

Table S58. Association Between One-Indicator Supplement-Use Classes and Length at Birth58

Class	Adjusted β	95% CI	P value	q
S2 Moderate, mixed	0.19	-0.11–0.50	0.216	0.865
S3 Higher frequency, all seven products	0.07	-0.27–0.40	0.697	0.929
S4 High-frequency folic acid, calcium, and iron	-0.01	-0.31–0.30	0.973	0.973
S5 Moderate-to-high folic acid, calcium, and iron	0.06	-0.22–0.35	0.658	0.929
S1 Lower use	0	—	—	—

Note: Adjusted β (95% CI). Adjusted for gestational age, maternal age, prepregnancy BMI, education, geographic region, parity, infant sex, gestational anemia, gestational hypertension, and gestational diabetes. Reference = S1 Lower use. N = 1,445. Benjamini–Hochberg adjustment used the four class comparisons for this outcome.

Table S59. Association Between One-Indicator Supplement-Use Classes and Head Circumference Gain (40–60 d)59

Class	Adjusted β	95% CI	P value	q
S2 Moderate, mixed	0.56	-0.18–1.30	0.140	0.186
S3 Higher frequency, all seven products	0.71	-0.09–1.51	0.082	0.165
S4 High-frequency folic acid, calcium, and iron	0.38	-0.35–1.10	0.306	0.306
S5 Moderate-to-high folic acid, calcium, and iron	0.75	0.07–1.42	0.031	0.122
S1 Lower use	0	—	—	—

Note: Adjusted β (95% CI). Adjusted for birth head circumference, gestational age, maternal age, prepregnancy BMI, education, geographic region, parity, infant sex, gestational anemia, gestational hypertension, and gestational diabetes. Reference = S1 Lower use. N = 513. Benjamini–Hochberg adjustment used the four class comparisons for this outcome.

Table S60. Association Between One-Indicator Supplement-Use Classes and Length Gain (40–60 d)60

Class	Adjusted β	95% CI	P value	q
S2 Moderate, mixed	0.48	-0.32–1.28	0.240	0.388
S3 Higher frequency, all seven products	0.49	-0.38–1.36	0.271	0.388
S4 High-frequency folic acid, calcium, and iron	0.42	-0.38–1.21	0.304	0.388
S5 Moderate-to-high folic acid, calcium, and iron	0.33	-0.42–1.08	0.388	0.388
S1 Lower use	0	—	—	—

Note: Adjusted β (95% CI). Adjusted for birth length, gestational age, maternal age, prepregnancy BMI, education, geographic region, parity, infant sex, gestational anemia, gestational hypertension, and gestational diabetes. Reference = S1 Lower use. N = 965. Benjamini–Hochberg adjustment used the four class comparisons for this outcome.

Table S61. Association Between One-Indicator Supplement-Use Classes and Weight Gain (40–60 d)61

Class	Adjusted β	95% CI	P value	q
S2 Moderate, mixed	-7.81	-243.63–228.01	0.948	0.962
S3 Higher frequency, all seven products	10.76	-245.53–267.04	0.934	0.962
S4 High-frequency folic acid, calcium, and iron	-44.95	-276.67–186.78	0.704	0.962
S5 Moderate-to-high folic acid, calcium, and iron	-5.37	-225.71–214.97	0.962	0.962
S1 Lower use	0	—	—	—

Note: Adjusted β (95% CI). Adjusted for birth weight, gestational age, maternal age, prepregnancy BMI, education, geographic region, parity, infant sex, gestational anemia, gestational hypertension, and gestational diabetes. Reference = S1 Lower use. N = 1,036. Benjamini–Hochberg adjustment used the four class comparisons for this outcome.

Table S62. Logistic Model for Completion of the 40–60-Day Follow-up Used to Derive Inverse-Probability Weights62

Covariate	Odds ratio	95% CI	P value
Intercept (reference odds)	2.17	1.32–3.54	0.002
North China	2.43	1.67–3.54	<0.001
Central China	2.06	1.29–3.27	0.002
South China	0.01	0.00–0.07	<0.001
Northeast China	5.99	2.89–12.41	<0.001
West China	1.01	0.69–1.49	0.952
Age 18–25 years	0.80	0.50–1.27	0.339
Age 31–35 years	1.11	0.80–1.55	0.520
Age ≥36 years	0.65	0.42–1.01	0.054
Underweight	1.47	0.87–2.51	0.154
Overweight	1.77	1.13–2.77	0.013
Obese	0.61	0.33–1.12	0.112
High school or vocational school	1.06	0.70–1.59	0.793
Junior high school	1.09	0.65–1.83	0.732
Master's degree or above	1.40	0.78–2.52	0.265
Primary school or below	0.31	0.07–1.44	0.135
Any folic acid use	1.47	0.97–2.23	0.066

Note: Logistic regression for completion of the 40–60-day follow-up. N = 1,623 with complete covariates (1,341 completed follow-up). Reference = East China, age 26–30 years, normal prepregnancy BMI, college or a bachelor's degree, and no folic acid use. The intercept is the odds of follow-up in the reference group; other rows are odds ratios. Stabilized weight = overall follow-up rate / predicted probability. One of 34 women from South China completed follow-up (stabilized weight 23.9) and did not contribute a 40–60-day length, weight, or head circumference.

Table S63. Follow-up-Weighted Association Between Maternal Supplement-Use Patterns and Neonatal Physiological Jaundice63

Pattern	Adjusted OR	95% CI	P value	q
C1 Mid-late moderate core	1.31	0.89–1.92	0.172	0.287
C2 Multi-supplement moderate	1.08	0.59–1.99	0.793	0.793
C3 Multi-supplement high-frequency	1.57	0.96–2.57	0.069	0.173
C4 Full-course high-frequency	1.80	1.01–3.22	0.047	0.173
C5 Mid-late high-frequency core	1.23	0.81–1.86	0.332	0.415
C6 Low use	1	—	—	—

Note: Adjusted odds ratios (95% CI), weighted by stabilized inverse-probability weights for completion of the 40–60-day follow-up. Classes were the primary six-class solution. Adjusted for maternal age, prepregnancy BMI, education, geographic region, parity, infant sex, gestational anemia, gestational hypertension, and gestational diabetes. Reference = C6 Low-use. N = 1,130 (298 events). Benjamini–Hochberg adjustment used the five pattern comparisons for this outcome.

Table S64. Follow-up-Weighted Association Between Maternal Supplement-Use Patterns and Length Gain (40–60 d)64

Pattern	Adjusted β	95% CI	P value	q
C1 Mid-late moderate core	0.19	-0.38–0.77	0.511	0.635
C2 Multi-supplement moderate	0.24	-0.75–1.23	0.635	0.635
C3 Multi-supplement high-frequency	0.21	-0.59–1.00	0.611	0.635
C4 Full-course high-frequency	-0.52	-1.41–0.37	0.249	0.635
C5 Mid-late high-frequency core	0.25	-0.39–0.89	0.449	0.635
C6 Low use	0	—	—	—

Note: Adjusted β (95% CI), weighted by stabilized inverse-probability weights for completion of the 40–60-day follow-up. Classes were the primary six-class solution. Adjusted for birth length, gestational age, maternal age, prepregnancy BMI, education, geographic region, parity, infant sex, gestational anemia, gestational hypertension, and gestational diabetes. Reference = C6 Low-use. N = 965. Benjamini–Hochberg adjustment used the 15 growth comparisons (length, weight, and head-circumference gain).

Table S65. Follow-up-Weighted Association Between Maternal Supplement-Use Patterns and Weight Gain (40–60 d)65

Pattern	Adjusted β	95% CI	P value	q
C1 Mid-late moderate core	113.18	-58.83–285.18	0.197	0.635
C2 Multi-supplement moderate	109.81	-156.32–375.94	0.418	0.635
C3 Multi-supplement high-frequency	66.41	-154.60–287.41	0.556	0.635
C4 Full-course high-frequency	-72.70	-342.49–197.09	0.597	0.635
C5 Mid-late high-frequency core	47.77	-132.11–227.65	0.602	0.635
C6 Low use	0	—	—	—

Note: Adjusted β (95% CI), weighted by stabilized inverse-probability weights for completion of the 40–60-day follow-up. Classes were the primary six-class solution. Adjusted for birth weight, gestational age, maternal age, prepregnancy BMI, education, geographic region, parity, infant sex, gestational anemia, gestational hypertension, and gestational diabetes. Reference = C6 Low-use. N = 1,036. Benjamini–Hochberg adjustment used the 15 growth comparisons (length, weight, and head-circumference gain).

Table S66. Follow-up-Weighted Association Between Maternal Supplement-Use Patterns and Head Circumference Gain (40–60 d)66

Pattern	Adjusted β	95% CI	P value	q
C1 Mid-late moderate core	0.83	0.27–1.40	0.004	0.056
C2 Multi-supplement moderate	0.48	-0.41–1.37	0.292	0.635
C3 Multi-supplement high-frequency	0.85	0.19–1.52	0.012	0.087
C4 Full-course high-frequency	0.45	-0.43–1.34	0.311	0.635
C5 Mid-late high-frequency core	0.39	-0.16–0.93	0.165	0.635
C6 Low use	0	—	—	—

Note: Adjusted β (95% CI), weighted by stabilized inverse-probability weights for completion of the 40–60-day follow-up. Classes were the primary six-class solution. Adjusted for birth head circumference, gestational age, maternal age, prepregnancy BMI, education, geographic region, parity, infant sex, gestational anemia, gestational hypertension, and gestational diabetes. Reference = C6 Low-use. N = 513. Benjamini–Hochberg adjustment used the 15 growth comparisons (length, weight, and head-circumference gain).

Table S67. Model Fit Indices for the Latent Class Analysis Excluding Preterm Births67

Classes	BIC	aBIC	Entropy	Minimum class, %	Minimum AvePP	Eligible	Selected
2	64568	64031	0.946	25.0	0.973	Yes	
3	60768	59961	0.922	21.5	0.961	Yes	
4	59710	58633	0.928	10.4	0.950	Yes	
5	59113	57766	0.923	8.2	0.943	Yes	
6	58760	57143	0.929	7.0	0.945	Yes	Yes
7	58813	56926	0.922	4.4	0.899	No	
8	58827	56670	0.942	3.8	0.940	No	
9	58968	56541	0.928	2.3	0.907	No	
10	59033	56336	0.940	2.9	0.911	No	

Note: Preterm birth was defined as delivery before 37 completed weeks. Twelve preterm infants were excluded. The same 28 period-by-supplement indicators were used (folic acid, calcium, iron, DHA, fish oil, vitamin C, and vitamin E in preconception and each trimester). N = 1,667. A model was eligible when entropy was at least 0.70, the smallest class was at least 5%, and the minimum average posterior probability was at least 0.70. Among eligible models, the lowest BIC was at 6 classes (entropy 0.929; minimum class 117 women, 7.0%; minimum average posterior probability 0.945). Models with 7–10 classes were not eligible because the smallest class was below 5%. Of the 1,667 women retained, 1,661 (99.6%) were assigned to the same class as in the primary six-class solution. This solution does not replace the primary six-class model.

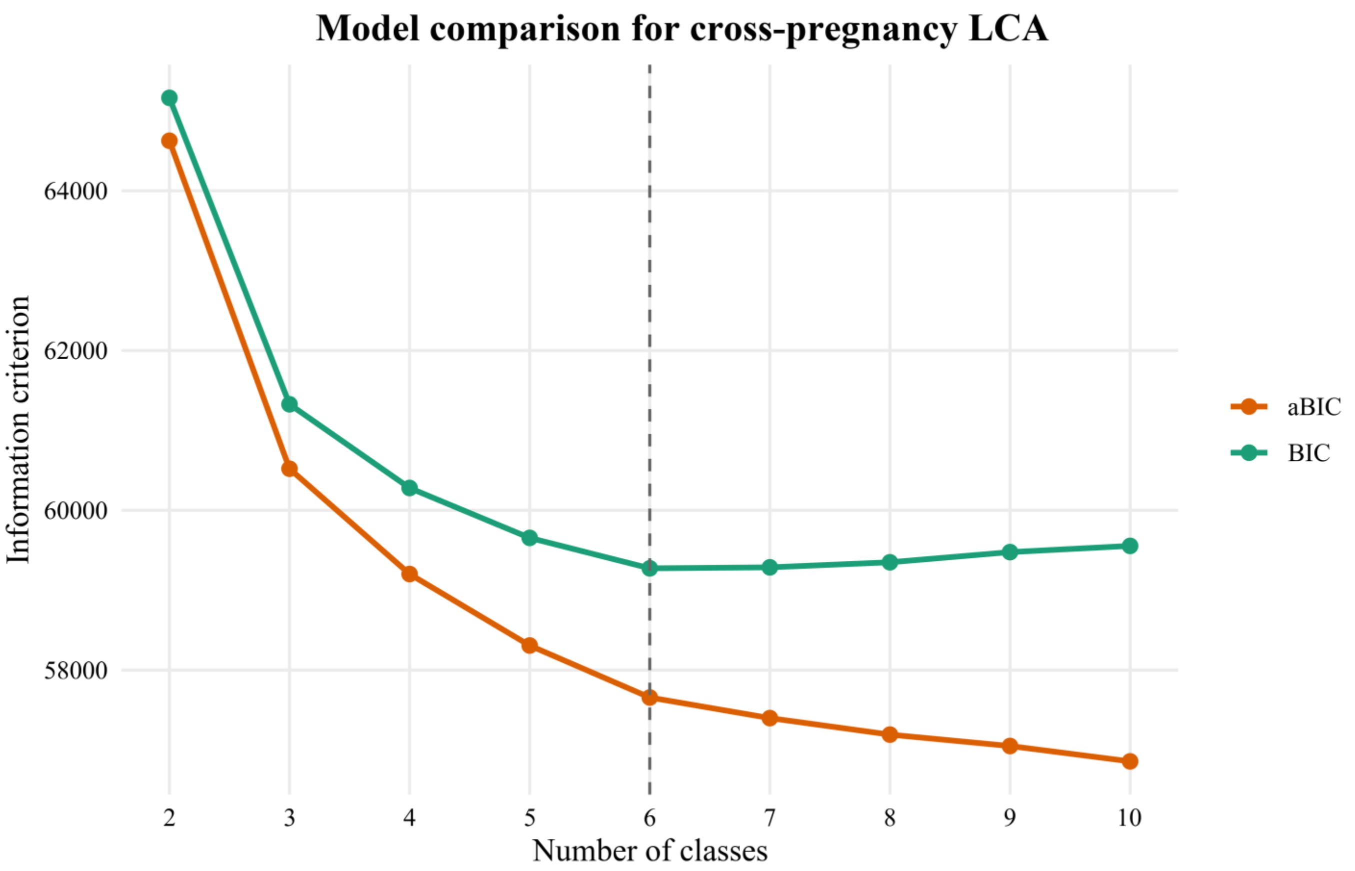

Figure S1. Model comparison for the cross-pregnancy latent class analysis. Bayesian information criterion (BIC) and sample-size-adjusted BIC (aBIC) are shown for models with 2–10 classes fitted to 28 period-by-supplement frequency indicators (N = 1,679). The dashed line marks the selected six-class model, which had the lowest BIC among models with relative entropy ≥ 0.70, a minimum class size ≥ 5%, and a minimum average posterior probability ≥ 0.70.

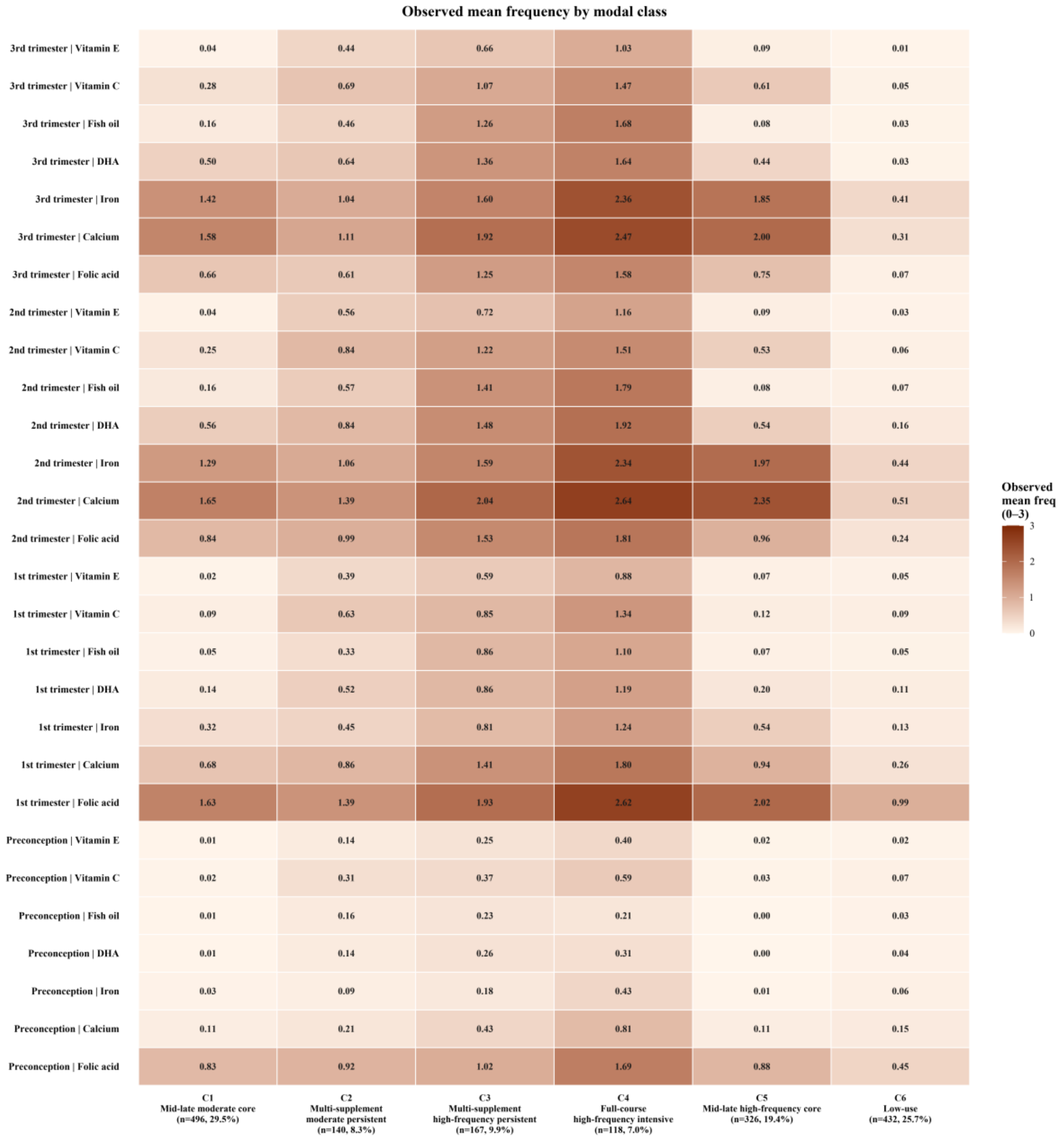
Observed mean frequency by modal class
3rd trimester | Vitamin E 0.04 0.44 0.66 1.03 0.09 0.01
3rd trimester | Vitamin C 0.28 0.69 1.07 1.47 0.61 0.05
3rd trimester | Fish oil 0.16 0.46 1.26 1.68 0.08 0.03
3rd trimester | DHA 0.50 0.64 1.36 1.64 0.44 0.03
3rd trimester | Iron 1.42 1.04 1.60 2.36 1.85 0.41
3rd trimester | Calcium 1.58 1.11 1.92 2.47 2.00 0.31
3rd trimester | Folic acid 0.66 0.61 1.25 1.58 0.75 0.07
2nd trimester | Vitamin E 0.04 0.56 0.72 1.16 0.09 0.03
2nd trimester | Vitamin C 0.25 0.84 1.22 1.51 0.53 0.06
2nd trimester | Fish oil 0.16 0.57 1.41 1.79 0.08 0.07
2nd trimester | DHA 0.56 0.84 1.48 1.92 0.54 0.16
2nd trimester | Iron 1.29 1.06 1.59 2.34 1.97 0.44
2nd trimester | Calcium 1.65 1.39 2.04 2.64 2.35 0.51
2nd trimester | Folic acid 0.84 0.99 1.53 1.81 0.96 0.24
1st trimester | Vitamin E 0.02 0.39 0.59 0.88 0.07 0.05
1st trimester | Vitamin C 0.09 0.63 0.85 1.34 0.12 0.09
1st trimester | Fish oil 0.05 0.33 0.86 1.10 0.07 0.05
1st trimester | DHA 0.14 0.52 0.86 1.19 0.20 0.11
1st trimester | Iron 0.32 0.45 0.81 1.24 0.54 0.13
1st trimester | Calcium 0.68 0.86 1.41 1.80 0.94 0.26
1st trimester | Folic acid 1.63 1.39 1.93 2.62 2.02 0.99
Preconception | Vitamin E 0.01 0.14 0.25 0.40 0.02 0.02
Preconception | Vitamin C 0.02 0.31 0.37 0.59 0.03 0.07
Preconception | Fish oil 0.01 0.16 0.23 0.21 0.00 0.03
Preconception | DHA 0.01 0.14 0.26 0.31 0.00 0.04
Preconception | Iron 0.03 0.09 0.18 0.43 0.01 0.06
Preconception | Calcium 0.11 0.21 0.43 0.81 0.11 0.15
Preconception | Folic acid 0.83 0.92 1.02 1.69 0.88 0.45
C1 Mid-late moderate core (n=496, 29.5%)
C2 Multi-supplement moderate persistent (n=140, 8.3%)
C3 Multi-supplement high-frequency persistent (n=167, 9.9%)
C4 Full-course high-frequency intensive (n=118, 7.0%)
C5 Mid-late high-frequency core (n=326, 19.4%)
C6 Low-use (n=432, 25.7%)
Observed mean freq (0–3)
3
2
1
0

Figure S2. Observed mean supplement-use frequency by latent class. Each cell is the mean frequency score among women assigned to that class. Rows are pregnancy period and supplement; columns are the six classes, labelled with the pattern name and the number and percentage of women. Scores are 0 = none, 1 = 1–3 times/week, 2 = 4–7 times/week, and 3 = ≥2 times/day.

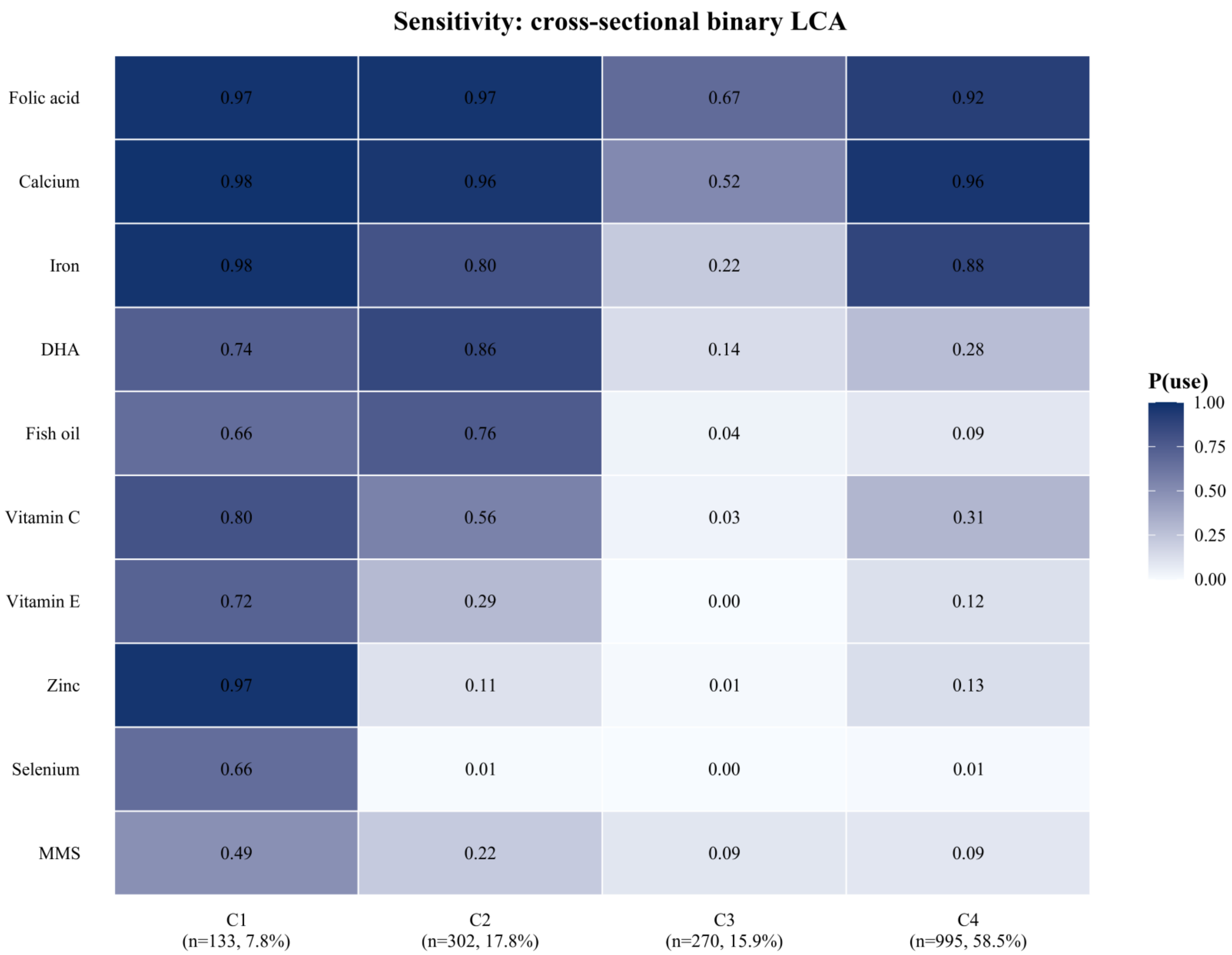
Sensitivity: cross-sectional binary LCA
Folic acid
Calcium
Iron
DHA
Fish oil
Vitamin C
Vitamin E
Zinc
Selenium
MMS
0.97 0.97 0.67 0.92
0.98 0.96 0.52 0.96
0.98 0.80 0.22 0.88
0.74 0.86 0.14 0.28
0.66 0.76 0.04 0.09
0.80 0.56 0.03 0.31
0.72 0.29 0.00 0.12
0.97 0.11 0.01 0.13
0.66 0.01 0.00 0.01
0.49 0.22 0.09 0.09
C1 (n=133, 7.8%)
C2 (n=302, 17.8%)
C3 (n=270, 15.9%)
C4 (n=995, 58.5%)
P(use)
1.00
0.75
0.50
0.25
0.00

Figure S3. Sensitivity latent class analysis using cross-sectional binary indicators. Folic acid, calcium, iron, DHA, fish oil, vitamin C, vitamin E, zinc, selenium, and multiple micronutrient supplements were each coded as any use versus none, without separation by pregnancy period (N = 1,700). A four-class model was selected. Cell values are model-estimated probabilities of use.

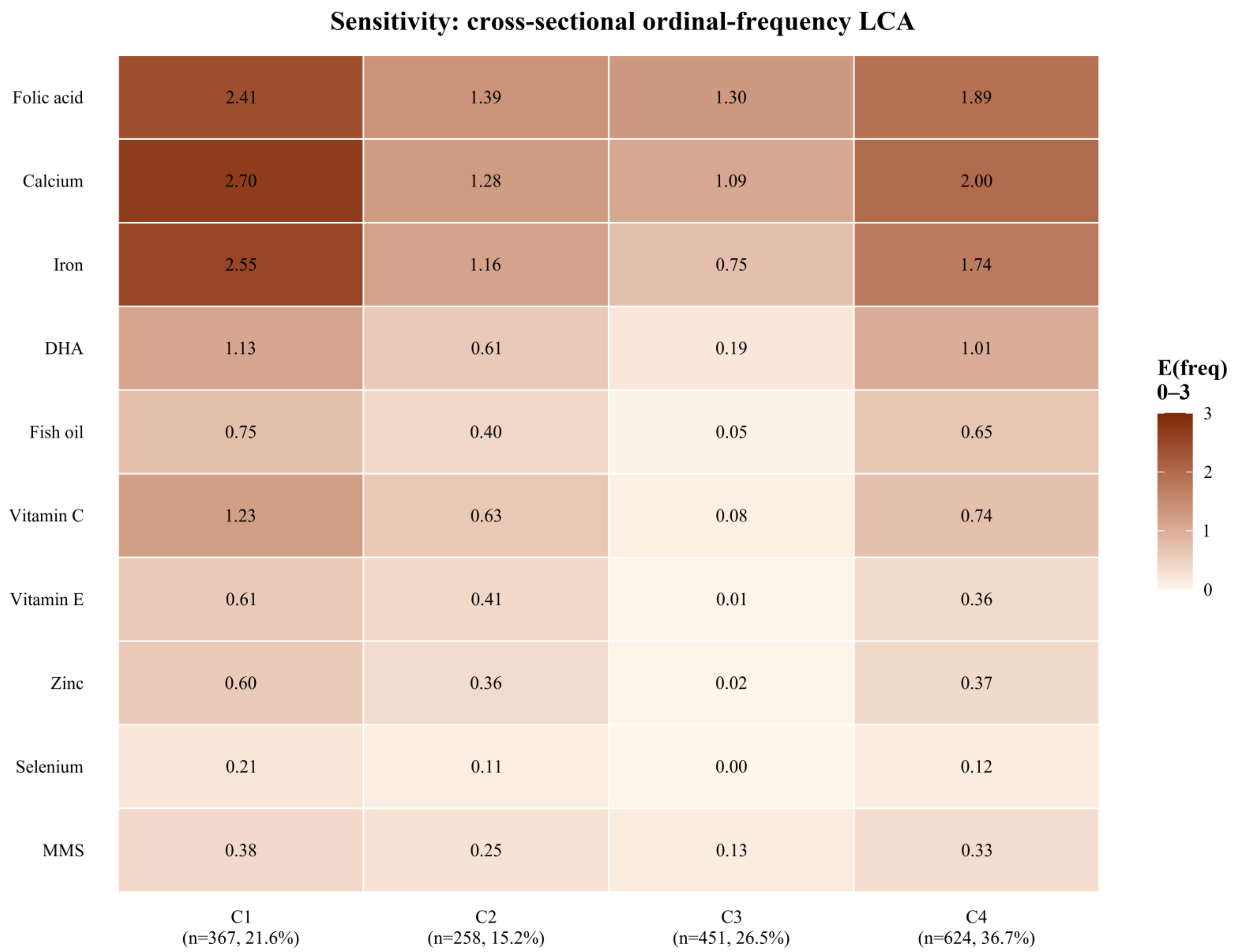
Sensitivity: cross-sectional ordinal-frequency LCA
Folic acid
Calcium
Iron
DHA
Fish oil
Vitamin C
Vitamin E
Zinc
Selenium
MMS
2.41
1.39
1.30
1.89
2.70
1.28
1.09
2.00
2.55
1.16
0.75
1.74
1.13
0.61
0.19
1.01
0.75
0.40
0.05
0.65
1.23
0.63
0.08
0.74
0.61
0.41
0.01
0.36
0.60
0.36
0.02
0.37
0.21
0.11
0.00
0.12
0.38
0.25
0.13
0.33
C1
(n=367, 21.6%)
C2
(n=258, 15.2%)
C3
(n=451, 26.5%)
C4
(n=624, 36.7%)
E(freq)
0–3
3
2
1
0

Figure S4. Sensitivity latent class analysis using cross-sectional frequency indicators. Folic acid, calcium, iron, DHA, fish oil, vitamin C, vitamin E, zinc, selenium, and multiple micronutrient supplements were each entered as a four-level variable, without separation by pregnancy period (N = 1,700): 0 = none, 1 = 1–3 times/week, 2 = 4–7 times/week, and 3 = ≥2 times/day. A four-class model was selected. Cell values are model-expected frequency scores.

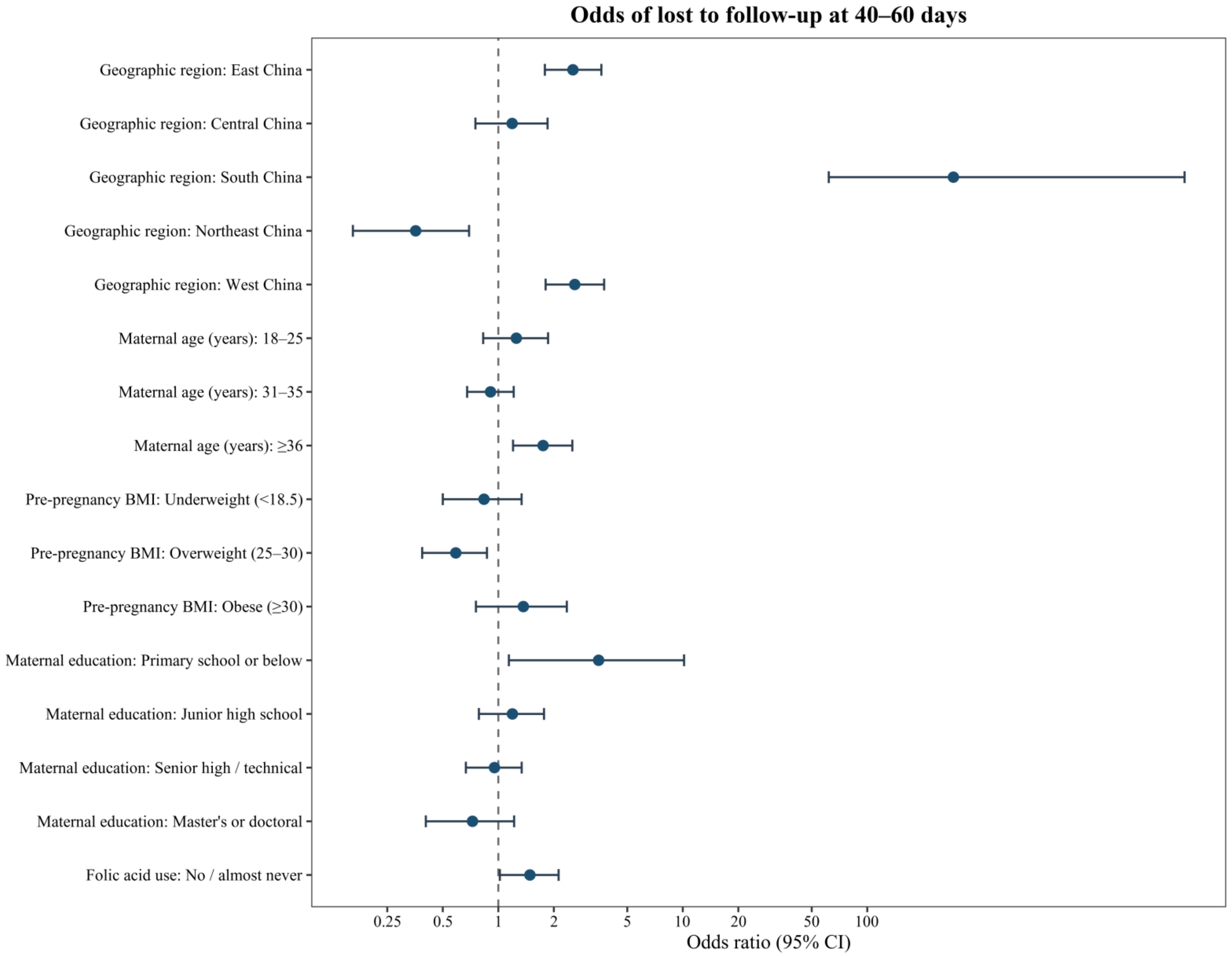
Odds of lost to follow-up at 40–60 days
Geographic region: East China
Geographic region: Central China
Geographic region: South China
Geographic region: Northeast China
Geographic region: West China
Maternal age (years): 18–25
Maternal age (years): 31–35
Maternal age (years): ≥36
Pre-pregnancy BMI: Underweight (<18.5)
Pre-pregnancy BMI: Overweight (25–30)
Pre-pregnancy BMI: Obese (≥30)
Maternal education: Primary school or below
Maternal education: Junior high school
Maternal education: Senior high / technical
Maternal education: Master's or doctoral
Folic acid use: No / almost never
0.25
0.5
1
2
5
10
20
50
100
Odds ratio (95% CI)

Figure S5. Odds of loss to follow-up at 40–60 days after birth. Points are univariable odds ratios and horizontal bars are 95% confidence intervals, on a logarithmic scale, comparing women lost to follow-up with those who completed the assessment. The vertical line is an odds ratio of 1. Reference categories are North China, maternal age 26–30 years, normal prepregnancy body-mass index, a college or bachelor's degree, and any use of folic acid.

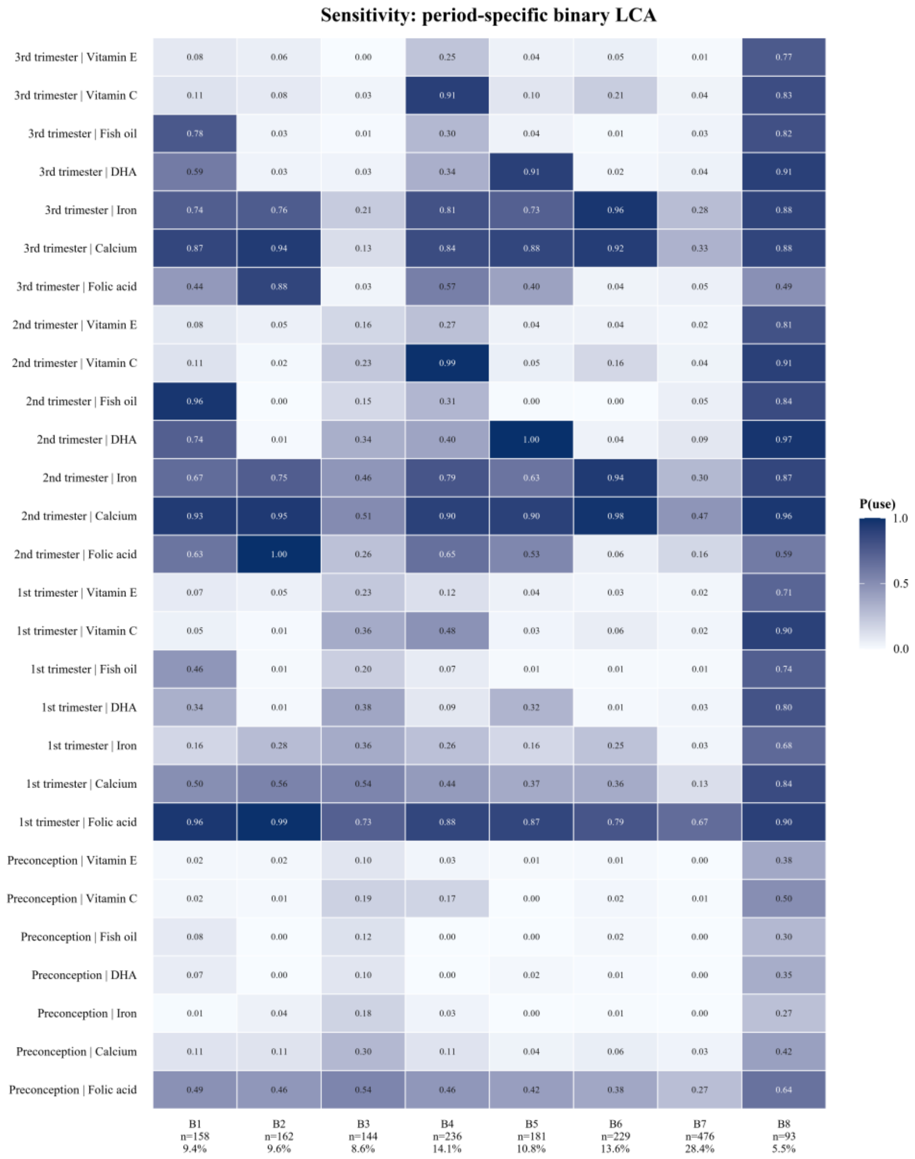
Sensitivity: period-specific binary LCA
3rd trimester | Vitamin E 0.08 0.06 0.00 0.25 0.04 0.05 0.01 0.77
3rd trimester | Vitamin C 0.11 0.08 0.03 0.91 0.10 0.21 0.04 0.83
3rd trimester | Fish oil 0.78 0.03 0.01 0.30 0.04 0.01 0.03 0.82
3rd trimester | DHA 0.59 0.03 0.03 0.34 0.91 0.02 0.04 0.91
3rd trimester | Iron 0.74 0.76 0.21 0.81 0.73 0.96 0.28 0.88
3rd trimester | Calcium 0.87 0.94 0.13 0.84 0.88 0.92 0.33 0.88
3rd trimester | Folic acid 0.44 0.88 0.03 0.57 0.40 0.04 0.05 0.49
2nd trimester | Vitamin E 0.08 0.05 0.16 0.27 0.04 0.04 0.02 0.81
2nd trimester | Vitamin C 0.11 0.02 0.23 0.99 0.05 0.16 0.04 0.91
2nd trimester | Fish oil 0.96 0.00 0.15 0.31 0.00 0.00 0.05 0.84
2nd trimester | DHA 0.74 0.01 0.34 0.40 1.00 0.04 0.09 0.97
2nd trimester | Iron 0.67 0.75 0.46 0.79 0.63 0.94 0.30 0.87
2nd trimester | Calcium 0.93 0.95 0.51 0.90 0.90 0.98 0.47 0.96
2nd trimester | Folic acid 0.63 1.00 0.26 0.65 0.53 0.06 0.16 0.59
1st trimester | Vitamin E 0.07 0.05 0.23 0.12 0.04 0.03 0.02 0.71
1st trimester | Vitamin C 0.05 0.01 0.36 0.48 0.03 0.06 0.02 0.90
1st trimester | Fish oil 0.46 0.01 0.20 0.07 0.01 0.01 0.01 0.74
1st trimester | DHA 0.34 0.01 0.38 0.09 0.32 0.01 0.03 0.80
1st trimester | Iron 0.16 0.28 0.36 0.26 0.16 0.25 0.03 0.68
1st trimester | Calcium 0.50 0.56 0.54 0.44 0.37 0.36 0.13 0.84
1st trimester | Folic acid 0.96 0.99 0.73 0.88 0.87 0.79 0.67 0.90
Preconception | Vitamin E 0.02 0.02 0.10 0.03 0.01 0.01 0.00 0.38
Preconception | Vitamin C 0.02 0.01 0.19 0.17 0.00 0.02 0.01 0.50
Preconception | Fish oil 0.08 0.00 0.12 0.00 0.00 0.02 0.00 0.30
Preconception | DHA 0.07 0.00 0.10 0.00 0.02 0.01 0.00 0.35
Preconception | Iron 0.01 0.04 0.18 0.03 0.00 0.01 0.00 0.27
Preconception | Calcium 0.11 0.11 0.30 0.11 0.04 0.06 0.03 0.42
Preconception | Folic acid 0.49 0.46 0.54 0.46 0.42 0.38 0.27 0.64
B1 n=158 9.4%
B2 n=162 9.6%
B3 n=144 8.6%
B4 n=236 14.1%
B5 n=181 10.8%
B6 n=229 13.6%
B7 n=476 28.4%
B8 n=93 5.5%
P(use)
1.0
0.5
0.0

Figure S6. Sensitivity latent class analysis using period-specific binary indicators. Folic acid, calcium, iron, DHA, fish oil, vitamin C, and vitamin E were each coded as used or not used in preconception and in the first, second, and third trimesters (28 indicators). Frequency did not enter the class model. Cells are the model-estimated probability of use in that period. Columns are the eight-class solution selected by the same rules as the primary analysis (entropy ≥ 0.70, minimum class size ≥ 5%, minimum average posterior probability ≥ 0.70, then lowest BIC; N = 1,679; entropy 0.894). Labels B1–B8 refer only to this sensitivity solution and are not classes C1–C6. The number and percentage of women are shown under each column.6
